# A new species of *Impatiens* and updated checklist of Balsaminaceae in Nepal

**DOI:** 10.1371/journal.pone.0274699

**Published:** 2022-10-19

**Authors:** Bhakta Bahadur Raskoti, Rita Ale

**Affiliations:** Nature Research and Conservation Initiative, Banke, Nepal; Public Library of Science, UNITED KINGDOM

## Abstract

*Impatiens nimspurjae* (*Impatiens*, Balsaminaceae), a new species from Nepal is described based on molecular data and morphological characters. Notes on the diagnostic characters used to distinguish it from allied taxa are provided. *I*. *nimspurjae* is closely related with taxon belonging to sect. *Racemosae* (*I*. *harae*, *I*. *radiata*, *I*. *wallichii*, *I*. *urticifolia*) in having many-flowered racemose inflorescences, lateral sepals 2 (very rarely 4 with inner 2 reduced), capsule linear, seed ovoid, but differs by its sessile leaves, upper lobe of lower united petal not truncated, spur flattened at base. With the discovery of this new species and five species new records to Nepal, a checklist of Balsaminaceae having 57 species (8 endemic) in Nepal is updated. An identification key to the species of *Imaptiens* in Nepal is also provided.

## Introduction

The genus *Impatiens* L. is one of the largest genera of flowering plants, containing over 1000 species, which exhibit complex pattern of morphological variation [1, 2]. It is widely distributed in tropical, subtropical, temperate and alpine regions of the Old World, with the greatest diversity seen in tropical Africa, Madagascar, southern India, Sri Lanka, Himalayas and Southeast Asia [3–5]. Biogeographically, *Impatiens* species are of Southeast Asian origin from where it dispersed to boreal Eurasia and North America to central Asia and eastern Europe via the Himalayas to India and Africa [4]. Based on the combined molecular and morphological phylogeny, *Impatiens* is divided into two subgenera, *Clavicarpa* and *Impatiens*; the latter is further subdivided into different sections [6].

Most of the *Impatiens* species in Nepal are restricted to eastern and central Nepal [7, 8]. Various authors studied Balsaminaceae of Nepal targeting on some problematic species group [9–11]. However, *Impatiens* is still not well studied and documented within Nepal. During different botanical surveys in the various parts of Nepal from 2007 to 2012, some unidentified plants of *Impatiens* were collected. We carefully studied taxonomic characters of these plants and also performed a molecular phylogeny of *Impatiens* to understand systematic position of a newly collected plant.

## Materials and methods

### Ethics statement

The collecting location reported in this work is not protected area by any law. In addition, the plant species collected here are currently neither endangered nor protected. No specific permits were required for this study.

### Nomenclature

The electronic version of this article in Portable Document Format (PDF) in a work with an ISSN and ISBN will represent a published work according to the International Code of Nomenclature for algae, fungi, and plants, and hence the new names contained in the electronic publication of PLoS article are effectively published under that Code from the electronic edition alone, so there is no longer any need to provide printed copies.

In addition, new name contained in this work has been submitted to The International Plant Names Index (IPNI), from where they will be made available to the Global Names Index (http://gni.globalnames.org/). The IPNI LSIDs can be resolved and the associated information viewed through any standard web browser by appending the LSID contained in this publication to the prefix http://ipni.org/. The online version of this work is archived and available from the following digital repositories: PubMed Central and LOCKSS.

### Morphological observations

Vegetative and reproductive characters were studied based on fresh and preserved specimens. Characters such as phyllotaxy, petiole, leaf shape, bracts, sepals, petals, androecium, gynoecium and fruits were carefully examined and measured. The characters were compared with related species deposited in different herbaria, and their description published in different literatures. The plants were dried and herbarium specimens were prepared following the standard procedure and deposited them in the National Herbarium and Plant Laboratories (KATH), Godawari, Lalitpur, Nepal and Tribhuvan University Central Herbarium (TUCH).

### Taxon sampling

In total, 239 nucleotide sequences of 238 species of *Impatiens* were sampled in the phylogenetic study. We downloaded 236 sequences of 236 species from GenBank. Additionally, we sequenced three samples of the newly collected taxon for the present analyses. The ingroup taxa included a total of 236 sequences of 235 species, representing all of the major clades in the *Impatiens* [6, 12]. Three species namely *Marcgravia umbellata*, *Norantea guianensis* and *Hydrocera triflora* were selected as outgroups following previous phylogenetic studies [6, 12]. A list of sampled species, GenBank accession numbers for all sequences used in this study are available in S1 Table.

### DNA extraction and marker selection

DNA extraction was carried out from fresh leaf tissue using the modified CTAB protocol [13]. Amplification of DNA regions was performed using a standard polymerase chain reaction (PCR). We selected nuclear (ITS) and plastid (*atpB*-*rbcL*) nucleotide sequences which have exhibited their efficacy for understanding phylogenetic relationships at various taxonomic levels within the Balsaminaceae [4, 6, 12, 14]. Primers and PCR protocols for *atpB-rbcL* and ITS are followed based on the published literatures [14, 15].

### Sequence alignment and phylogenetic reconstruction

DNA sequences were initially aligned with the help of CLUSTAL X version 1.83 [16] with minor subsequent manual adjustment using the software BioEdit verion 5.0.9 [17]. To evaluate congruence between different DNA regions, we analyzed nuclear (ITS) and chloroplast (*atpB*-*rbcL*) dataset separately to see if they produce an identical topology. We observed no significant difference in topologies of ITS and *atpB-rbcL* (S1 and S2 Figs). We therefore analyzed ITS and *atpB-rbcL* dataset in combination. Phylogenetic analyses were carried out using maximum parsimony (MP) as implemented in PAUP [18]. MP analyses were conducted using heuristic searches with 1,000 replicates of random taxon addition and tree rearrangements using tree bisection-reconnection (TBR) branch swapping to obtain the parsimonious trees. Gaps were considered as missing data and all characters were treated as unordered and equal weights. The node supports for clades were assessed by bootstrap (BS_MP_) analyses, which were performed using 1,000 replicates with ten random taxon additions and heuristic options [19].

For the Bayesian inference (BI) analysis, posterior probabilities (PP) were conducted in MrBayes version 3.2.3 [20], implemented in CIPRES Science Gateway [21]. The best-fitting model of sequence evolution in BI analyses was chosen for each marker using ModelTest 3.7 [22] under the Akaike Information Criterion (AIC) in conjugation with PAUP [18]. Evolutionary models that best fitted ITS, *atpB*-*rbcL* and combined dataset were GTR + I + G (nst = 6, rates = invagamma). Two separate runs, each with four-Markov-Chain Monte Carlo (MCMC) analyses were performed, starting with a random tree, proceeding for 20000000 generations (for combined dataset) and sampling one tree every 1000 generations. Majority rule (>50%) consensus trees were reconstructed after removing the ‘burn-in phase’ samples (the first 25% of the sample trees).

### Checklist preparation

To prepare an updated checklist of Balsaminaceae, we reviewed all scientific names of Balsaminaceae, which had been recorded in Nepal. All the scientific names were checked according to Shenzen code of International Association for Plant Taxonomy [23] together with online consulted from World Flora Online, The Plant List and International Plant Names Index.

## Results

**Fig 1:**
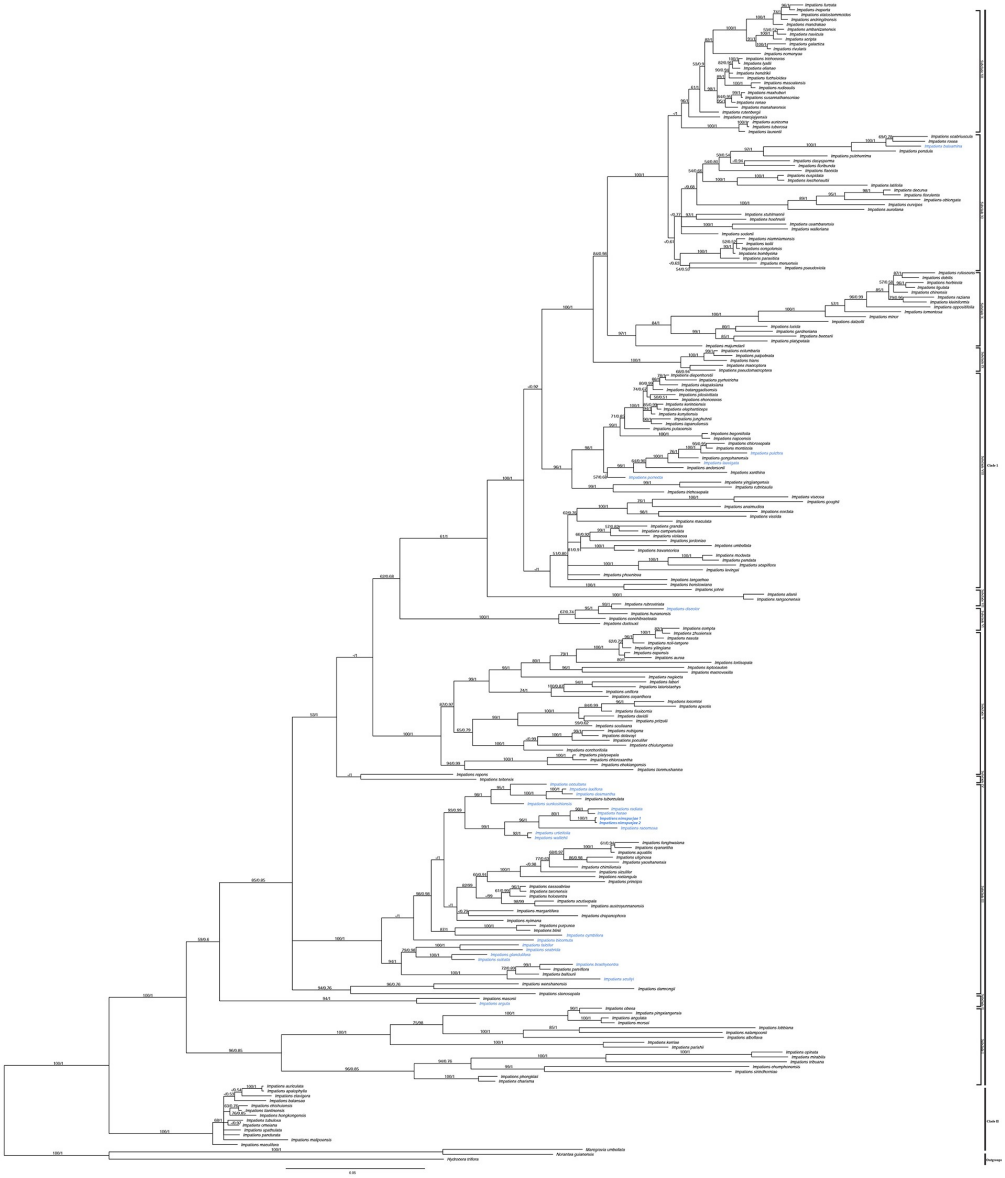
Bayesian inference tree of *Impatiens* based on the combined nuclear (ITS), and chloroplast (*atpB-rbcl*) markers. Numbers above branches indicate bootstrap percentages (BS_MP_) for MP analyses and posterior probabilities (PP) for BI analysis, respectively. Asterisk (*) indicates BS_MP_ = 100 and PP = 1.0, a dash (−) indicates support at a node < 50%. Scientific name marked in blue color indicates species occurring in Nepal.

The length of aligned matrix of ITS was 857 bp, whereas *atpB*-*rbcL* matrix consisted of 1112 bp. The combined (nuclear and chloroplast) data matrix consisted of 1969 characters of which 296 were variable and 934 were parsimony informative. The topologies from ITS, *atpB-rbcl* and combined analysis were congruent (S1 and S2 Figs and Fig 1). The BI tree based on combined dataset is shown for the discussion of phylogenetic relationships (Fig 1).

The phylogenetic tree inferred from single and combined dataset are congruent with the previous phylogenetic analysis [6, 12, 24]. *Impatiens* is divided into two major clades (i.e. Clade 1 and Clade II). Clade I is further divided into strong to weakly supported different subclades (Fig 1).

Our molecular results based on nuclear (ITS) and chloroplast (*atpB*-*rbcL*) DNA sequence data indicated that newly sampled taxon nested within Clade I (Fig 1). This species form a separate lineage within Clade I/Sublcade III (BS_MP_ = 80%, PP = 1.00), which is strongly supported as sister to *I*. *harae* and its allies (BS_MP_ = 90%, PP = 1.00).

The major morphological similarities within the clade belonging to newly sampled species and allied taxa consist of many-flowered, racemose inflorescences, lateral sepals 2, rarely 4 with inner 2 reduced, capsule linear, seed ovoid.

### Taxonomic treatment

**Fig 2:**
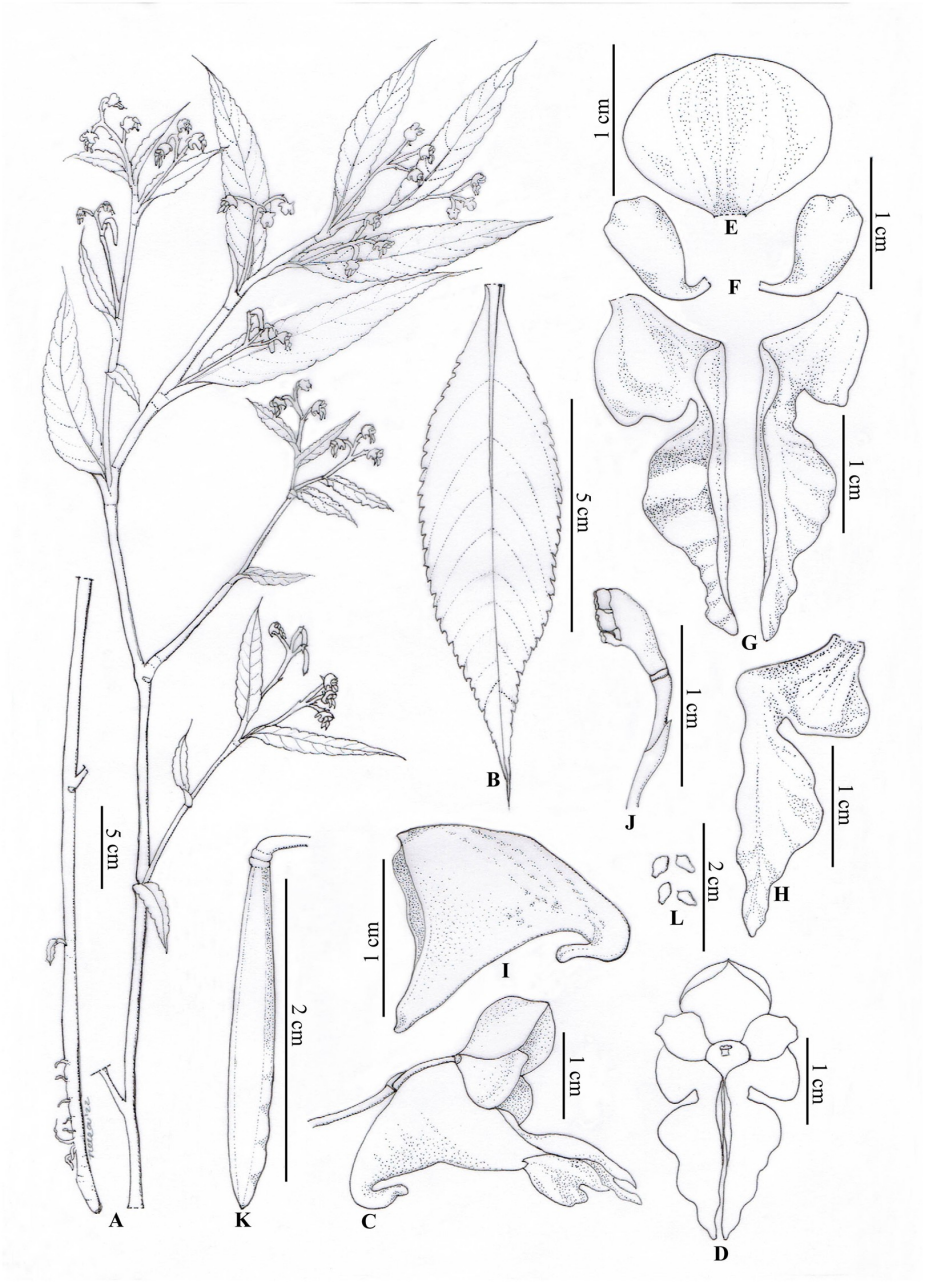
Line drawings of *Impatiens nimspurjae*: A. Flowering plant; B. Leaf; C. flower, side view; D. Flower (front view); E. Dorsal sepal; F. lateral sepals; G. Lateral united sepals (front view); H. Lateral united sepal (back view); I. Lower sepal; J. Ovary, pistil, pedicel and bract; K. Capsule; L. Seeds. Line drawings assisted by Neera Joshi Pradhan.

**Fig 3:**
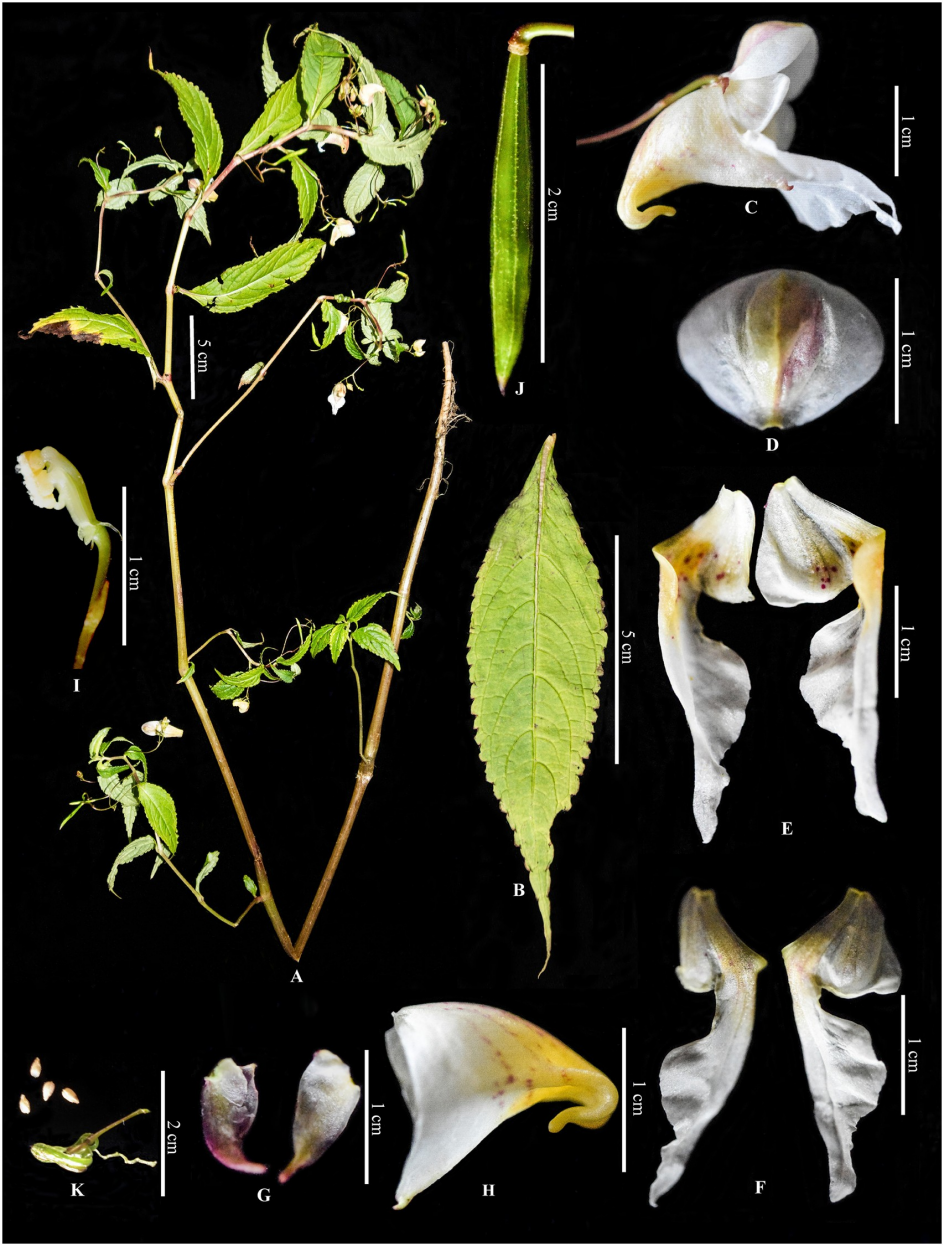
*Impatiens nimspurjae*: A. Flowering plant; B. Leaf; C. flower, side view; D. Dorsal petal (dorsal view); E. lateral united sepals (ventral view); F. lateral united sepals (dorsal view); G. Lateral sepal; H. Lower sepal; I. Ovary, pistil, pedicel and bract; J. Capsule; K. seeds. Photographs by Bhakta Bahadur Raskoti.

*Impatiens nimspurjae* Raskoti, sp. nov. [urn:lsid:ipni.org:names: 77304428–1] (Figs 2 and 3).

Type:—NEPAL. Gandaki Province: Myagdi District, above Moreni, at an elevation range of 2800–2900 m, 25 Aug. 2011, *Bhakta B*. *Raskoti 201190* (holotype, designated here: KATH); isotype *Bhakta B*. *Raskoti 201190* (TUCH).

### Diagnosis

*Impatiens nimspurjae* is closely related to *I*. *harae*, *I*. *radiata*, *I*. *wallichii* but differ from these species by having sessile oblong-lanceolate leaves, flowers less than 3 in each peduncle, base of the spur flattened, apex of dorsal petal rounded, basal lobe of lateral united petal widely ovate, not truncated.

### Description

Annual herbs, 12–99 cm tall. Stems glabrous, succulent, erect or creeper, slender, often branched above. Leaves alternate, sessile; leaf blades oblong to lanceolate, 1.5–11 × 0.5–3 cm, glabrous, setose between teeth, base cuneate and tapering, margin crenate, apex acuminate, lateral veins 5–7 pairs, whitish green. Inflorescences axillary, 2–3-flowered, peduncle 1.5–6 cm long, 1 mm diam, glabrous, pedicel 0.5–1 cm long, 1–1.5 mm diam, bracteate above base. Bracts persistent, lanceolate, 1–2 mm long. Flowers 1.5–2 cm long, white, flushed with yellowish and tinge purple on dorsal petal and lower sepal. Lateral sepals 2, falcately ovate, 2–3 × 1.5–2 mm, entire, apex mucronulate. Lower sepal bucciniform, 1–1.5 cm (excluding spur), apex with a small crest, basal portion narrowed into a hooked spur; spur stout with flat base, longitudinal canal on dorsal and ventral surface, obtuse. Dorsal petal orbicular, ca. 1 × 1 cm, apex rounded; lateral united petals ca. 1.5–2 cm long, basal lobe widely ovate, entire; apical lobe bilobulate. Stamens 5, filaments linear, anther small, apex obtuse. Ovary 5–7 mm long, erect, apex acute, glabrous. Capsule erect, linear, 1.5–2.5 cm long, 5-angled, sparsely verruculose along ribs; seeds ovoid, brownish.

### Phenology

Flowering and fruiting occur from July to October.

### Etymology

The species epithet ‘*nimspurjae’* refers to the name of Mr. Nirmal Purja (nickname Nimsdai) for his initiation on conservation through climate change champion. Mr. Purja is a world-renowned mountaineer from Nepal and has dozens of Guinness book world records in the field of mountaineering.

### Distribution and habitat

*Impatiens nimspurjae* is known from the type locality in western Nepal where it occurs in the temperate forest and forest margins at an elevation range of 2800–2900 m. It grows on moist humus rich slopes.

### Taxonomic notes

**Fig 4:**
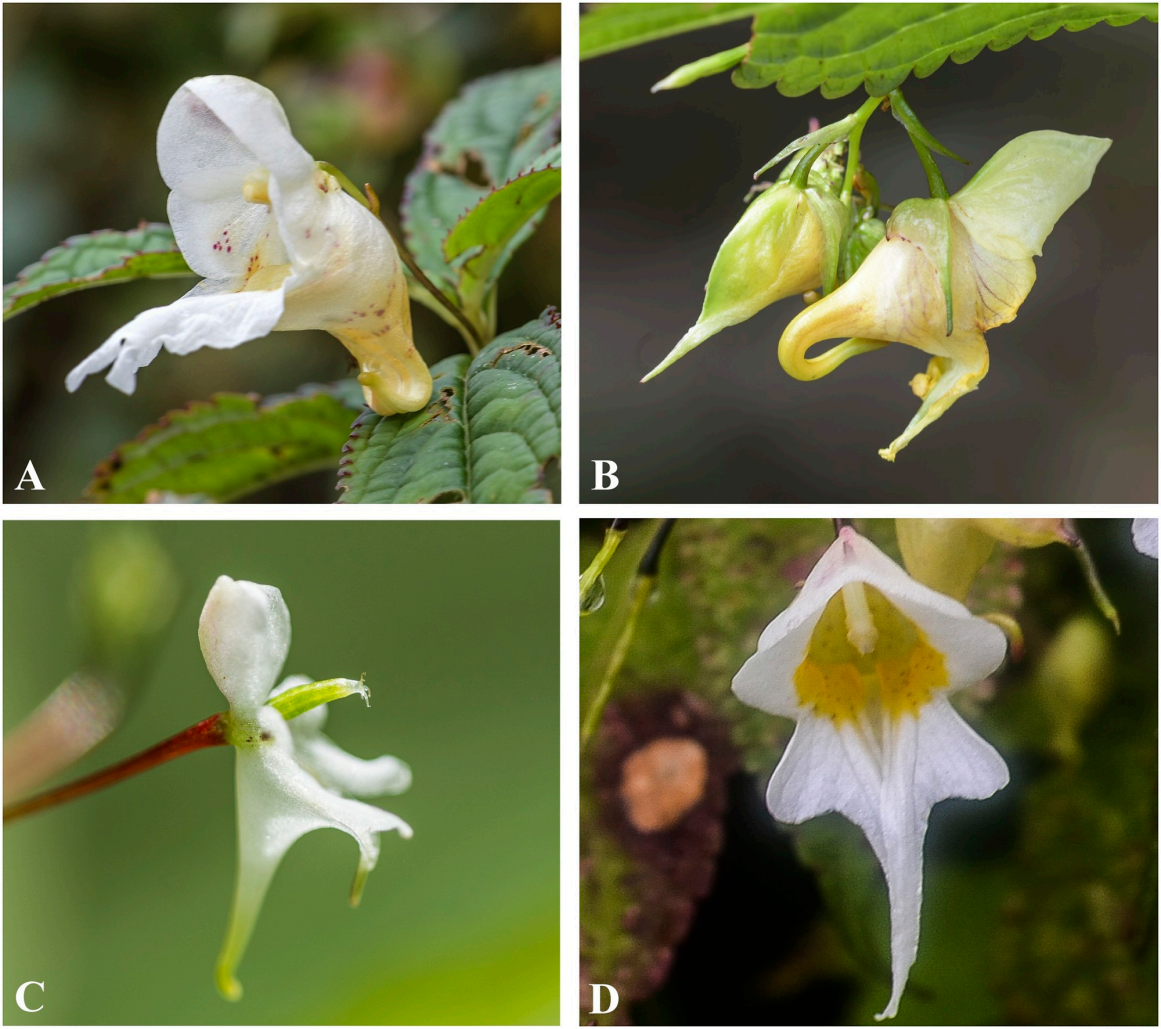
Flowers of *Impatiens nimspurjae* and its allied species: A. I. *nimspurjae*; B. *I*. *urticifolia*; C. *I*. *radiata*; D. *I*. *harae*. Photographs by Bhakta Bahadur Raskoti.

*Impatiens nimspurjae* is closely related to *I*. *radiata*, *I*. *harae*, *I*. *wallichii* and *I*. *urticifolia* having many-flowered, racemose inflorescences, lateral sepals 2, rarely 4 with inner 2 reduced, capsule linear, seed ovoid but *I*. *nimspurjae* can be distinguished by its sessile, oblong to lanceolate leaves, less than three flowers in each inflorescences, flowers flushed with yellow and tinged purple, base of spur much flat (Fig 4A). The apex of dorsal petal of *I*. *nimspurjae* is rounded (vs. retuse in *I*. *wallichii* and mucronate in *I*. *urticifolia*). Moreover, basal lobe of lateral united petal is not truncated and base of spur is much flattened. *Impatiens nimspurjae* is also similar to *I*. *harae* and *I*. *radiata* by having white flower with purple tinge but latter two have clustered leaves and radiated inflorescences.

### Conservation status

*Impatiens nimspurjae* is endemic to western Nepal and restricted in only three populations. During the fieldworks in 2008 and 2011 a total of about 100 individuals were found in an area of < 9 km^2^. It is expected that size of its population may be larger in the type locality and surrounding areas. However, the extent of occurrence and exact population size are unknown. According to these preliminary information *I*. *nimspurjae* is assessed as Data Deficient (DD) following the IUCN Red List Categories and Criteria [25]. The major threat for this species is habitat disturbance from anthropogenic activities such as deforestation, trampling, over grazing etc.

### New addition to flora of Nepal

During the different botanical surveys in different parts of Nepal, we discovered following five species of *Impatiens* as new records for flora of Nepal.

***Impatiens brachycentra*** Kar. & Kir.

NEPAL. Central Nepal: Myagdi district, at an elevation of 2400 m, *Bhakta B*. *Raskoti* 20103 (KATH).

***Impatiens cathcarthii*** Hook.f.

NEPAL. Eastern Nepal: Ilam district, at an elevation of 1300 m, *Bhakta B*. *Raskoti* 20117 (KATH).

***Impatiens gammiei*** Hook.f.

NEPAL. Eastern Nepal: Taplejung district, at an elevation of 3400 m, *Bhakta B*. *Raskoti* 20109 (KATH).

***Impatiens infundibularis*** Hook. f.

NEPAL. Eastern Nepal: Ilam district, Kolbung, at an elevation of 1400 m, *Bhakta B*. *Raskoti* 20101 (KATH).

***Impatiens sikkimensis*** Govaerts & Chakrabarty

NEPAL. Eastern Nepal: Ilam district, Kolbung, at an elevation of 1500 m, *Bhakta B*. *Raskoti* 201145 (KATH).

### Identification key to the species of *Impatiens* in Nepal

1a. Capsule ovoid, fusiform.............................................................................................................2

1b. Capsule linear, cylindrical, rarely clavate................................................................................13

2a. Inflorescence fascicled in leaf axils, peduncle very short (less than 5 mm)...................... 1) *I*. *arguta* [sect. *Fasciculatae*]

2b. Inflorescence not fascicled, peduncle longer than 5 mm (except in *I*. *tripetala*)………...….3

3a. Inflorescence subscorpioid cymes, flowers white tinge with pink............................4 [sect. *Scorpioidae*]

3b. Inflorescence not scorpoid, flowers pinkish, yellow, white...........................6 [sect. *Uniflorae*]

4a. Spur distinctly involute, peduncle short (upto 5 cm), crest of dorsal petal 3–6 mm long................................................................................................................................2) *I*. *discolor*

4b. Spur not distinctly involute, peduncle more than 5 cm long …………..…..............................5

5a. Leaves glabrous, bracts broadly ovate-orbicular, lateral petals often orbicular, crest of dorsal petal 3–6 mm long,.…..............................................................................................3) *I*. *cathcarthii*

5b. Leaves hairy, bracts ovate, lateral petals usually ovate (not orbicular), crest of dorsal petal 6–16 mm long …………………..........................................................................................4) *I*. *jurpia*

6a Capsule ovoid, seeds rounded, upper lobe of lateral united petals distinctly smaller than lower lobe (plant cultivated or naturally grows in abandoned land)……………………..5) *I*. *balsamina*

6b. Capsule fusiform, seeds ovoid or ellipsoid……………………..………….…………...……..7

7a. Peduncle short (not exceeding 1 cm long), spur curved, lower sepal saccate, spur curved...………………………………………………………………...…………….6) *I*. *tripetala*

7b. Peduncle long (more than 1 cm)...............................................................................................8

8a. Lower sepal infundibuliform with long spur.............................................................................9

8b. Lower sepals saccate, bucciniform, navicular, spur usually not very long..............................10

9a. Dorsal petal with crest, upper lobe of lateral united petal smaller than the lower lobe, spur curved, flower deeply pink………………………………...…………………..7) *I*. *infundibularis*

9b. Dorsal petal without a crest; upper lobe and lower lobe of lateral united petals nearly equal in size, flower light pink, spur filliform.................................................................................8) *I*. *exilis*

10a. Flowers reddish purple, lower sepal navicular or bucciniform………………………….….11

10b. Flower yellow or orange, lower sepal bucciniform.............................................................. 12

11a. Spur coiled, lower sepal bucciniform...................................................................9) *I*. *spirifera*

11b. Spur not coiled, lower sepal deeply navicular...................................................10) *I*. *puberula*

12a. Spur not coiled, lower sepal obliquely navicular................................................11) *I*. *porrecta*

12b. Spur coiled, peduncles more than 10 mm long; flowers yellow or orange, lower sepal bucciniform….............................................................................................................12) *I*. *pulchra*

13a. Fruits 4-lobed, ovary 4-carpellate……………….……13) *I*. *laevigata* [sect. *Semeiocardium*]

13b. Fruits 5-lobed, ovary 5-carpellate…………………………….………..14 [sect. *Racemosae*]

14a. Leaves opposite at base and middle of stem………………………...…………....………..15

14b. Leaves alternate………………….........................................................................................17

15a. Spur of lower sepal not constricted but distally curved............................14) *I*. *chungtienensis*

15b. Spur of lower sepal constricted..............................................................................................16

16a. Upper leaves verticillate and serrate, capsule clavate................................. 15) *I*. *glandulifera*

16b. Upper leaves alternate and crenate, capsule cylindrical.......................................16) *I*. *sulcata*

17a. Lower sepal directed upwards……………….......................................................................18

17b Lower sepal directed downwards..........................................................................................21

18a. Leaves ovate-lanceolate, dorsal petal reflexed at middle, lower sepal navicular, apex of lateral sepals sickle-shaped…………..……………………..………………...17) *I*. *drepanophora*

18b. Dorsal petal not reflexed at middle........................................................................................19

19a. Leaves elliptic-lanceolate, lower sepal funneliform, apex of lateral sepals acute.........18) *I*. *stenantha*

19b. Lower sepal funneliform-tubular……………………………..……………………………20

20a. Leaves linear-narrowly elliptic, apex of the lateral sepals acute……...…………19) *I*. *prainii*

20b. Leaves elliptic-lancolate, apex of lateral sepals acuminate……….………...20) *I*. *leptocarpa*

21a. Leaves usually cluster, inflorescences radiated.....................................................................22

21b. Leaves not clustered...............................................................................................................32

22a. Flowers white.........................................................................................................................23

22b. Flowers pink, yellow, purple or whitish purple.....................................................................24

23a. Apex of distal lobe of lateral united petals long tailed, flower tinge with purple.……………….…………………………………………………………………21) *I*. *harae*

23b. Flower not tinge with purple (except in *I*. *arunensis*)...........................................................27

24a. Leaves alternate and aggregated at the apical part of the stem, spur curved downwards, 17–36 mm long…………………………………………………………………...….22) *I*. *graciliflora*

24b. Spur (if present) not curved downwards but straight.............................................................25

25a. Lower sepal spurless...................................................................................23) *I*. *brachycentra*

25b. Lower sepal navicular (7–15 mm long)……………………………………........................26

26a. Lower sepals with spur, spur straight and tapering gradually…………………..24) *I*. *radiata*

26b. Lower sepals spurless, other characters similar as in *I*. *radiata*………………25) *I*. *gammiei*

27a. Flower redish purple, spur short..………………………………..….…………………….28

27b. Flowers yellow, spur long……………………………………...……………..……..........30

28a. Flower white with pink dots………………………………….……….…….26) *I*. *arunensis*

28b. Flower purple…………………………………………………………………………….…29

29a. Flowers purplish red with brownish dots………….…………………………27) *I*. *pradhanii*

29b. Flowers reddish purple with dark reddish purple dots.................................... 28) *I*. *bicornuta*

30a. Leaves opposite decussate, spur filiform………………………….……..… 29) *I*. *leptoceras*

30b. Leaves alternate, spur not filiform, curved downward……….………………………….…31

31a. Spur 5–8 mm long, apex of the lateral sepal aristate…….…………..……….30) *I*. *racemosa*

31b. Spur longer than in I. racemosa (up to 14 mm long)…………………….31) *I*. *recticalcarata*

32a. Leaves sessile, flower white with flushed yellow, spur flat at base...............32) *I*. *nimspurjae*

32b. Leaves petiolate, flower yellow, spur not flat at base…………………….…….…………33

33a. Spur incurved forward........................................................................................................ 34

33b. Spur not incurved forward……………………………………………….….………….....35

34a. Stem with several branches, flowers yellow with reddish veins, spur incurved forward.....................................................................................................................33) *I*. *urticifolia*

34b. Stem unbranched, spur S-shaped; flowers pale yellow with reddish dots……34) *I*. *wallichii*

35a. Lower sepal carinate, spur absent.........................................................................................36

35b. Lower sepal not carrinate, spur present................................................................................39

36a. Leaves nearly glabrous, flowers white, center shaded purple..............................35) *I*. *serrata*

36b. Leaves usually pubescent......................................................................................................37

37a. Flowers 15–18 mm long, pale yellow…………………………………...…....36) *I*. *kharensis*

37b. Flower white or yellow with purple coloration……………………...………......................38

38a. Flowers white, shaded reddish purple in center…………………..................37) *I*. *occulatans*

38b. Flowers yellow, with dark purple markings, leaves pubescent........................38) *I*. *williamsii*

39a. Flower pale yellow or whitish yellow....................................................................................40

39b. Flower pinkish or yellow…………………………………………………………...……....46

40a. Upper lobe of the lateral united petal notched………………...……………39) *I*. *sikkimensis*

40b. Upper lobe of the lateral united petal not notched……………………………………...….41

41a. Lower lobe of lateral united petals bilobed, upper lobule falcate………..….... 40) *I*. *falcifera*

41b. Lower lobe of the lateral united petal not lobed...................................................................42

42a. Lower sepal bucciniform…………………………………….………….............41) *I*. *tricoris*

42b. Lower sepal navicular, funneliform or saccate....................................................................43

43a. Flower yellowish………………………………………………………….……...……..…44

43b. Flower yellowish white……………………………………………………..…….……….45

44a. Lower sepal navicular or infundibuliform, tapering into upwardly or downwardly curved spur............................................................................................................................42) *I*. *scabrida*

44b. Lower sepal saccate........................................................................................43) *I*. *bajurensis*

45a. Apex of upper lobe of lateral united petals obtuse..............................................44) *I*. *bicolor*

45b. Apex of upper lobe of lateral united petals falcate.………………..…..….45) *I*. *edgeworthii*

46a. Spur straight, horizontal, short, flower purple......................................................................47

46b. Spur usually curved, not horizontal (except in *I*. *insignis*) flowers pink or yellow..……….48

47a. Bract ovate, flower purple to white…………………...………….…....….….46) *I*. *cymbifera*

47b. Bract linear-lanceolate, flower dark pink………………………..………47) *I*. *gorepaniensis*

48a. Spur long and horizontal.....................................................................................48) *I*. *insignis*

48b. Spur short and not horizontal...............................................................................................49

49a. Leaves glucose, pedicels bracteate at middle; lower sepal bucciniform……………….49) *I*. *glauca*

49b. Leaves not glucose…………………………………………………..……………………..50

50a. Flower yellow or purple, spur abruptly constricted……………….……………………….51

50b. Flower pink or yellow, spur not abruptly constricted……….…………..………..….…….53

51a. Spur incurved forward, abruptly constricted, flowers pale yellow with reddish dots, lateral sepals 2…………………………………………………………..….………….…...50) *I*. *hobsonii*

52b. Flowers purple, spur curved downward, lateral sepals 4....................................51) *I*. *gamblei*

53a. Lateral united petals with appendage elongating into tubular lower sepal….……..52) *I*. *scullyi*

53b. Lateral united petal without appendage elongating into tubular lower sepal………..…….54

54a. Petals hook like, flowers pale reddish purple………………………...……53) *I*. *uncipetala*

54b. Petals not hook like, flowers pink, yellow………………………………………………..55

55a. Leaves serrated, lower sepal shallowly navicular, tapering into downwardly curved spur……………………………………………………………………………..…54) *I*. *serritifolia*

55b. Leaves not serrated, dorsal sepal orbicular, lower sepal navicular………….........……..…56

56a. Flower yellow, lower sepals with incurved spur……………………….……55) *I*. *desmantha*

56b. Flower pink…………………………………………………………………….………...…57

57a. Basal lobe of lateral united petal narrowly triangular, lower sepal with abruptly constricted erect spur and curved distally…….………………………………………………....56) *I*. *laxiflora*

57b. Basal lobe of lateral united petal orbicular, spur straight, abruptly constricted……….…57) *I*. *sunkoshiensis*

### Updated checklist of Balsaminaceae in Nepal

***Impatiens arguta*** Hook. f. & Thomson, J. Linn. S. B. 4: 137 (1860).

Type: *Hooker J*.*D*. *s*.*n*. (lecto, designated in [26] K K000694618!), Sikkim Himalaya, Darjeeling, 7500 ft.; *Hooker J*.*D*. *s*.*n*. (syn, K K000694619!), Darjeeling.

*Impatiens gagei* Hook. f. Hooker’s Icon. Pl. 30: t. 2951 (1911).

Type: *Burkill 27744* (lecto, designated in [26], K K000694617!), Eastern Himalaya, Darjeeling, Tonglo slopes, below Simana, at an elevation range of 6000–8000 ft.

Habitat: Temperate forest at an elevation range of 2100–2900 m.

Distribution: Eastern Nepal, China, India and Myanmar.

Specimen examined: Solukhumbu district, Naamkhali, 2240 m, 21 Jul. 1995, *Miyamoto F*. *et al*. *9592063* (KATH); Sankhuwasabha district, Arun Valley, Maghang Khola, 2550 m, 01 Jul. 1956, *Stainton J*.*D*.*A*. *817* (TI); Nigale, 5500 ft., 11 Sept. 1964, *Banerjee*, *Shrestha & Upadhya 2633* (US); Phaplos, 8000–9000 ft., 1930, *Lall Dhwoj 79* (E); Tinjure Danda, 2400 m, 06 Sept. 1967, *Williams & Stainton 8402* (TI); Dhankuta district, Bilbatay Bhanjang, 2200 m, 25 Oct. 1963, *Hara H*. *et al*. (TI); Dhankuta district, Chitre, 2400 m, 07 Jul. 1972, *Kanai H*. *et al*. *721162* (TI); Dhankuta district, Shidua-Tute, 13 Jul. 1991, *Ohba H*. *et al*. *9120029* (TI); Panchthar district, Prangbung-Bhanduke, 2300 m, 25 Jun. 1992, *Noshiro S*. *et al*. *9241070* (TI); Ramechhap district, Bhandar-Sete, 1880 m, 29 Jul. 1997, *Wakabayashi M*. *et al*. *9710032* (TI); Rasuwa district, Syabru-Dhunche, 2300 m, 25 Jul. 1992, *Takayama H*. *et al*. *9241088* (TI); Solukhumbu district, Bandar-Namkhli, 20 Jul. 1995, *Miyamoto F*. *et al*. *9584017* (TI); Solukhumbu district, Fera-Nunthala, 2780 m, 26 Jul. 1995, *Miyamoto F*. *et al*. *9596393* (TI).

**Fig 5:**
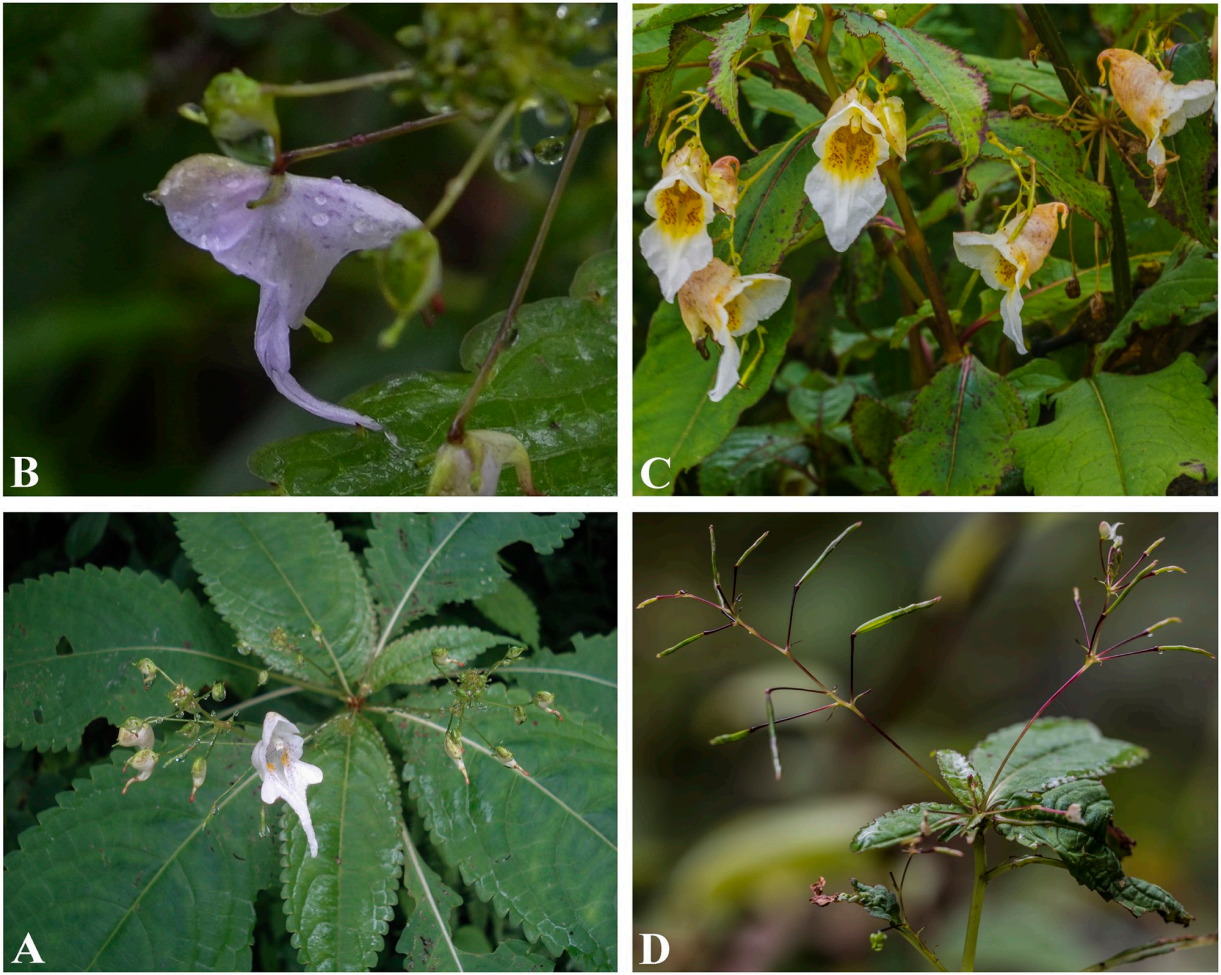
A, B. *Impatiens arunensis*; C. *Impatiens wallichii*, D. *Impatiens gammiei*. Photographs by Bhakta Bahadur Raskoti.

***Impatiens arunensis*** Grey-Wilson, Kew Bull. 44 (1): 65 (1989). Fig 5A and 5B.

Type: *Grey-Wilson et al*. *4376* (holo, K K000694616!), Nepal, Arun Khola, Upper Kashwa Khola, above Hedangha, elevation 2800 m, 08 Sept. 1981.

Habitat: Temperate forest at an elevation about 2800 m.

Distribution: Eastern Nepal (endemic).

Specimen examined: Arun khola, upper kashwa khola, above Hedangha, 2800 m, 08 Sept. 1981, *Bhandary & Taylor 4376* (K); Sankhuwasabha district, Arun khola, banking, Sekidim, 2600 m, 15 Aug. 1981, *Grey-Wilson*, *Christopher and Henderson 4376* (E).

Note: *Impatiens arunensis* is morphologically similar to *Impatiens harae*; a comprehensive study is necessary for the taxonomic identity of *Impatiens arunensis*.

***Impatiens bajurensis*** S. Akiyama & H. Ohba, Journ. Jap. Bot. 68: 157 (1993).

Type: *Suzuki M*. *et al*. *9171054* (holo, TI!; iso KATH!), Far West Nepal, Bajura district, Kaudegaon-Babali, 1520 m, 29 Aug. 1991.

Habitat: Subtropical forest at an elevation of 1520 m.

Distribution: Western Nepal (endemic).

Specimen examined: Doti district, Siligarhi, 4500 ft., 01 Apr. 1967, *Ecker-Racz N*. *38* (US); Between Munigaon and Chutta, S.E. of Jumla, 9500 ft., 24 Jul. 1952, *Polunin*, *Sykes & Williams 4901* (E); Mugu district, Kamali Valley, near Lumsa, 7000 ft., 14 Aug. 1952, *Polunin*, *Sykes & Williams 5186* (E); Mugu district, Ratuligaon, *Bis Ram 350* (NY); Mugu district, Samla, 7000 ft., 04 Oct. 1952, *Polunin*, *Sykes & Williams 5560* (E).

***Impatiens balsamina*** L., Sp. Pl. 938 (1753).

Type: *Anonymous s*.*n*. (lecto, designated in [27], LINN-HL1053.5).

*Balsamina cornuta* (L.) DC., Prodr. 1: 686 (1824).

Type: *Paul Hermann 3*:*9*, *n*. *316* (lecto, designated in [28], BM BM000621820), Sri Lanka.

*Impatiens coccinea* Sims, Bot. Mag. 31: t. 1256 (1810).

Type: *Sims (1810) t*.*1256* (lecto, designated in [26], illustration of specimen cultivated in Sloane street by Mr. Salisbury, seeds from Mr. Roxburgh, east India.

*Impatiens balsamina* var. *longifolia* Wight & Arn., Prodr. 1: 136 (1834).

Type: *Wall*. *Numer*. *List*. *n*. *4734* (K K001039792!).

*Impatiens arcuata* Wight & Arn., Prodr. 1: 136 (1834).

Type: *Wall*. *Numer*. *List*. *n*. *4735* (K K001039793!), India.

Habitat: Sub-tropical forest at an elevation range of 1200–1900 m.

Distribution: Widely cultivated in eastern, central and western Nepal, its native habitat is S.E. Asia.

Specimen examined: Darchula district, Khalenga, 840 m, 26 Aug. 1982, *Amatya M*.*M*. *& Regmi P*.*M*. *W479/82* (KATH); Chyanthapu-Khebang, 5000 ft., 13 Jun. 1969, *Shrestha T*.*B*. *15617* (KATH); Ranipauwa (north of Beni), 3500 ft., 31 Aug. 1954, *Stainton*, *Sykes and Williams 7619* (E!).

***Impatiens bicolor*** Royle, Ill. B. Him. 151, t. 28 (1834).

Type: *s*.*d*., *J*.*F*. *Royle s*.*n*. (lecto, designated in [29], K K000694726!), India, Northwest India.

*Impatiens amphorata* Edgew, Trans. Linn. Soc. London 20(1): 39 (1846).

Type: *Edgeworth M*.*P*. *332* (lecto, designated in [29], K K000694728!), India, Himachal Pradesh, Simla, 7000–8000 ft., 1844.

*Impatiens duthiei* Hook. f., Rec. Bot. Surv. India 4: 11 (1905).

Type: *Duthie’s collector (Inayat) s*.*n*. (lecto, designated in [29], upper specimen of K K000694732!; isolecto DD, lower specimen of K K000694732!), India, Western Himalaya, Uttarakhand, Kumaon, 1900.

*Impatiens edgeworthii* var. *toppinii* Hook. f. ex Toppin, Bull. Misc. Inform. Kew 1920: 348 (1920).

Type: *Toppin S*.*M*. *605* (lecto, designated in [29], larger left hand side specimen of K K000694738!; isolecto, smaller right hand side specimen of K K000694738!), Pakistan, Chitral, Ziarat, 7000–8000 ft., Aug. 1908.

*Impatiens pallens* Edgew., Trans. Linn. Soc. London 20(1): 39 (1846).

Type: *Edgeworth M*.*P*. *334* (lecto, designated in [29], left hand side specimen of K K000694729!; isolecto right hand side specimen of K K000694729!), India, Uttarakhand, Garhwal, 4000–8000 ft., 1844.

*Impatiens pseudobicolor* Grey-Wilson, Fl. Iranica 143: 9 (1979).

Type: *Burtt B*.*L*. *& Kazmi M*.*A*. *1253* (holo, E E00841614!), Pakistan, Hazara, Hill territory, 7500 ft., 18 Sept. 1958.

*Impatiens umbrosa* Edgew., Trans. Linn. Soc. London 20(1): 39 (1846).

Type: *Edgeworth M*.*P*. *334* (lecto, designated in [29], larger left hand side specimen of K K000694727), India, Himachal Pradesh, Kangra, 3000–8000 ft., 1844.

*Impatiens nepalensis* Hook. f., Rec. Bot. Surv. India 4: 14, 20. 1905 & Hooker’s Icon. Pl. 30: t. 2961 (1911).

Type: *Wallich cat*. *no*. *4729* (designated in [29], K K001039776), Nepal, 1821.

Habitat: Lower temperate forest at an elevation range of 2100–2400 m.

Distribution: Western Nepal, India and Pakistan.

Specimen examined: Dhaulakot, 7500 ft., 18 Aug. 1952, *Polunin*, *Sykes and Williams 498* (E).

**Fig 6:**
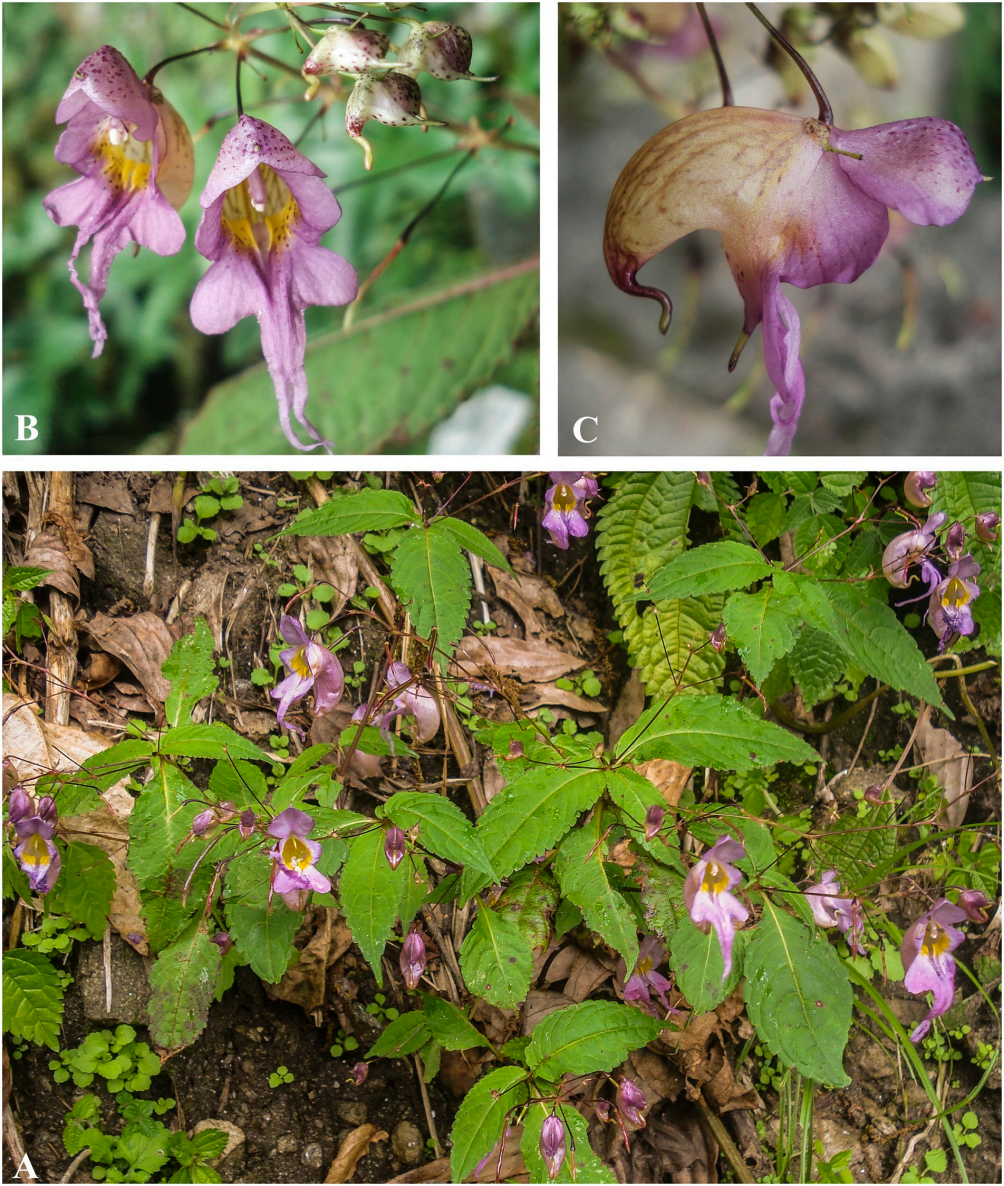
*Impatiens bicornuta* A. Flowering plant; B. Front view of flower; C. Flower (side view). Photographs by Bhakta Bahadur Raskoti.

***Impatiens bicornuta*** Wall., Roxb., Fl. Ind. 2: 460 (1824). Fig 6.

Type: *Wallich 4765* (lecto, designated in [30], K, two specimens K001039849! and K001039850!), Nepal; *Wallich cat*. *no*. *4765* (lecto, designated in [29], K K001039849!), Nepal, Chandaghery, Jun. 1821.

*Impatiens bicornuta* var. *micrantha* H. Hara, Fl. E. Himalaya 3^rd^ Rep. 78 (1975).

Type: *Kannai H*., *Hara H*. *& Ohba H*. *723214* (lecto, designated in [29], TI TI00013971!), Nepal, Rasuwa district, Dunche-Trisuli Khola-Singum Gompa, 22 Aug. 1972.

Habitat: Subtropical to temperate forest at an elevation range of 1600–3000 m.

Distribution: Eastern, Central, Western Nepal, China and India.

Specimen examined: Ramechhap district, Sivalaya, 1800 m, 17 Aug. 1995, *Ohaba H*. *et al*. *61345* (KATH); Manang district, Chame, 12 Aug. 1983, *Manandhar N*.*P*. 977 (KATH); Lalitpur district, Phulchoki, 2200–2700 m, 09 Aug. 1969, *Kani H*. *673398* (KATH); Thade, 2140 m, 18 Jul. 1971, *Shakya P*.*R*. *& Adhikari 597* (TI).

***Impatiens brachycentra*** Kar. & Kir., Bull. Soc. Imp. Naturalistes Moscou. 15: 179 (1842).

Type: *Kar*. & *Kir*. *13339* (holo, LE), Dzungarian Ala-Tau, Baskan River.

Habitat: Temperate forest at an elevation of 2400 m.

Distribution: Western Nepal, Afghanistan, China, India, Kazakhstan and Kyrgyzstan.

Specimen examined: Mygdi district, on the way to Jaljala, 2400 m, *Bhakta B*. *Raskoti 201395* (KATH).

**Fig 7:**
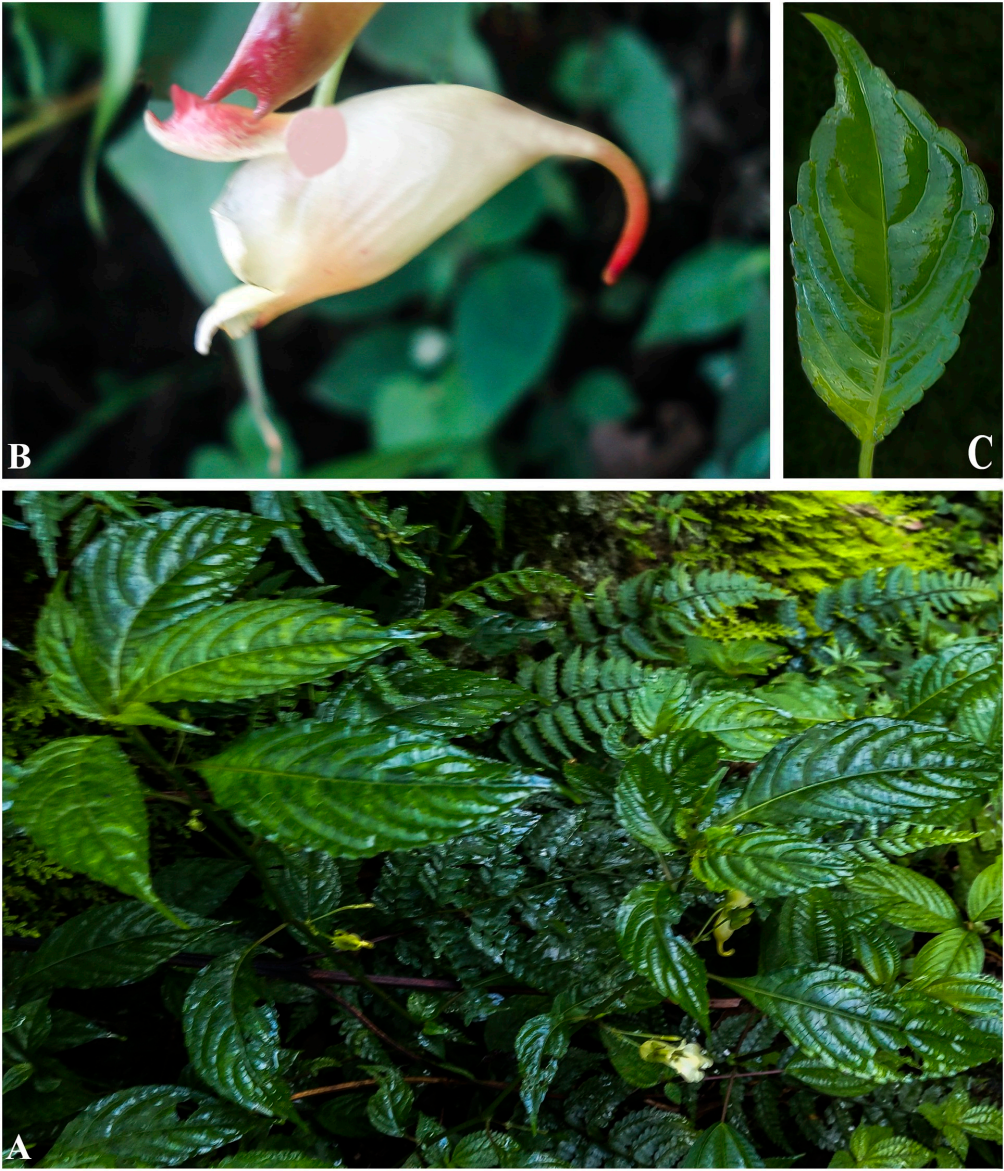
*Impatiens cathcarthii* A. Flowering plant; B. Side view of flower; C. Leaf. Photographs by Bhakta Bahadur Raskoti.

***Impatiens cathcarthii*** Hook.f. Fl. Brit. India 1: 473 (1875). Fig 7.

Type: *Hooker J*.*D*. *s*.*n*. (lecto, designated in [39], K K000694715!), Sikkim, Kursing.

Habitat: Subtropical forest and forest margins at an elevation of 1300 m.

Distribution: Eastern Nepal and India

Specimen examined: Ilam district, Lasune, 1300 m, 05 Sept. 2011, *Bhakta B*. *Raskoti 201189* (KATH).

**Fig 8:**
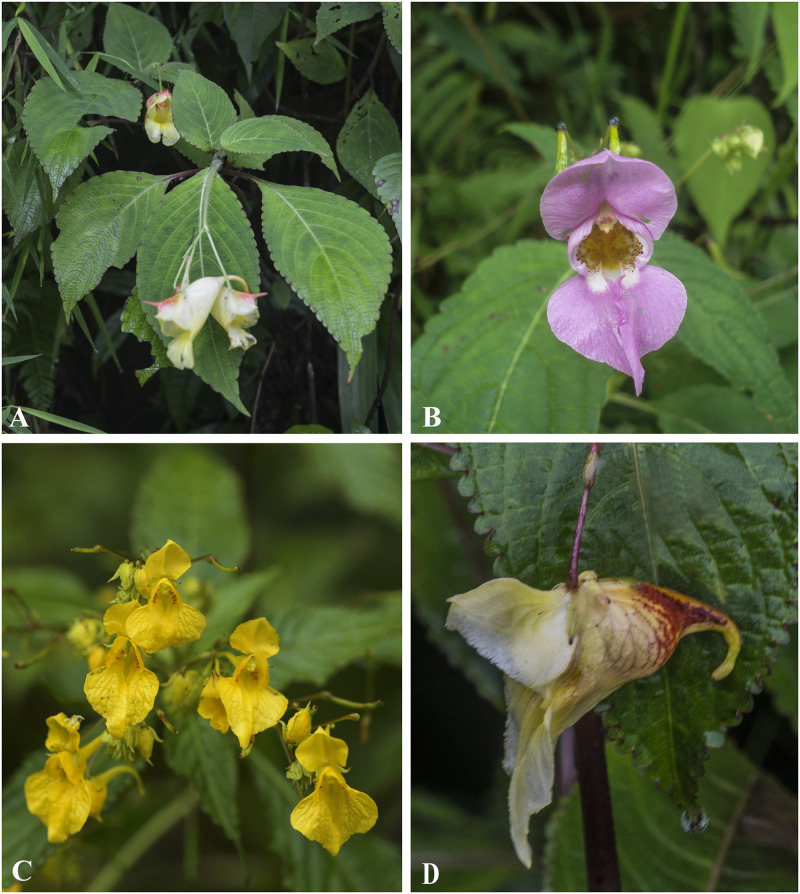
A. *Impatiens jurpia*; B. *Impatiens chungtienenis*; C. *Impatiens desmantha*; D. *Impatiens hobsonii*. Photographs by Bhakta Bahadur Raskoti.

***Impatiens chungtienensis*** Y.L. Chen, Acta Phytotax. Sin. 16(2): 44 (1978). Fig 8B.

Type: *Feng K*.*M*. *2025* (holo PE!; iso A and KUN), China, NW Yunnan, SE of Chungtien, 15 Aug. 1939.

Habitat: Temperate forest at an elevation range of 2400–2800 m.

Distribution: Western Nepal, China and India.

Specimen examined: Kuntisau, 2800 m, 29 Aug. 1980, *Rajbhandari K*.*R*. *& Malla K*.*J*. *5725* (KATH); Mugu district, below Mugu khola, 11500 ft., 24 Aug. 1952, *Polunin*, *Sykes & Williams 2590* (E); Mugu district, Mugu khola, 11500 ft., 24 Aug. 1952, *Polunin*, *Sykes & Williams 3015* (E)

**Fig 9:**
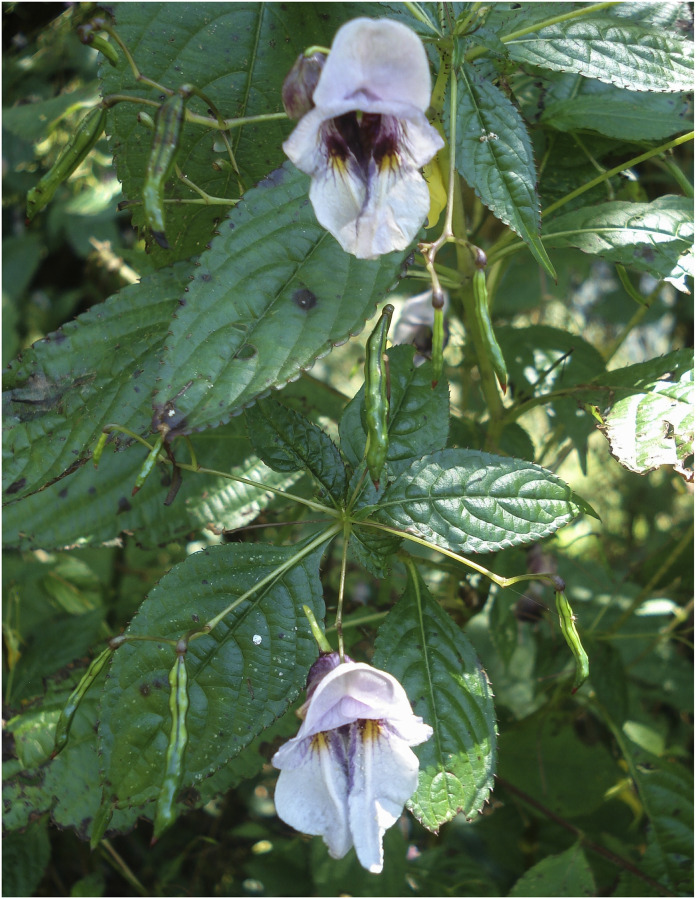
*Impatiens cymbifera* (flowering plant). Photograph by Bhakta Bahadur Raskoti.

***Impatiens cymbifera*** Hook. f., Fl. Br. Ind. 1: 474 (1875); Rec. B. Surv. Ind. 4: 14 & 20 (1905). Fig 9.

Type: *Joseph Hooker* (holo, K K000694693!), Sikkim, Lachoong (Lachung) Valley.

Habitat: Temperate forest at an elevation range of 2000–2900 m.

Distribution: Estern, Central Nepal, China, India and Myanmar.

Specimen examined: Dolakha district, Tingoang-Khosuri Kharbe, 12 Sept. 1970, *Kani H*., *Chuma C*.*H*. *& Nagano J*. *s*.*n* (KATH); Solukhumbu district, Utishey khola Tashi gaon, 2100 m, 06 Oct. 2017, *Sherpa L*.*D*. *106–1* (KATH); Panchthar district, Memeng, 2050 m, 13 Oct. 1981, *Grey-Wilson 4697* (E), Rasuwa district, above Dhunche, 2065 m, 03 Jun. 1969, *Hara H*. *et al*. *69896* (TI).

***Impatiens desmantha*** Hook., Nouv. Arch. Mus. Hist. Nat., sér. 4. 10: 248 (1908). Fig 8C.

Type: *Delavay J*.*M*. *s*.*n*. (lecto, designated in [31], P P00780638!), China, Yunnan, San-Tcha-ho, 4 Sept. 1889.

Habitat: Temperate forest and forest margins.

Distribution: Eastern and Central Nepal, China, India

**Fig 10:**
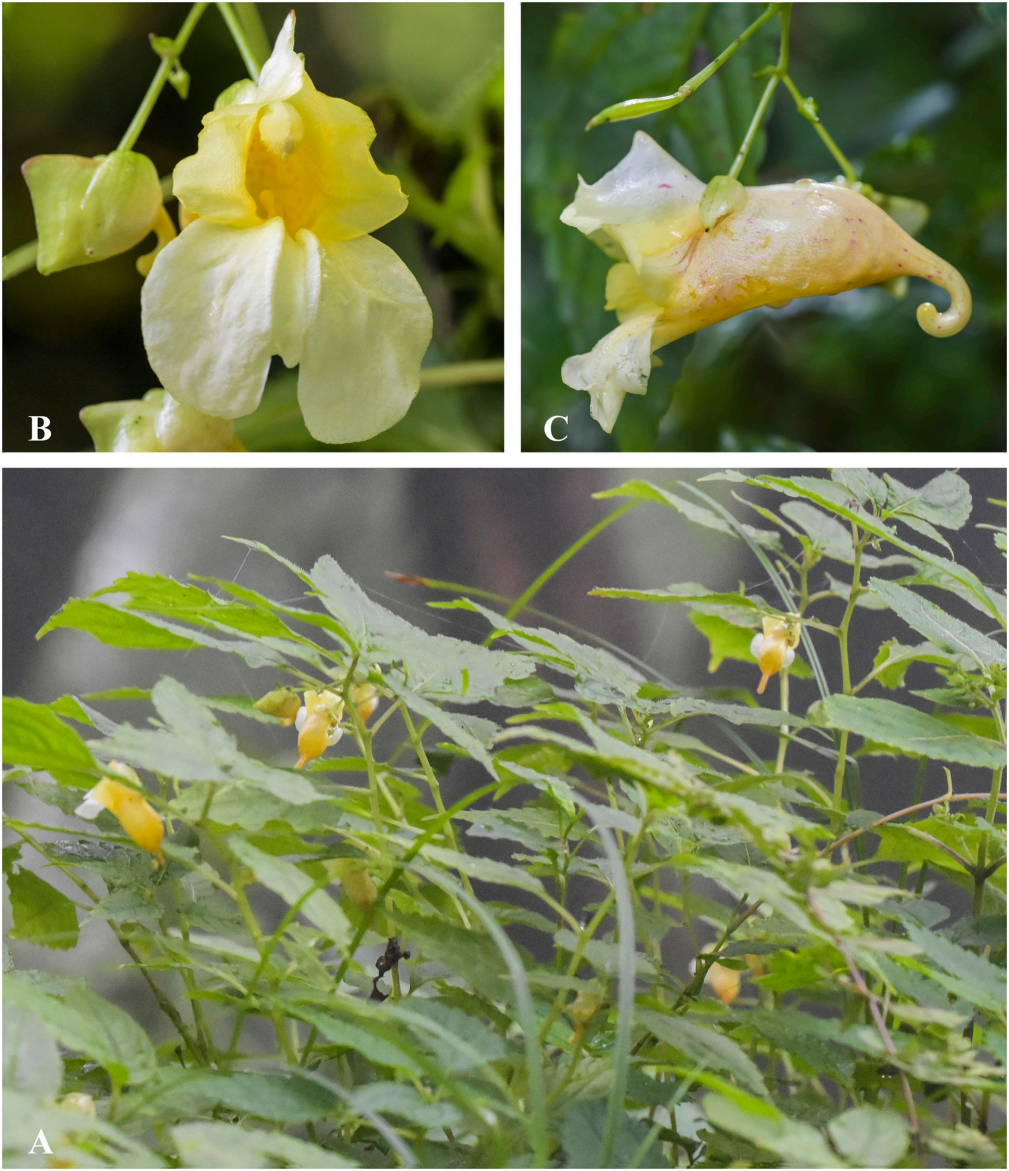
*Impatiens discolor* A. Flowering plant; B. Flower (front view); C. Flower (side view). Photographs by Bhakta Bahadur Raskoti.

***Impatiens discolor*** DC., Prodr. 1: 687 (1824). Fig 10.

Type: *Wallich s*.*n*. (holo, G G00218031), Nepal (Napaulia).

*Impatiens kathmanduensis* Grey-Wilson, Kew Bull. 44 (1): 119 (1989).

Type: *Schilling 1071* (holo, K!), Nepal, Phulchoke (Phulchoki), 8700 ft.

Habitat: Lower temperate forest at an elevation range of 1900–2200 m.

Distribution: Eastern and Central Nepal and India.

Specimen examined: Kathmandu district, Bagdwar, 7700 ft., 02 Aug. 1966, *Malla S*.*B*. *4959* (KATH); Lalitpur district, Phulchoki, 2518 m, 22 Aug. 2018, *Bhatta G*.*D*. *& Pandey T*.*R*. *7556* (KATH); Botebus-Fururu, 1800–2050 m, *Ohba et al*. *773305* (TI); Between Dobato and Pativanjyang, 2400 m, *Grey-Wilson & Phillips 214* (K); Gangia La-Palchock Danda, 7000 ft., *Polunin 1950* (BM); Gosainkund, Dunche Gyang-Thodang Danda, 2450 m, *Malla & Kanai 674619* (TI); Lalitpur, Phulchoki, 7250 ft., *Schilling 641* (TI); Lalitpur, Phulchoki, 2300–2700 m, *Ohashi et al*. *771446*, (TI); Kathmandu district, Sheopuri, *Chuma 7009224* (TI); Mangalbare, *Hara et al*. *6300497* (TI); Manglabare, 2650 m, *Dobremez 1553* (BM); Milke Danda, 9000 ft., *Beer 25740* (BM); Sati Khola, Peramchaw, 3500 ft., *Stainton et al*. *6495* (BM); Sankhuwasabha district, Tinjure Danda, 7500 ft., *Williams & Stainton 8379* (BM); Kaski, Banthati, 2180 m, *Mikage et al*. *9485565* (TI); Sankhuwasabha district, Arun Valley, between Bhotebas and Chichila, 1930 m, *Long et al*. *91* (E); Solukhumbu district, Namkhli-Gnaula, 2720 m, *Miyamoto F*. *et al*. *9584026* (TI).

***Impatiens drepanophora*** Hook. f., Rec. B. Surv. Ind. 4: 17 & 22 (1905).

Type: *Hooker* & *Thomson 56* (lecto, designated in [32], K K000694682!; isolecto K K000694683!, K K000694684!, K K000694685!, K K000694686!, K K000694687!, L L0388881), India, Khasia Hills, 06 Jul. 1850, 6000 ft.

Habitat: Temperate forest at an elevation of 2000–2200 m.

Distribution: Nepal, China, India and Myanmar.

***Impatiens edgeworthii*** Hook. f., Fl. Brit. Ind. 1: 476 (1875); in Rec. B. Surv. Ind. 4: 5 & 9 (1904).

Type: *Edgeworth 1067* (holo, K!), India, Kundau and Beas Valley, shady and open places, 1800–3000 m.

Habitat: Temperate forest at an elevation range of 2000–3000 m.

Distribution: Central Nepal, India and Pakistan.

Specimen examined: Kaski district, Panchase forest, 2340 m, 09 Sept. 2014, *Bhandari P*. *& Budhamagar S*. *P832* (KATH).

**Fig 11:**
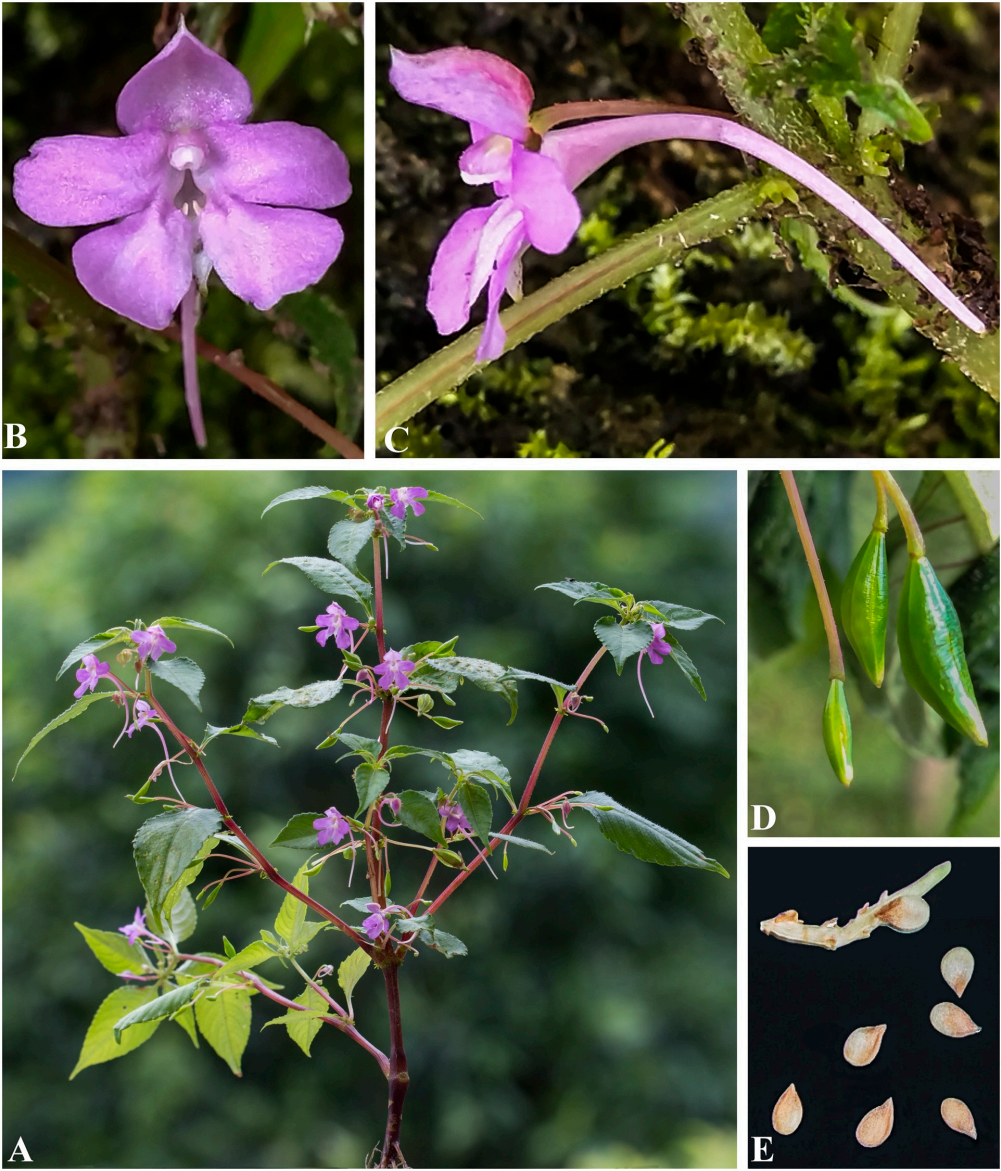
*Impatiens exilis* A. Flowering plant; B. Front view of flower; C. Flower (side view); D, Capsule; E, Seed. Photographs by Bhakta Bahadur Raskoti.

***Impatiens exilis*** Hook. f., Rec. B. Surv. Ind. 4: 13 & 19 (1905). Fig 11.

Type: *Clarke C*.*B*. *869b* (lecto, designated in [29], K K000694677!), India, West Bengal, Darjeeling, Pomong, 3000 ft., 23 Aug. 1869.

Habitat: Tropical to subtropical forest at an elevation range of 600–1200 m.

Distribution: Eastern Nepal, Bhutan and India.

Specimen examined: Sunsari district, Dharan, 600 m, 03 Sept. 1967, *Williams & Stainton 8338* (TI); Pohlara, 1050 m, 07 Spet. 1954, *Stainton*, *Sykes & Williams 7135* (E); Sanguri Bhanjang–Dhara, 16 Oct. 1963, *Hara H*. *et al*. *6300493* (TI); Sankhuwasawa district, Baidep-Num, 13 Aug. 1991, *Ohba H*. *et al*. *9120411* (TI); Pahakhola–Baidep, 12 Aug. 1991, *Ohba H*. *et al*. *9120406* (TI); Sunsari district, Dhara Pani, 1000 m, 10 Jul. 1972, *Kanai H*. *et al*. *721226* (TI); Dhara Pani-Dharan, 10 Jul. 1972, *Kanai H*. *et al*. *721226* (TI).

**Fig 12:**
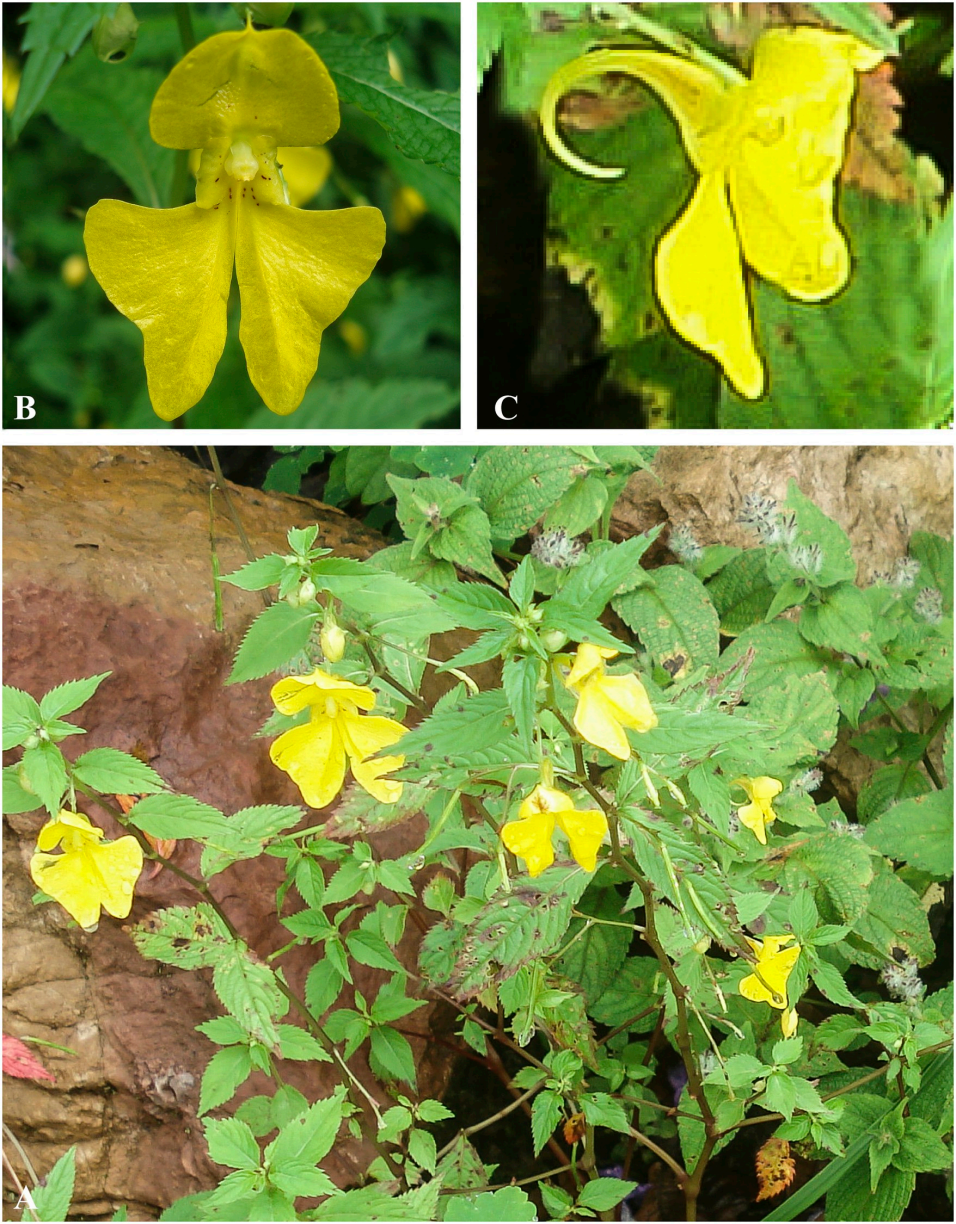
*Impatiens falcifera* A. Flowering plant; B. Flower (front view); C. Flower (side view). Photographs by Bhakta Bahadur Raskoti.

***Impatiens falcifera*** Hook. f., B. Mag. 129: t. 7923 (1903); Rec. B. Surv. Ind. 4: 18 & 23 (1905). Annotation: as "falcifer" Fig 12.

Type: *Hooker J*.*D*. *s*.*n*. (lecto, designated in [33], K K000694739!), India, Sikkim, 8000–1000 ft.

Habitat: Temperate forest at an elevation range of 2500–3400 m.

Distribution: Eastern, Central Nepal, China and India.

Specimen examined: Topke to Mauwakhola, 9500 ft., 03 Aug. 1971, *Shrestha T*.*B*. *& Joshi D*.*P*. *488* (KATH); Solukhumbu district, Kuri-Kharikhola, 3000 m, 29 Aug. 1997, *Rajbhandari K*.*R*. *9740630* (KATH); Sankhuwaabha district, Arun Valley, Chyamtang, 9000 ft., 21 Sept. 1956, *Stainton J*.*D*.*A*. *1745* (E); Maney Dara, 13000–14000 ft., *Lall Dhwoj 505* (E); Panghu Danda-Mere Danda, 23 Aug. 1969, *Kanai H*. *& Malla S*.*B*. *674694* (TI); Lalitpur district, Phulchoki, 2650 m, 15 Jul. 1972, *Hara H*. *et al*. *723210* (TI); Riala, 5000 ft., 02 Sept. 1952, *Polunin*, *Sykes & Williams 1317* (E); Gorkha district, Lungdang Gompa, 3170 m, 29 Jul. 1994, *Suzuki M*. *et al*. *9460512* (TI).

***Impatiens gamblei*** Hook. f., Rec. B. Surv. Ind. 4: 15 & 20 (1905).

Type: *Gamble J*.*S*. *8423* (lecto, designated in [11], K K000694669!), Sikkim, Darjeeling, Sandukpho, 11500 ft. Sept. 1880.

Habitat: Temperate forest at an elevation of 2800 m.

Distribution: Eastern Nepal and India.

Specimen examined: Pachther district, Memeng dabale deurali, 2640 m, 29 Sept. 2007, *Shrestha K*.*K*., *Kunwar R*.*M*., *Humagain K*., *Dhamala M*.*K*., *Pandey J*., *Khatri Chhetri N*.*B*. *et al*. *259* (E); Sankhuasabha district, Tinjure, 2800 m, *Kanai et al*. *721126* (TI); Sankhuwasabha district, Jaljale Himal, Shuwan Kharka-Topke Gola, 3570–4360 m, *Ohba H*. *et al*. *9120291* (TI).

***Impatiens gammiei*** Hook.f. Rec. Bot. Surv. India 4(2): 16, 21 (1905). Fig 5D.

Type: *Burkill I*. *H*. *27689* (neo, designated in [29], K K000694668!), Darjiling, Sabarkum, 11000 ft., 01 Oct. 1906.

*Impatiens minimiflora* Hook. f. Rec. Bot. Surv. India 4: 16, 21 (1905).

Type: *Clarke C*.*B*. *9874 B* (lecto, designated in [29] K K000694788!), Sikkim, Yakla, 11000 ft., 15 Oct. 1869.

Habitat: Temperate forest at an elevation of 3000–3600 m.

Distribution: Eastern Nepal and India

Specimen examined: Taplejung district, 3400 m, 08 Sept., 2011, *Bhakta B*. *Raskoti 201079* (KATH).

Note: *Impatiens gammiei* is very close to *Impatiens radiata* in overall morphology; only a notable difference is spurless lower sepal in former. More robust study is necessary for the confirmation of taxonomic identity of *Impatiens gammiei*.

**Fig 13:**
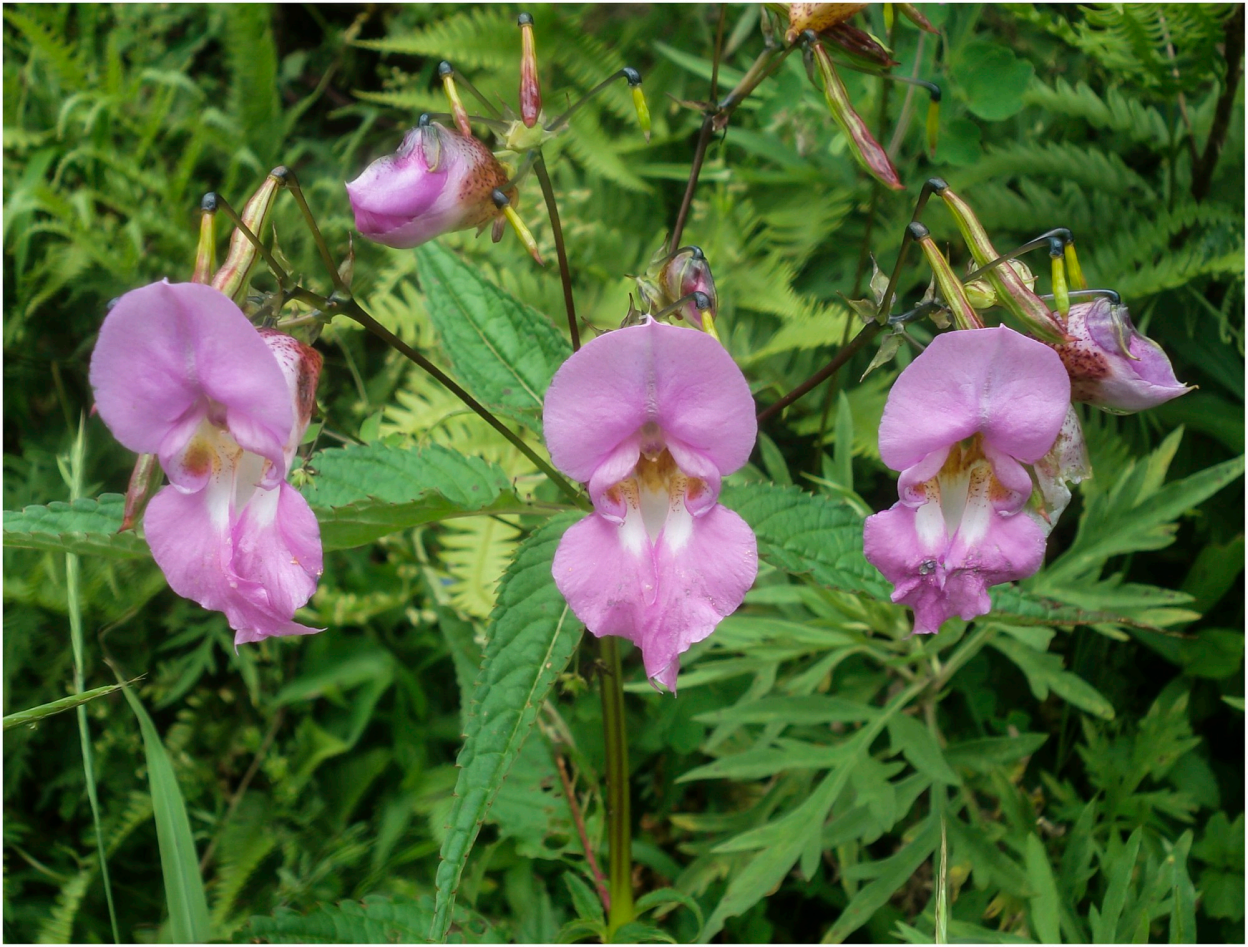
*Impatiens glandulifera* (flowering plant). Photograph by Bhakta Bahadur Raskoti.

***Impatiens glandulifera*** Royle, Ill. B. Him. t. 28, f. 2 (1834); 151 (1835). Fig 13.

Type: *Mts*. *t*. *28*, *f*. *2*. (designated in [32], Illustration Royle, Ill. Bot. Himal), 1835.

Habitat: Temperate forest at an elevation of 3000 m.

Distribution: Western Nepal, India and Pakistan.

Specimen examined: Harigaun, 3000 m, 12 Jul. 1952, *Polunin O*., *Sykes W*.*R*. *& Williams L*.*H*.*J*., *143* (TI).

***Impatiens glauca*** Hook.f. & Thomson, J. Linn. Soc. Bot. 4: 155. (1860).

Type: *Strachey & Winterbottom 14* (holo, K K000694742!), India, Kumaon ad Dwali, 9500 ft.

Habitat: Temperate forest at an elevation range of 2200–3000 m.

Distribution: Western Nepal and India.

Specimen examined: Bajura district, Damkane-Jilli, 1700 m, 13 Aug. 1991, *Rajbhandari K*.*R*. *14957* (KATH); Bajura district, Damkane, 2300 m, 13 Aug. 1991, *Suzuki M*., *Hatta H*., *Kurosaki N*., *Mikage M*., *Miyamoto F*., *Rajbhandari K*.*R*. *et al*. *70555* (TI); Bajura district, Porakya-Serigaon, 2200 m, 13 Aug. 1991, *Suzuki M*. *et al*. *9170555* (KATH); Below Garjigoth, Dori Lekh, N.W. of Jumla, 10000 ft., 09 Aug. 1952, *Polunin O*., *Sykes W*.*R*. *& Williams L*.*H*. *5052* (E); Ratamata Chakure Lekh, 10000 ft., 12 Aug. 1952, *Polunin O*., *Sykes W*.*R*. *& Williams L*.*H*. *405* (E).

**Fig 14:**
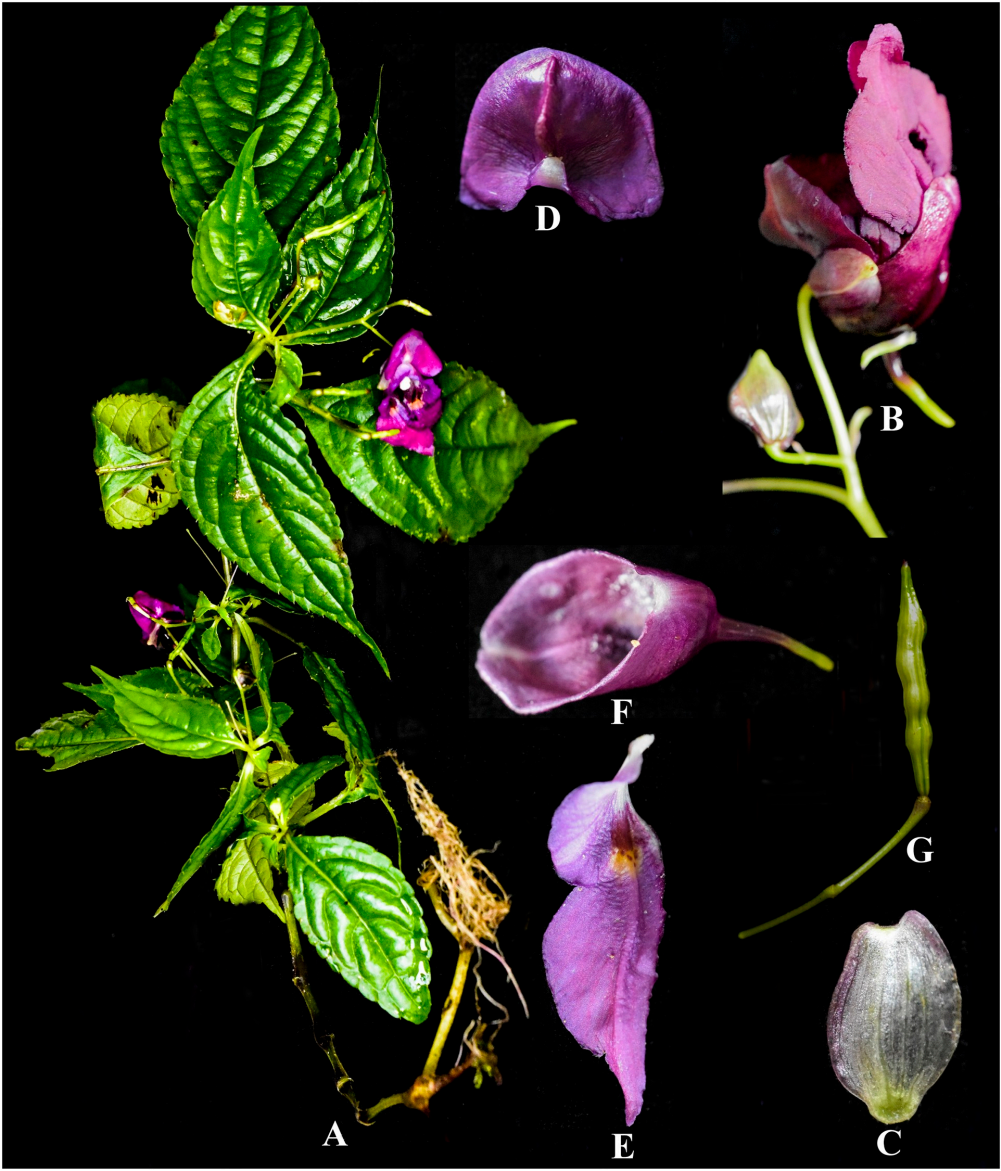
*Impatiens gorepaniensis* A. Flowering plant; B. Flower (side view); C. Lateral sepal; D. Dorsal sepal; E. Lateral united petal; F. Lower sepal; G. Capsule. Photographs by Bhakta Bahadur Raskoti.

***Impatiens gorepaniensis*** Grey-Wilson, Kew Bull. 44: 715 (1989). Fig 14.

Type: *Grey-Wilson*, *Phillips C*. *868* (K K000694667!), Nepal, west of Ghorepani, 10 Sept. 1973.

Habitat: Temperate forest at an elevation of 2000 m.

Distribution: Western Nepal (endemic).

Specimen examined: West of Gorepani, 2000 m, 10 Sept. 1973, *Grey-Wilson and Philips 868* (K).

Note: *Impatiens gorepaniensis* is morphologically similar to *Impatiens cymbifera*; further study including morphology and molecular data is required for the taxonomic identity of *Impatiens gorepaniensis*.

***Impatiens graciliflora*** Hook.f., Rec. Bot. Surv. India 4(2): 15 (1905).

Type: *Pantling 64I* (lecto, designated in [34] K K000694665!), India, Sikkim, Lenchul, 6000 ft., Aug. 1903.

Habitat: Temperate forest at an elevation range of 2000–2500 m.

Distribution: Eastern Nepal, Bhutan, India and Myanmar.

Specimen examined: Ilam district, Maipokhari, 4500 ft., Collector name not mention in sheet *15425* (KATH); Panchther district, Parangbung-Namle Phedi, 1700–2000 m, 25 Jun. 1992, *Noshiro S*., *Akiyama S*., *Acharya N*. *9241084* (TI).

Note: Based on morphological characters; *Impatiens graciliflora* is close to *Imapties radiata*; further study is necessary for the confirmation of taxonomic identity of *Impatiens graciliflora*.

**Fig 15:**
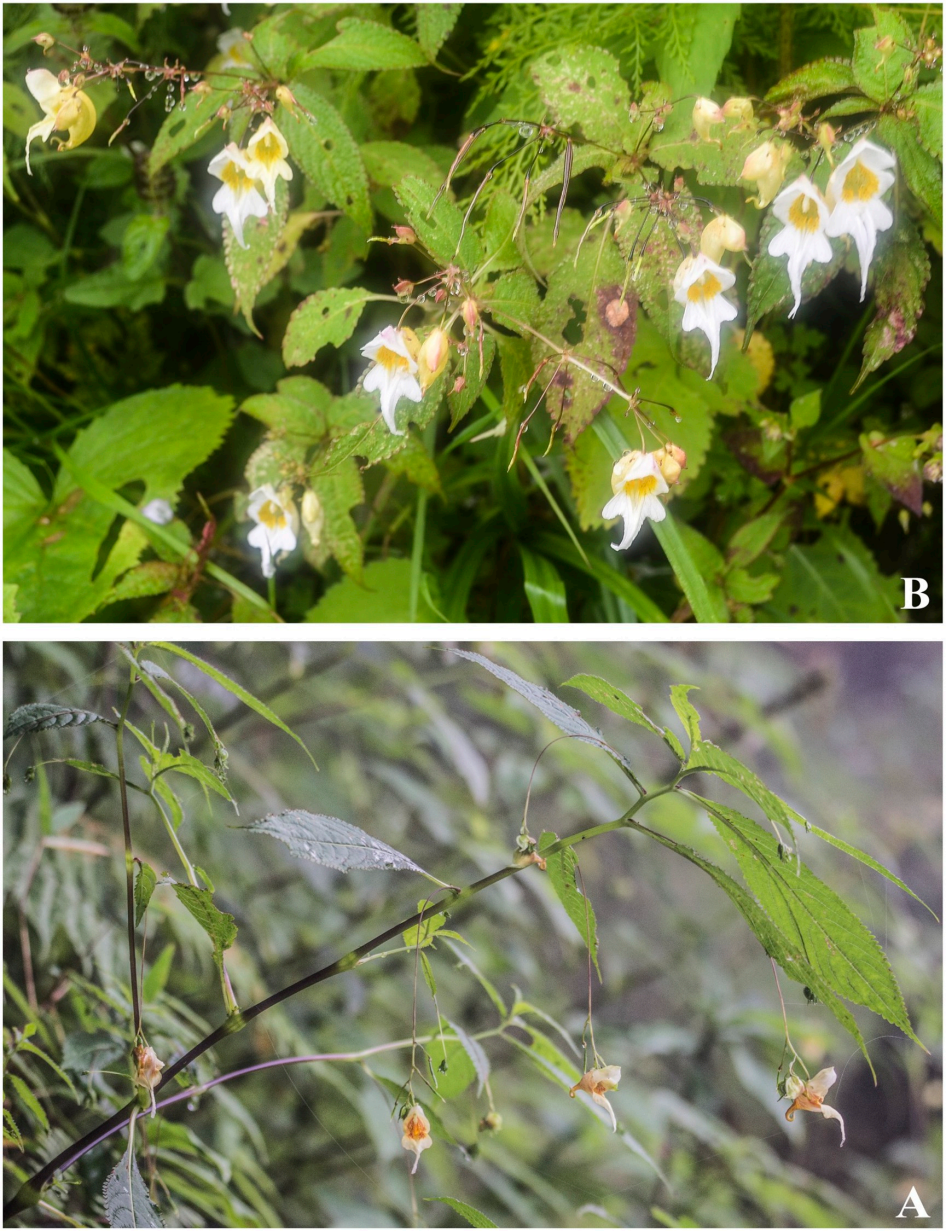
A, *Impatiens harae*; B. *Impatiens urticifolia*. Photographs by Bhakta Bahadur Raskoti.

***Impatiens harae*** H. Ohba & S. Akiyama, Journ. Jap. Bot. 62 (12): 368 (1987). Fig 15A.

Type: *Ohba H*. *et al*. *8530680* (holo, TI!), Nepal, Ramechhap district, Neju-Choarma, 02 Aug. 1985.

Habitat: Temperate forest at an elevation range of 2000–2500 m.

Distribution: Eastern, Central Nepal and China.

Specimen examined: Ramechhap district, Neju-Choarma, 3651–2760 m, 02 Aug. 1985, *Ohba H*. *et al*. *61078* (KATH); Ramechhap district, Choarma, 2760 m, 02 Aug. 1985, *Ohba H*., *Wakayabashi M*., *Suzuki M*., *Kurosaki N*., *Rajbhandari K*.*R*. *and Wu S*.*K*. *8530680* (TI).

***Impatiens hobsonii*** Hook. f., Rec. B. Surv. Ind. 4: 15 & 20 (1905), ’hobsoni’. H. Hara in H. Hara & Williams, Enum. Flow. Pl. Nep. 2: 79 (1979). Fig 8D.

Type: *Hobson H*. *E*. *s*.*n*. (lecto, designated in [29], K K000694824!), India, Sikkim, Lachen valley, Yatung, 1897.

Habitat: Temperate forest at an elevation range of 3300–3500 m.

Distribution: Eastern Nepal and India.

Specimen examined: Sankhuwasabha district, Mahang khola, 11000 ft., 02 Jul. 1956, *Satinton J*.*D*.*A*. *828* (KATH); Sankhuwasabha district, Arun Valley, Maghang Khola, 11000 ft., *Stainton 828* (BM); Milke Dande, 9000 ft., *Beer 10051* (BM); Sankhuwasabha district, Wabak Khola, 11500 ft., *Beer 9467* (BM).

**Fig 16:**
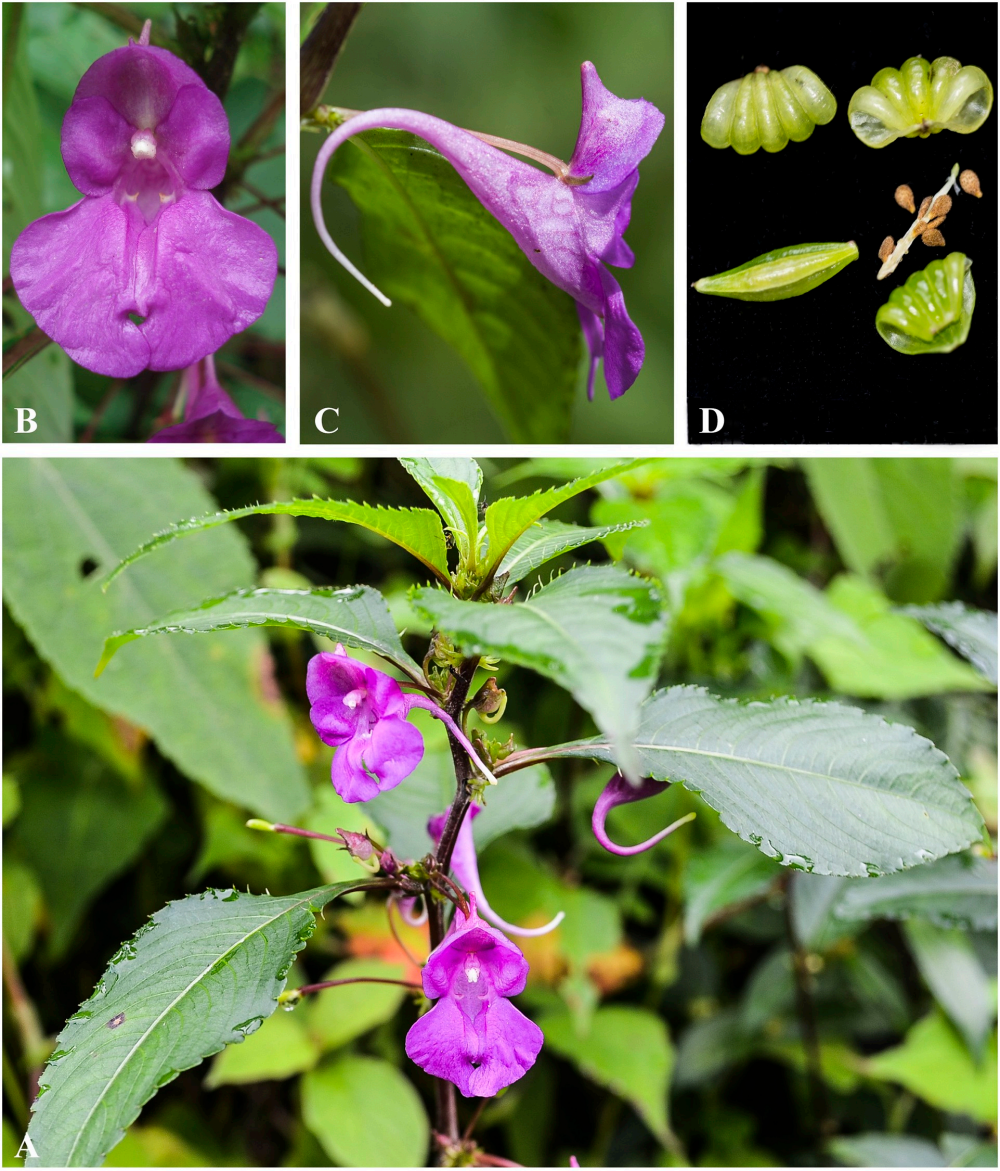
*Impatiens infundibularis* A. Flowering plant; B. Flower (front view); C. Flower (side view); D. Capsule and seeds. Photographs by Bhakta Bahadur Raskoti.

***Impatiens infundibularis*** Hook. f., Rec. Bot. Surv. India 4: 13, 19. 1905. Fig 16.

Type: *Hooker J*.*D*. *s*.*n*. (lecto, designated in [29], left hand side specimen of K K000694661!, India, Sikkim, 2000–5000 ft.; isolecto CAL, right hand side specimen of K K000694661).

Habitat: Sub-tropical forest at an elevation of 1400 m.

Distribution: Eastern Nepal, Bhutan and India.

Specimen examined: Ilam district, Kolbung, 1400 m, *Bhakta B*. *Raskoti 20121* (KATH).

**Fig 17:**
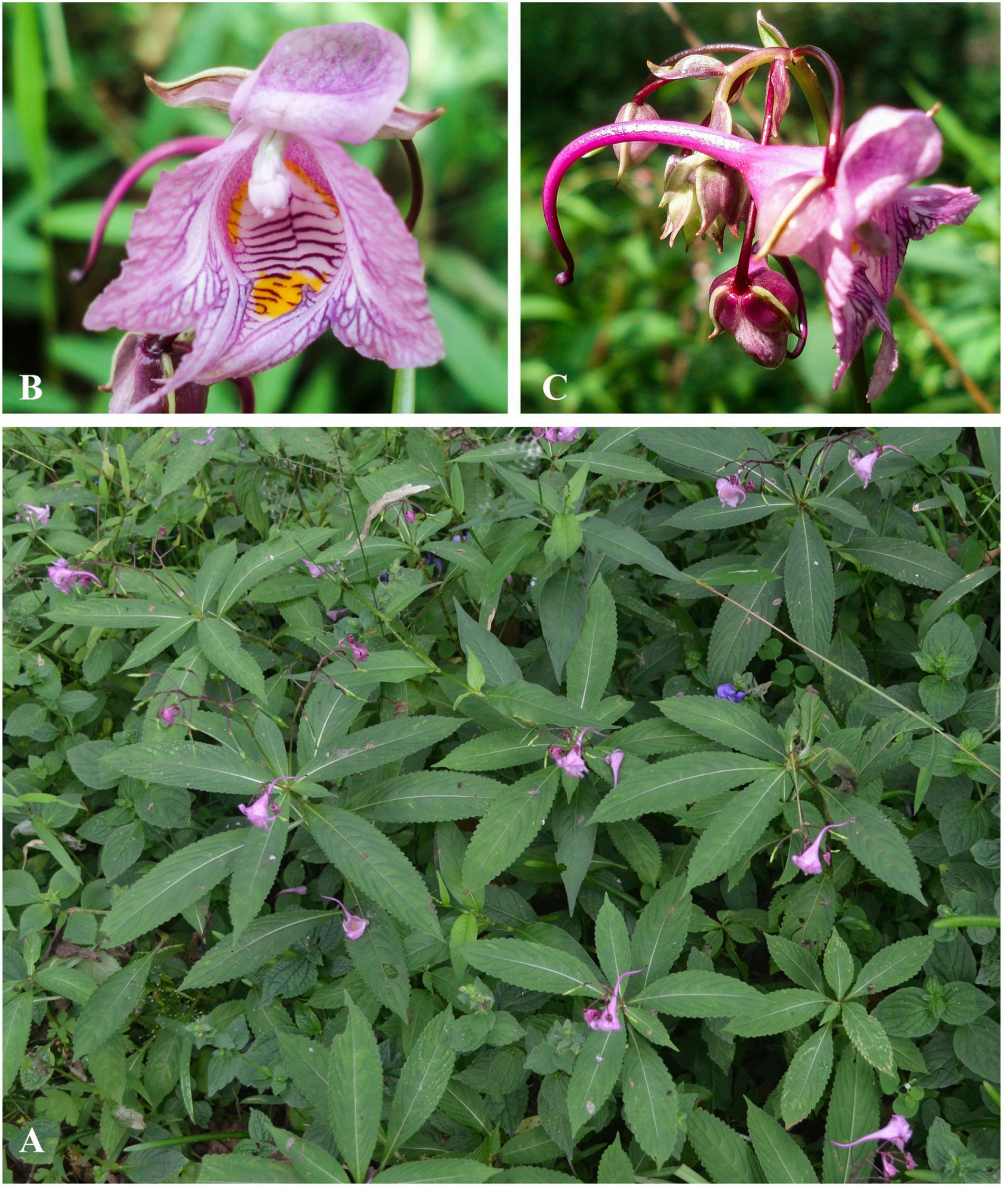
*Impatiens insignis* A. Flowering plant; B. Flower (front view); C. Flower (side view). Photographs by Bhakta Bahadur Raskoti.

***Impatiens insignis*** DC., Prodr. 1: 688 (1824). Fig 17.

Type: *Wallich s*.*n*. (lecto, designated in [32], G00218231), Napauliâ, 1821; (syn, G00218230).

Habitat: Tropical to temperate forest at an elevation range of 900–2000 m.

Distribution: Eastern, Central Nepal and India.

Specimen examined: Taplejung district, Heydewa-Garhi danra, 03 Nov. 1963, *Hara H*. *et al*. *6300502* (KATH); Kule khani, 1450 m, 02 Sept. 1970, *Kani H*. *& Shrestha T*.*B*. *672705* (KATH); Baju danda to Tamor river, 1550 m, 31 Aug. 1989, *Kew-Edinburgh-Kathmandu Expedition to N*.*E*. *Nepal 143* (E); Terhathum district, 1650 m, 11 Sept. 1967, *Williams & Stainton 8453* (TI); Kathmandu district, Sundarijal Waterfall, 1600 m, 20 Sept. 1963, *Hara H*. *et al*. *6306724* (TI); Lalitpur district, Godavari, 1500 m, 18 Sept. 1963, *Hara H*. *et al*. *6306723* (TI); Phulchoki, 1800 m, 28 Aug. 1969, *Miss Manandhar & party 12915* (TI); Makwanpur district, Okhre Danra–Deorali, 1600 m, 02 Sept. 1970, *Kanai H*. *& T*.*B*. *Shrestha 672705* (TI); Sankhuwasabha district, Dumhan, 1200 m, 30 Oct. 1963, *Hara H*. *et al*. *s*.*n*. (TI); Sindhupalchok district, Charikot–Mokaibari, 30 Sept. 1970, *Kanai H*. *et al*. *674699* (TI); Taplejung district, Dumhan–Taplejung, 1400 m, 01 Nov. 1963, *Kanai H*. *et al*. *6306725* (TI); Papung-Sangrati Pati, 1500 m, 26 Aug. 1977, *Ohashi H*. *et al*. *772736* (TI); Taplejung district, Heydewa-Garhi Danra, 03 Nov. 1963, *H*. *Hara et al*. *6300502* (TI); Taplejung district, Tuwa-Taplethok, 05 Nov. 1963, *Hara H*. *et al*. *s*.*n*. (TI).

***Impatiens jurpia*** Buch.-Ham., J. Proc. Linn. Soc., Bot. 4: 140 (1859). Fig 8A.

Type: *Wallich Herb*. *no*. *4761* (lecto, designated in [35], A, K-W K001039839!), Nepal, Morang Hills, 14 Jul. 1810.

Habitat: Subtropical forest at an elevation range of 1500–1800 m.

Distribution: Eastern, Central Nepal, India and Myanmar.

Specimen examined: Lalitpur district, Phulchoki, 2200–2700 m, 09 Aug. 1969, *Kani H*. *673373* (KATH); Gorkha district, Dovan-Jagat, 1220 m, 27 Jul. 2007, *Ikeda H*., *Kawahara T*., *Yano O*., *Watson M*.*F*., *Li Z*.*H*., *Subedi M*.*N*., *et al*. *20816035* (KATH); Sanguri Bhanjang-Dhara Pani, *Hara H*. *et al*. *6300490* (TI); South of Hatora, 700 m, *Grey-Wilson & Philllips 67A* (K).

***Impatiens kharensis*** S. Akiyama, H. Ohba & Wakab., Himal. Pl. 2: 75 (1991).

Type: *Ohba H*. *et al*. *8320702* (holo TI!, iso KATH!), Nepal, Khare Khola, Bitta Kharka (4100 m) -a valley (3300 m)-Patale Pokhari, 4000 m, 12 Sept. 1983.

Habitat: Temperate forest to alpine slopes at an elevation range of 2500–4000 m.

Distribution: Central Nepal (endemic).

Specimen examined: Dolakha district, Vitte kharka-Valley patale pokhari, 3300–4000 m, 12 Sept. 1983, *Ohba H*., *Wakabayashi M*., *Suzuki M*., *Akiyama S*. *8320702* (TI); Dolakha district, Vitte kharka-Valley patale pokhari, 3300–4000 m, 12 Sept. 1983, *Ohba H*., *Wakabayashi M*., *Suzuki M*., *Akiyama S*. *8341278* (TI).

***Impatiens laevigata*** Wall. ex Hook. f. & Thomson, J. Linn. Soc. Bot. 4: 146 (1860).

Type: *Wall*. *Cat*. *Num*. *list no*. *4753* (K K000694815!), Sillet, 1820.

Habitat: Subtropical forest at an elevation range of 1000–1500 m.

Distribution: Nepal, India and Myanmar.

Note: Though, this speicies is included in the literature [8], authors are unable to track authentic specimen of *Impatiens laevigata* from Nepal. Further study is necessary for the existence of this species within Nepal.

**Fig 18:**
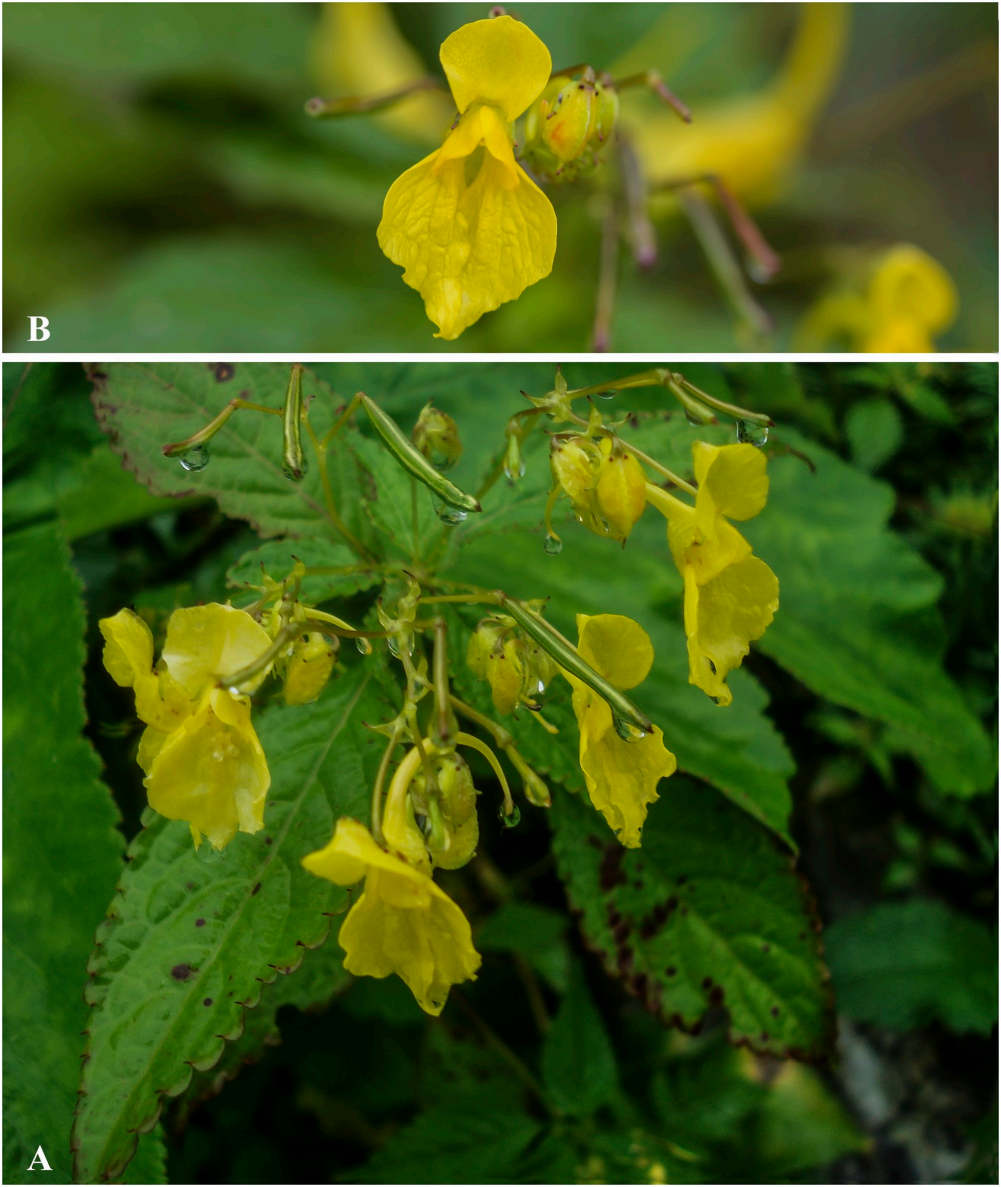
*Impatiens laxiflora* A. Flowering plant; B. Flower (front view). Photographs by Bhakta Bahadur Raskoti.

***Impatiens laxiflora*** Edgew., Tr. Linn. S. 20: 40 (1846). Fig 18.

Type: *Edgeworth M*.*P*. *399* (holo, K K000694834!), India, Serain, 8000–10000 ft., 1844.

Habitat: Temperate to subalpine forest at an elevation range of 2700–3900 m.

Distribution: Eastern, Central, Western Nepal, China and India.

Specimen examined: Lumsa north west of Jumla, 9500 ft., 10 Aug. 1952, *Stainton*, *Sykes & Williams 5117* (E); Barabar, 5000 ft., 20 Aug. 1935, *Bailey’s F*.*M*. (BM); Bajhang district, Khaptad National Park, 2900 m, 27 Aug. 1991, *Suzuki M*. *et al*. *9170978* (TI); Porakya-Serigaon, 2200 m, 13 Aug. 1991, *Suzuki M*. *et al*. *9170552* (TI); Jumla district, Below Gajigoth, Dori Sekh, 10000 ft., 09 Aug. 1952, *Polumin*, *Sykes & Williams 5054* (BM); Rukum district, East of Chalike Pahar, 05 Aug. 1954, *Stainton*, *Sykes & Williams 3755* (E); Sankhuwasabha district, Barun khola, Sana Pokhari, 3300 m, 22 Aug. 1981, *Grey-Wilson*, *Henderson*, *Rajbhandary & Taylor 4149* (K); Sankhuwasabha district, Barun khola, W. of Yangle, 3500 m, 22 Aug. 1981, *Grey-Wilson*, *Henderson*, *Rajbhandary & Taylor 423* (K).

***Impatiens leptocarpa*** Hook. f., Rec. Bot. Surv. India 4(2): 17, 22 (1905).

Type: *Prain’s collector 511* (K), Sikkim, Bhalbort, Oct. 1903.

Habitat: Temperate to lower belt of subalpine zone at an elevation ranges of 2600–3600 m.

Distribution: East Nepal and India.

Specimen examined: Sankhuwasabha district, north of sando pokhari near barun khola, 3400 m, 22 Aug. 1981, *Grey-Wilson Henderson 4141* (E).

Note: This species is not well documented; it was collected from Phalut (Darjeeling, India), which is close to the boarder of eastern Nepal. Literature [36] included a photograph of *Impatiens leptocarpa* taken in Ramitay Odaar, Sankhuwasabha, Nepal. We recommend its further study and collections from Nepal.

**Fig 19:**
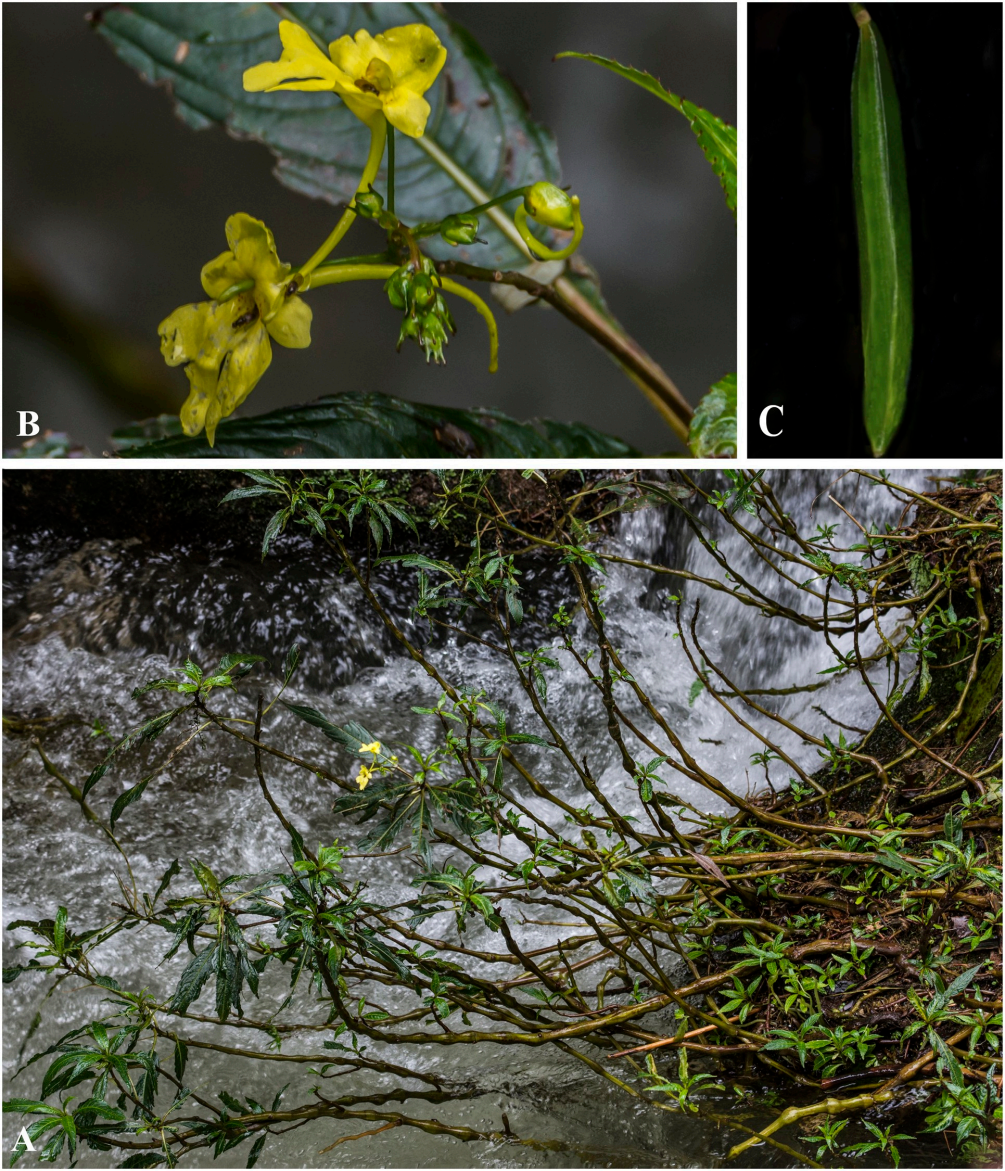
*Impatiens leptoceras* A. Flowering plant; B. Flower; C. Capsule. Photographs by Bhakta Bahadur Raskoti.

***Impatiens leptoceras*** DC., Prodr. 1: 688 (1824). Fig 19.

Type: *Wallich s*.*n*. (lecto, designated in [29], G00218028), Nepal, 1819.

Habitat: Subtropical forest at an elevation range of 1600–1800 m.

Distribution: Central Nepal, China and India.

Specimen examined: Sundarijal, 5600 ft., 14 Sept. 1977, *Pradhan P*. *& Shrestha N*. *127* (KATH); Naghmeti khola Sundarijal area, 4600 ft., 19 May 1976, *Amatya M*.*M*., *Sharma R*. *& Shrestha R*. *173/76ms* (KATH); Kathmandu district, Thage, 1800 m, 01 Sept. 1970, *Kani H*. *& Shrestha T*. *B*. *672694* (TI).

***Impatiens nimspurjae*** sp. nov.

Type: *Bhakta B*. *Raskoti* 201190 (holo, KATH; iso, TUCH), Nepal, Myagdi District, above Moreni, 2800–2900 m, 25 Aug. 2011.

Habitat: Temperate forest and forest margins at an elevation range of 2800–2900 m.

Distribution: Western Nepal (endemic).

Specimen examined: Only examined type specimen *Bhakta B*. *Raskoti 201190* (KATH).

***Impatiens occultans*** Hook. f., Rec. B. Surv. Ind. 4: 17 & 22 (1905).

Type: *Gammie 414* (lecto, designated in [33] K K000694783!), India, Sikkim, 12000–13000 ft.

Habitat: Temperate forest and alpine grasslands at an elevation range of 3350–4000 m.

Distribution: Eastern, Central Nepal, China and India.

Specimen examined: Solukhumbu district, Randu Kharka, 3350 m, 09 Aug. 1997, *Rajbhandari K*.*R*. *9740196* (KATH); Ramechhap district, Neju, 3651 m, 31 Jul. 1985, *Ohba H*. *& Wu S*.*K*. *8580614* (KATH); Solukhumbu district, Pike dongshar, 3600 m, 09 Sept. 1985, *Botanical Expedition to Himalaya (1985) 8531381* (E), Rasuwa district, Singum gumpa-Gosaikunda, 3900 m, 23 Aug. 1972, *Hara H*. *et al*. *721860* (TI); Ramechhap district, Neju, 3651 m, 31 Jul. 1985, *Botanical Expidition to Himalaya* (1985) (BM).

***Impatiens porrecta*** Wall. ex Hook. f. & Thomson, J. Linn. S. B. 4: 138 (1860).

Type: *Wall*. *Cat*. *no*. *7275* (lecto, designated in [26], K K001127061!), Sylhet.

*Impatiens bella* Hook. f. & Thomson, J. Proc. Lin. Soc. Bot. 4(15):138 (1859).

Type: *Griffith s*.*n*. (lecto, designated in [26], K K000694775!), Khasia.

Habitat: Temperate forest at an elevation of 3000 m.

Distribution: Nepal, India and Manipur.

***Impatiens pradhanii*** H. Hara, J. Jap. Bot. 40: 99 (1965).

Type: *Pradhan K*.*C*. *& Hara H*. *6306729* (holo TI!), India, Sikkim, Lagyap, 13 Sept. 1964.

Habitat: Temperate forest at an elevation ranges of 2000–3200 m.

Distribution: Central Nepal and India

Specimen examined: Gorkha district, Ghap to Namrung, 2280 m, 31 Jul. 2008, *Ikeda H*. *et al*. *20811094* (KATH); Dolakha district, Jagat-Samagaun, 2030 m, 15 Oct. 2007, *Noshiro et al*. *20710078* (KATH); Manang district, Marsyandi Khola Chame, 2630 m to Dhanagyang, 2200 m, 06 Aug. 1983, *Ohba H*. *et al*. (TI); Dolakha district, Rolwaling Khola: Simigaon, 1950 m to Kyalche, 2700 m, 31 Aug 1983, *Ohba H*. *et al*. (TI); Kyalche, 2700 m to Thandingma, 3200 m, 01 Sept. 1983, *Ohba H*. *et al*. (TI).

***Impatiens prainii*** Hook.f., Rec. Bot. Surv. India 4(2): 14, 20 (1905).

Type: *Prain 377* (lecto, designated in [37], K K000694764!; isolecto K! sheet 1 with illustration, K sheet 2), India, eastern Himalaya, Sikkim, Lachung Valley, near Choongtang.

*Impatiens mallae* S. Akiyama & H. Ohba, Journ. Jap. Bot. 67: 187 (1992).

Type: *Suzuki et al*. *no 8821106* (holo, TI!; iso, KATH!), Nepal, Koshi, Sankhuwasabha, Tashi Gaun, 2160–3500 m.

Habitat: Temperate forest at an elevation range of 2100–3500 m.

Distribution: Eastern Nepal, China and Myanmar.

Specimen examined: Solukhumbu district, Tasigaun-Kauma, 2590 m, 25 Sept., 1991, *Long D*.*G*. *et al*. *241* (KATH).

**Fig 20:**
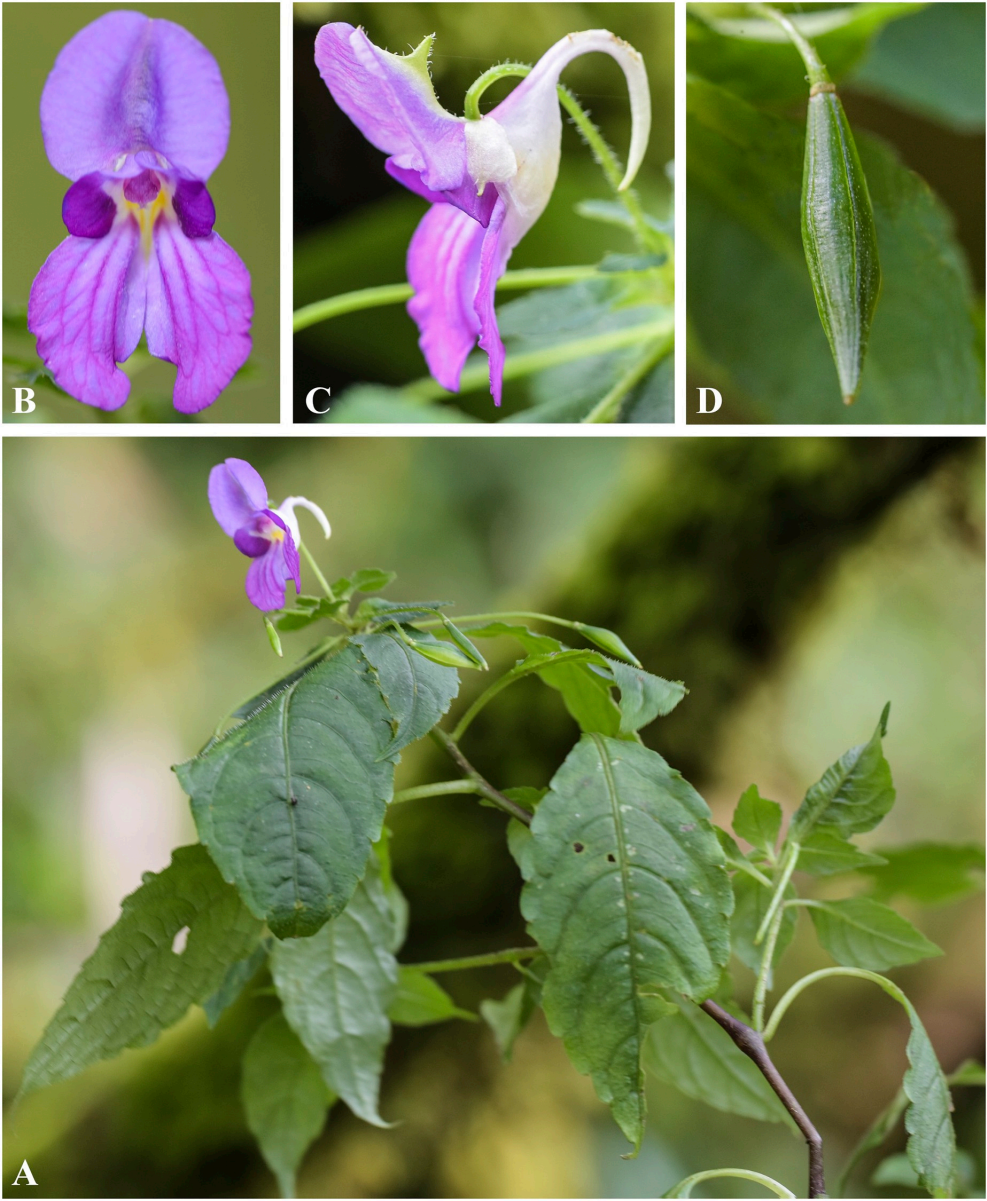
*Impatiens puberula* A. Flowering plant; B. Flower (front view); C. Flower (side view); D. Capsule. Photographs by Bhakta Bahadur Raskoti.

***Impatiens puberula*** DC., Prodr. 1: 687 (1824). Fig 20.

Type: *Wallich N*. (holo G00218032), Nepal, Napaulia.

*Impatiens mollis* Wall., Fl. Ind. (Carey and Wallich ed.) 2: 461 (1824).

Type: *Wallich N*. *s*.*n*., Sheopore Nipal (Nepal).

Habitat: Tropical to temperate forest at an elevation range of 1500–2700 m.

Distribution: Eastern, Central Nepal, Bhutan, China and India.

Specimen examined: Ramechhap district, Choarma, 2760 m, 03 Aug. 1995, *Ohba H*. *et al*. *61154* (KATH); Sankhuwasabha district, ArunValley, Chhoyang Khola, 2550 m, *Stainton J*.*D*.*A*. *699* (TI); Lalitpur, Phulchoki, 1800 m, 27 Aug. 1965, *Shilling A*. *D*. *618* (TI); Taplejung district, Yamphodin, 2100 m, 25 Jun. 1969, *Williams L*. *H*. *J*. *941* (TI); Dhankuta district, Sedua, 1740 m, 15 Sept. 1971, *Ohsawa M*. *& Shakya P*.*R*. *1799* (TI); Terhathum district, Tute-Tinjure Phedi, 14 Jul. 1991, *Ohba H*. *et al*. *9120057* (TI); Terhathum district, Tinjure Danda, 7500 ft., 06 Sept. 1967, *Williams & Stainton 8378* (TI); Dolakha district, Jiri-Sibalaya, 2250 m, 08 Jul. 1995, *Miyamoto F*. *et al*. *9596013* (TI); Kaski district, Banthanti-Ghodepani Deurali, 2720 m, 24 Aug. 1988, *Suzuki M*. *et al*. *8842103* (TI); Kaski district, Ghandruk–Bhainsi Kharka 2160 m, 22 Aug. 1988, *Suzuki M*. *et al*. *8860585* (TI); Kathmandu district, Sheopuri, 6500 ft., 19 Aug. 1954, *Stainton*, *Sykes & Williams 6936* (E); Lalitpur district, Phulchoki, 09 Aug. 1969, *Kanai H*. *673348* (TI); Myagdi district, Chitre, 2150 m, 27 Aug. 1994, *Mikage M*. *et al*. *9485557* (TI); Ghar Khola, 3000 m, *Stainton*, *Sykes & Williams 5777* (E); Panchthar district, Prang–Goruwale Bhanjang, 25 Jun. 1992, *Noshiro S*. *et al*. *9241085* (TI); Sindhupalchok district, Thale–Thale Bisauna, 2500 m, 10 Sept. 1970, *Kanai H*., *Ch*. *Chuma & T*. *Nagano s*.*n*. (TI); Solukhumbu, Nunthala–Junbesi, 2620 m, 31 Aug. 1997, *Wakabayashi M*. *et al*. *9710412* (TI); Solukhumbu, Nunthala-Kharikhola, 1670 m, 27 Jul. 1995, *Miyamoto F*. *et al*. *9596158* (TI); Taplejung district, Shewaden-Mewa Khola, 2300 m, 29 Jun. 1972, *Kanai H*. *et al*. *720947* (TI).

***Impatiens pulchra*** Hook. f. & Thomson, J. Linn. S. B. 4: 139 (1860).

Type: *Hooker J*. *D*. *& Thomson T*. *2281* (lecto, desiginated in [26], K K000694754!; isolecto, K K0006947530!), India, Khasia, elevation 4000–5000 ft.

Habitat: Temperate forest at an elevation range of 2400–2700 m.

Distribution: Eastern Nepal, China and India.

Specimen examined: East Nepal, Moyong valley, 4000–5000 ft., *Hooker J*.*D*. *s*.*n*. (K).

**Fig 21:**
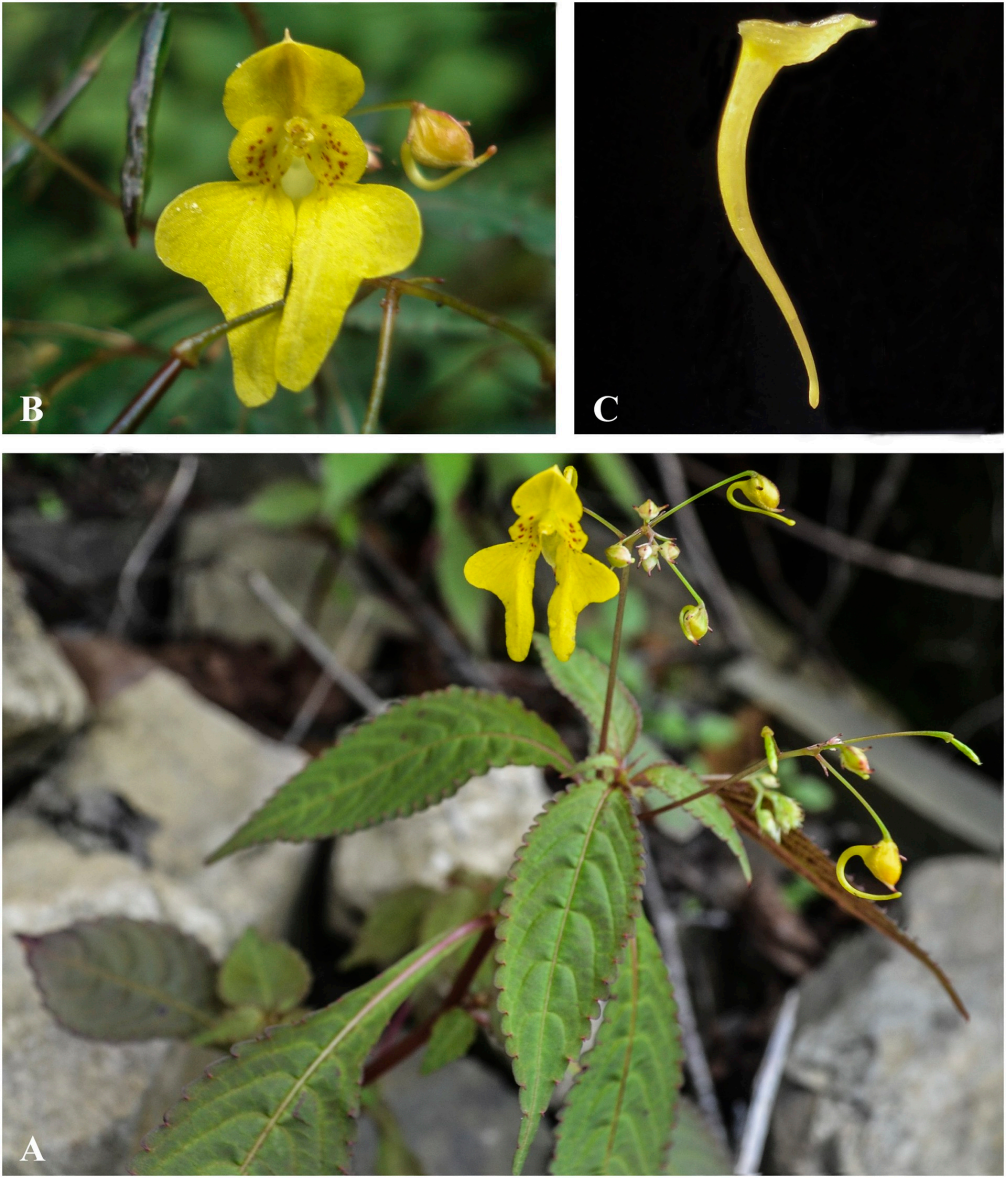
*Impatiens racemosa* A. Flowering plant; B. Flower (Front view); C. Lower sepal (side view). Photographs by Bhakta Bahadur Raskoti.

***Impatiens racemosa*** DC., Prodr. 1: 688 (1824). Fig 21.

Type: *Wallich s*.*n*. (holo, G00218030), Nepal (Napaulia), 1821.

*Impatiens racemosa* DC. var. *ecalcarata* Hook.f., Rec. Bot. Surv. India. 4: 9 (1905).

Type: *Prain 39I* (lecto, designated in [26] K K000694752!), India, Sikkim Himalaya.

*Impatiens microsciadia* Hook.f., Rec. B. Surv. Ind. 4(2): 16 & 21 (1905).

Type: *Pantling s*.*n*. (lecto, designated in [26] with illustration by Hooker, K), Sikkim, 6000 ft.

Habitat: Tropical to sub-alpine forest at an elevation range of 1300–3900 m.

Distribution: Eastern, Central, Western Nepal, Bhutan, China, India, Myanmar and Thailand.

Specimen examined: Dolakha district, Lamabagar-Hum, 2000 m, 16 Jul. 1977, *Rajbhandari K*.*R*. *& Roy B*. *1451* (KATH); Bajura district, Pategaon–Badigaon, 2700 m, 16 Aug. 1991, *Suzuki M*. *et al*. *9170710* (TI); Dhankuta district, Chauke-Tute, 2600 m, 01 Sept. 1977, *Ohashi H*. *et al*. *771266* (TI); Dhankuta district, Chitre, 2300 m, 05 Jun. 1972, *Kanai H*. *et al*. *720200* (TI); Gorkha district, Ekle Ghar-Sardu Khola, 2000 m, 25 Jul. 1994, *Suzuki M*. *et al*. *9470180* (TI); Gorkha district, Sardu Khola-Ripche, 2060 m, 27 Jul. 1994, *Suzuki M*. *et al*. *9480052* (TI); Kaski, Banthanti-Ghodepani Deurali, 2700 m, 24 Aug. 1988, *Suzuki M*. *et al*. *8881302* (TI); Kaski district, Banthati, 2360 m, 28 Aug. 1994, *Mikage M*. *et al*. *9485568* (TI); Pothana-Tolka, 1800 m, 20 Aug. 1988, *Suzuki M*. *et al*. *8881086* (TI); Kathmandu district, Shiwapuri Summit–Borlang Bhanjyng, 2732 m, 25 Aug. 1990, *Minaki M*. *et al*. *9011018* (TI); Lalitpur district, Phulchoki, 19 Sept. 1970, *Kanai H*. *& Ch*. *Chuma 675184* (TI); Makwanpur district, Kuli Khani, 1450 m, 03 Sept. 1970, *Kanai H*. *& T*.*B*. *Shrestha 672733* (TI); Manang district, Koto, 2440 m, 14 Aug. 1994, *Mikage M*. *et al*. *9485418* (TI); Mustang district, Kalopani–Tatopani, 1870 m, 23 Aug. 1999, *Mikage M*. *et al*. *9961317* (TI); Myagdi district, Shika–Narchang, 2200 m, 26 Aug. 1988, *Suzuki M*. *et al*. *8881498* (TI); Panchthar district, Gairi Kharka–Beteni, 21 Jun. 1992, *Noshiro S*. *et al*. *9240982* (TI); Rasuwa district, Dhunche–Grang, 2000 m, 01 Aug. 1995, *Hoshino T*. *et al*. *9539277* (TI).

**Fig 22:**
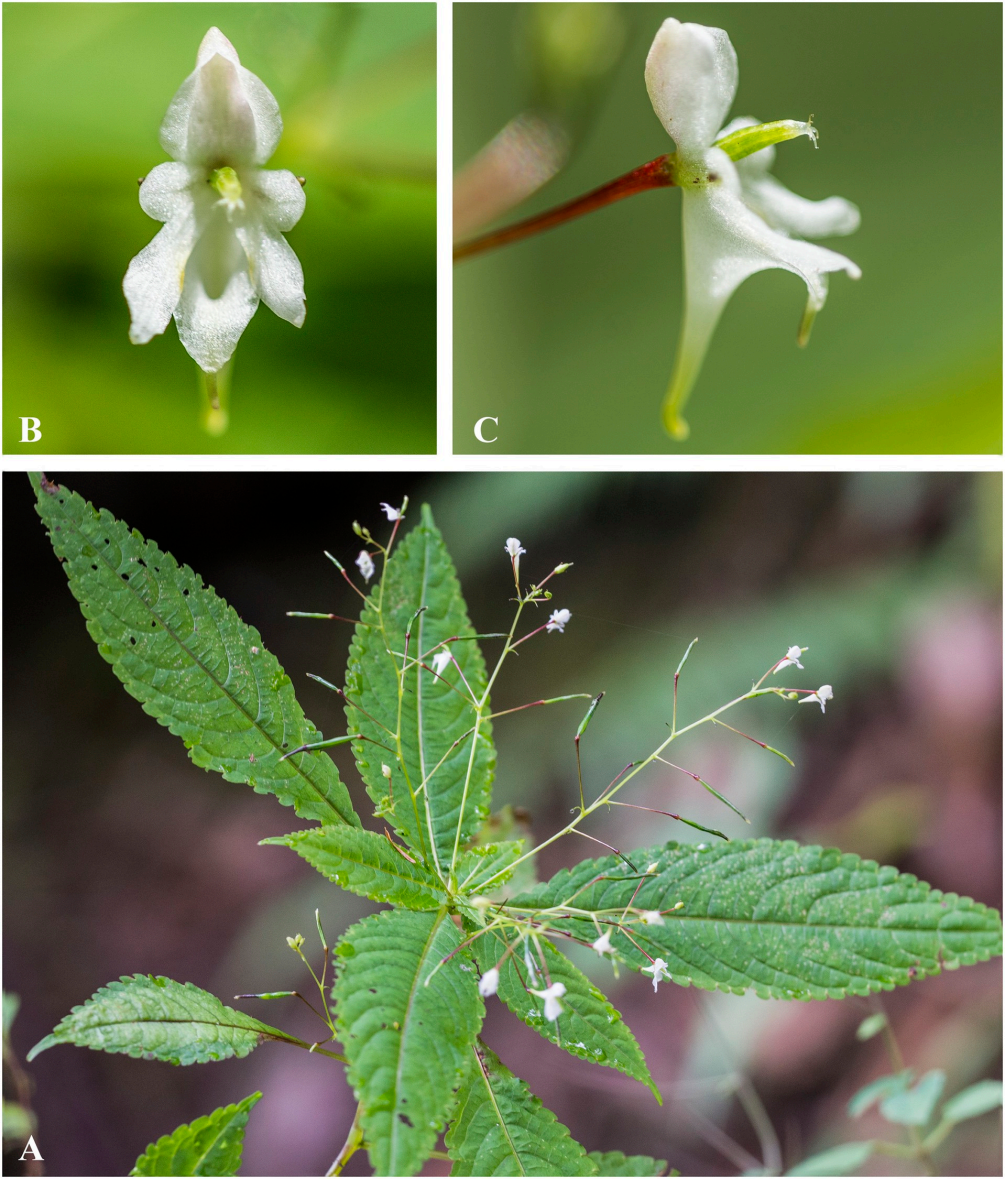
*Impatiens radiata* A. Flowering plant; B. Flower (Front view); C. Flower (side view). Photographs by Bhakta Bahadur Raskoti.

***Impatiens radiata*** Hook. f., Fl. Br. Ind. 1: 476 (1875). Fig 22.

Type: *Hooker s*.*n*. (lecto, designated in [34], K K000694747!; isolecto K K000694746!), India, Sikkim, Lachung, elevation 6000–12000 ft, 06 Jul. 1849.

Habitat: Temperate forest at an elevation range of 2100–3500 m.

Distribution: Eastern, Central Nepal, Bhutan, China, India and Thailand.

Specimen examined: Solukhumbu district, Beni kharka-Ringmo, 2600 m, 03 Sept. 1985, *Ohba H*. *et al*. *62005* (KATH); Lampokhari-Chitre, 9500 ft., 12 Aug. 1971, *Shrestha T*.*B*. *& Joshi D*.*P*. *533* (KATH); Ramechhap district, Neju, 19 Aug 1995, 3651 m, *Ohba H*. *et al*. 8571822 (KATH); Gorkha district, Lho- Samagaun, 3200 m, 02 Aug. 2008, *Ikeda H*., *Kawahara T*., *Yano O*., *Watson M*.*F*., *Li Z*.*H*., *Subedi M*.*N*. *et al*. *20811128* (E).

**Fig 23:**
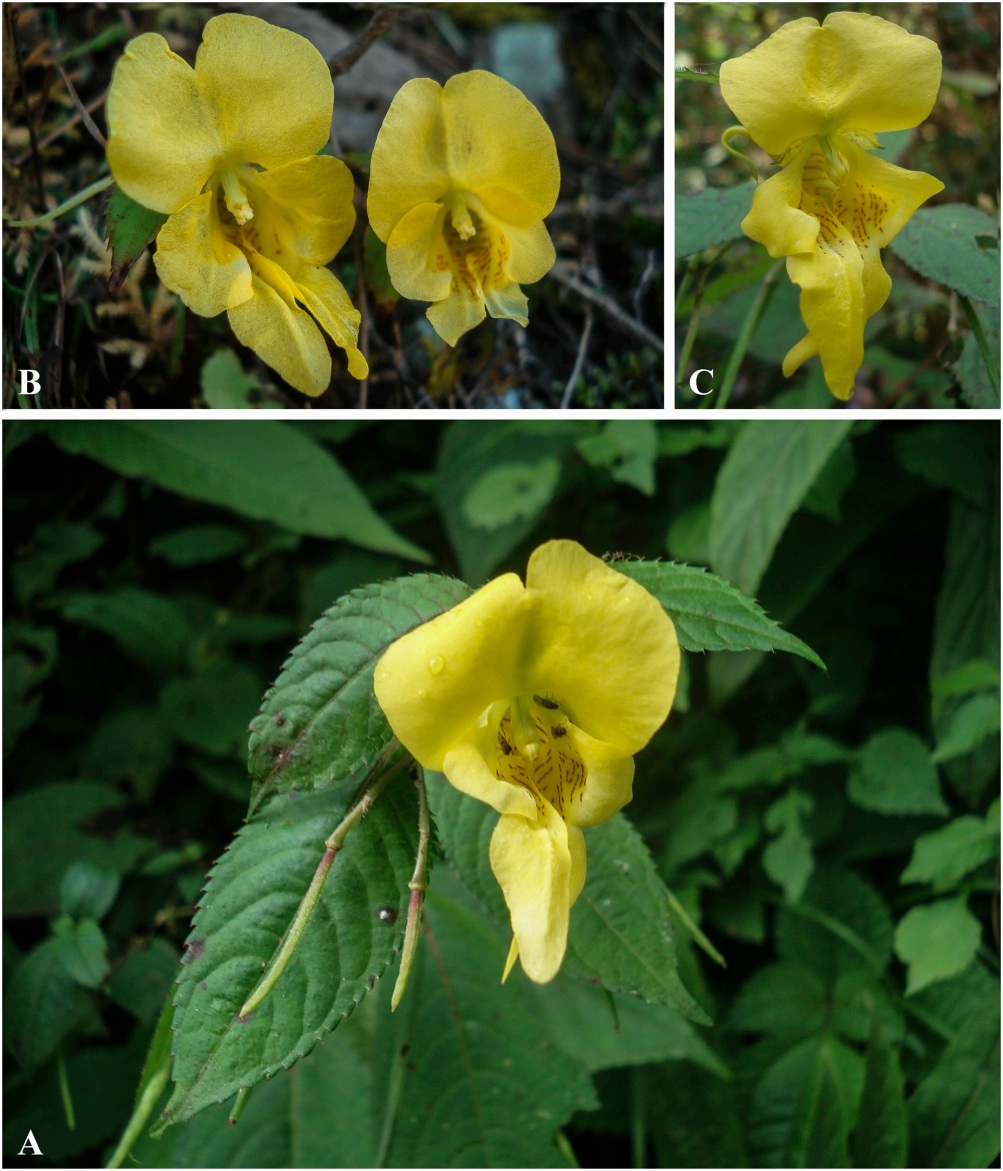
*Impatiens scabrida* A. Flowering plant; B, C. Flower (Front view). Photographs by Bhakta Bahadur Raskoti.

***Impatiens scabrida*** DC., Prodr. 1: 687 (1824). Fig 23.

Type: *Wallich N*. (holo, G00218814), Nepal (Napaulia).

*Impatiens calycina* Wall., Fl. Ind. (Carey & Wallich ed.) 2: 463 (1824).

Type: *Wallich N*. *4769a* (lecto, designated in [38]), Nepal, Chandragiri, Aug. 1821.

Habitat: Tropical to temperate forest at an elevation range of 1000–3600 m.

Distribution: Eastern Central, Western Nepal, Bhutan, China, Pakistan and India.

Specimen examined: Doti district, Khaptad lekh, 2950 m, 01 Jul. 1981, *Shakya P*.*R*., *Sharma L*.*R*. *& Amatya K*.*R*. *6255* (KATH); Mugu district, Kamali valley near lumsa, 7000 ft., 14 Aug. 1952, *Polunin*, *Shykes & Williams 5186* (KATH); Gorkha district, Dovan to Jagat, 1240 m, 27 Jul. 2008, Gorkha district, Dobhan to Jagat, 1240 m, 27 Jul. 2008, *Ikeda H*., *Kawahara T*., *Yano O*., *Watson M*.*F*., *Li Z*.*H*., *Subedi M*.*N*., *et al*. *20811050* (E).

**Fig 24:**
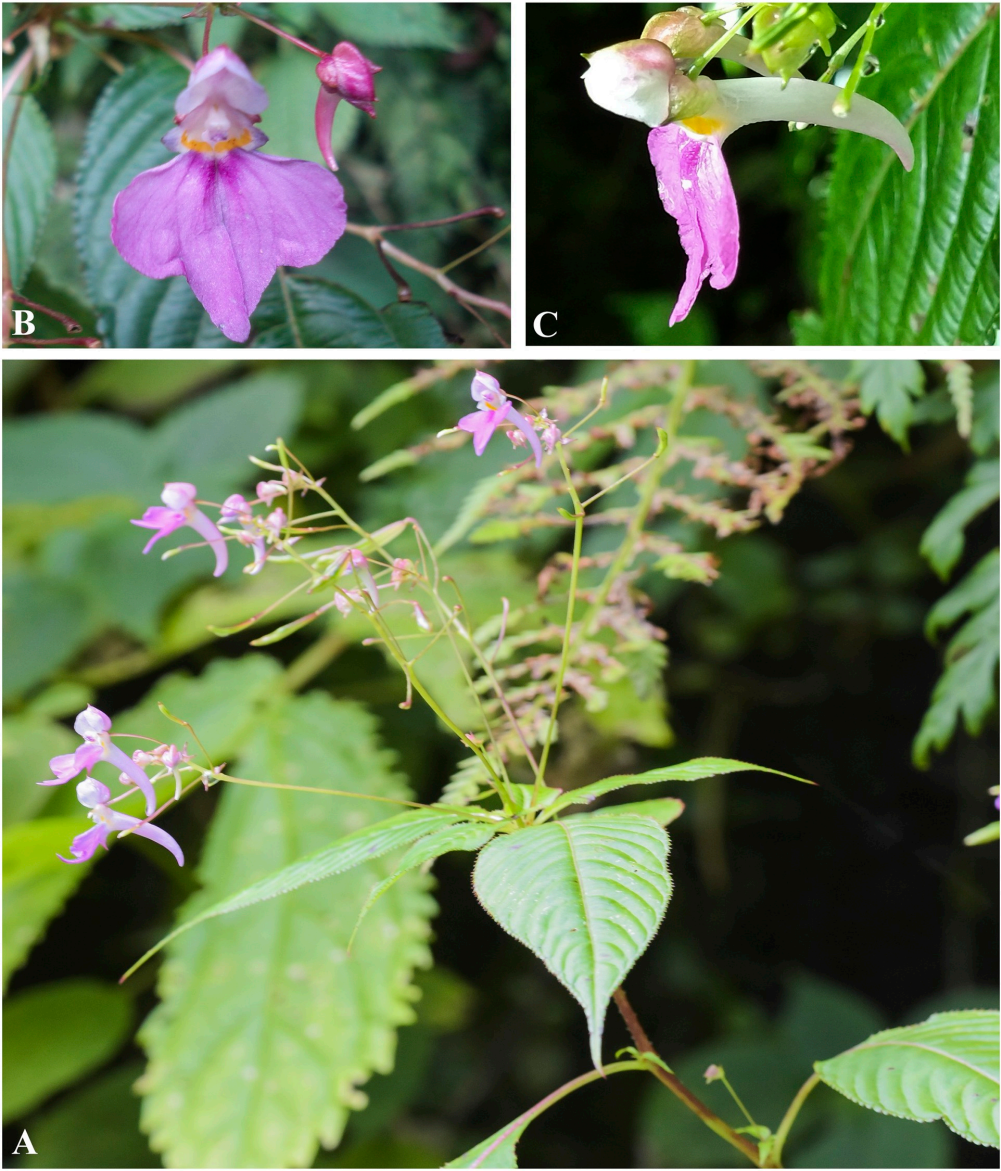
*Impatiens scullyi* A. Flowering plant; B. Flower (Front view); C. Flower (side view). Photographs by Bhakta Bahadur Raskoti.

***Impatiens scullyi*** Hook. f., Rec. B. Surv. Ind. 4: 15 & 21 (1905). Fig 24.

Type: *Scully J*. *272* (holo, K000694939!), Central Nepal.

Habitat: Temperate forest at an elevation of 2300 m.

Distribution: Eastern, Central, Western Nepal (endemic).

Specimen examined: Manang district, Thanchok-Dhangyung, 2400 m, 06 Aug. 1983, *Rajbhandari K*.*R*. *8993* (KATH); Bajhang district, Jimkot-Meltadi-Khaptad National Park, 1650 m, 26 Aug. 1991, *Suzuki M*. *et al*. *9170957* (TI); Bajura district, Kaudegaon-Babali, 1500 m, 29 Aug. 1991 *Suzuki M*. *et al*. *9171053* (TI); Kaski district, Ghandruk-Bhainsi Kharka, 2250 m, 22 Aug. 1988, *Suzuki M*. *et al*. *8881141* (TI); Manang district, Danake, 14 Aug. 1994, *Mikage M*. *et al*. *9485411* (TI); Mustang district, Kabre-Kalopani, 2300 m, 28 Aug. 1988, *Suzuki M*. *et al*. *8881529* (TI); Myagdi district, Shika-Narchang, 1460 m, 26 Aug. 1988, *Suzuki M*. *et al*. *8881450* (TI); Rasuwa district, Dhunche-Syabru, 29 Aug. 1991, *Noshiro S*. *9154506* (TI); Lingju-Tibling, 2100 m, 13 Aug. 1994, *Miyamoto F*. *et al*. *9420261* (TI).

**Fig 25:**
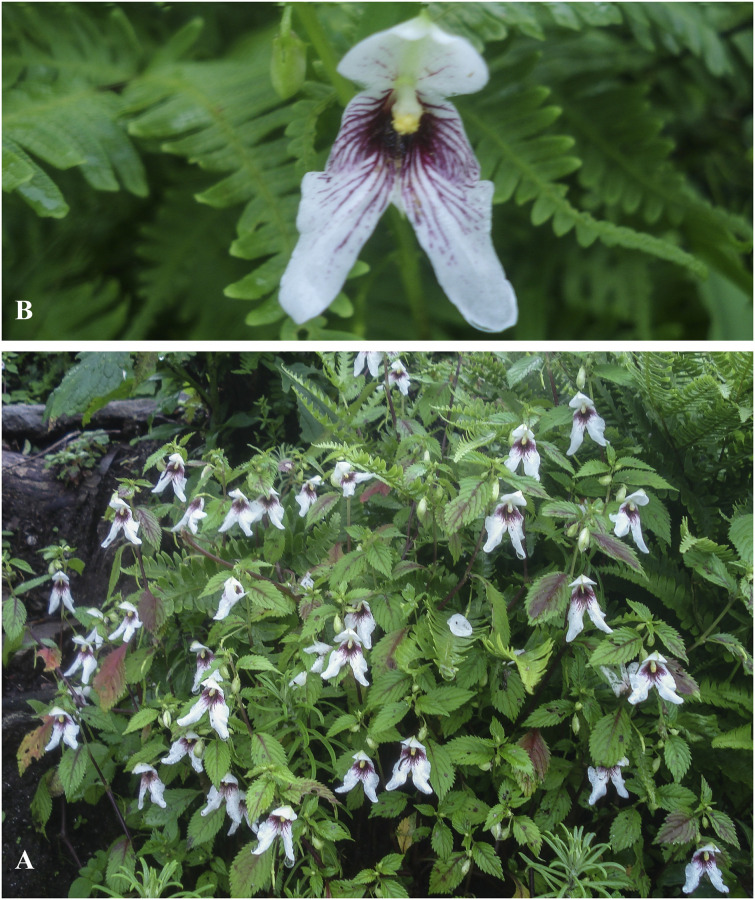
*Impatiens serrata* A. Flowering plant; B. Flower (Front view). Photographs by Bhakta Bahadur Raskoti.

**Impatiens serrata** Benth. ex Hook. f. & Thomson, J. Linn. Soc. Bot. 4: 136 (1860). Fig 25.

Type: *Wallich cat*. *no*. *4771* (lecto, designated in [29], larger middle specimen of K K001039866!; isolecto other specimens of CAL, K K001039866!), Nepal, 1821.

Habitat: Temperate forest at an elevation range of 2000–3600 m.

Distribution: Central, Western Nepal, Bhutan and China.

Specimen examined: Lamjung district, Tarma-Qubara, 3528 m, 03 Sept. 2021, *Thapa T*.*K*. *& Magar R*. *20210939* (KATH); Bajhang district, Jimkot-Meltadi-Khaptad National Park, 2850 m, 26 Aug. 1991, *Suzuki M*. *et al*. *9170959* (TI); Kalikot district, Panipokhari–Chaukebada, 2800 m, 04 Aug. 1991, *Suzuki M*. *et al*. *9170231* (TI); Rasuwa district, Chyauche Kharka-Lingju, 3580 m, 12 Aug. 1994, *Miyamoto F*. *et al*. *9420242* (TI); Rasuwa district, Deolari–Sing Gomba, 22 Jul. 1995, *Hoshino T*. *et al*. *9539060* (TI); Sindhupalchok district, Jangdang Kharka-Tingoang, 3100 m, 24 Sept. 1970, *Kanai H*. *et al*. *672949* (TI); Sindhupalchok district, Thale–Thale Bisauna, 2600 m, 10 Sept. 1970, *Kanai H*., *Ch*. *Chuma & T*. *Nagano 675182* (TI).

**Fig 26:**
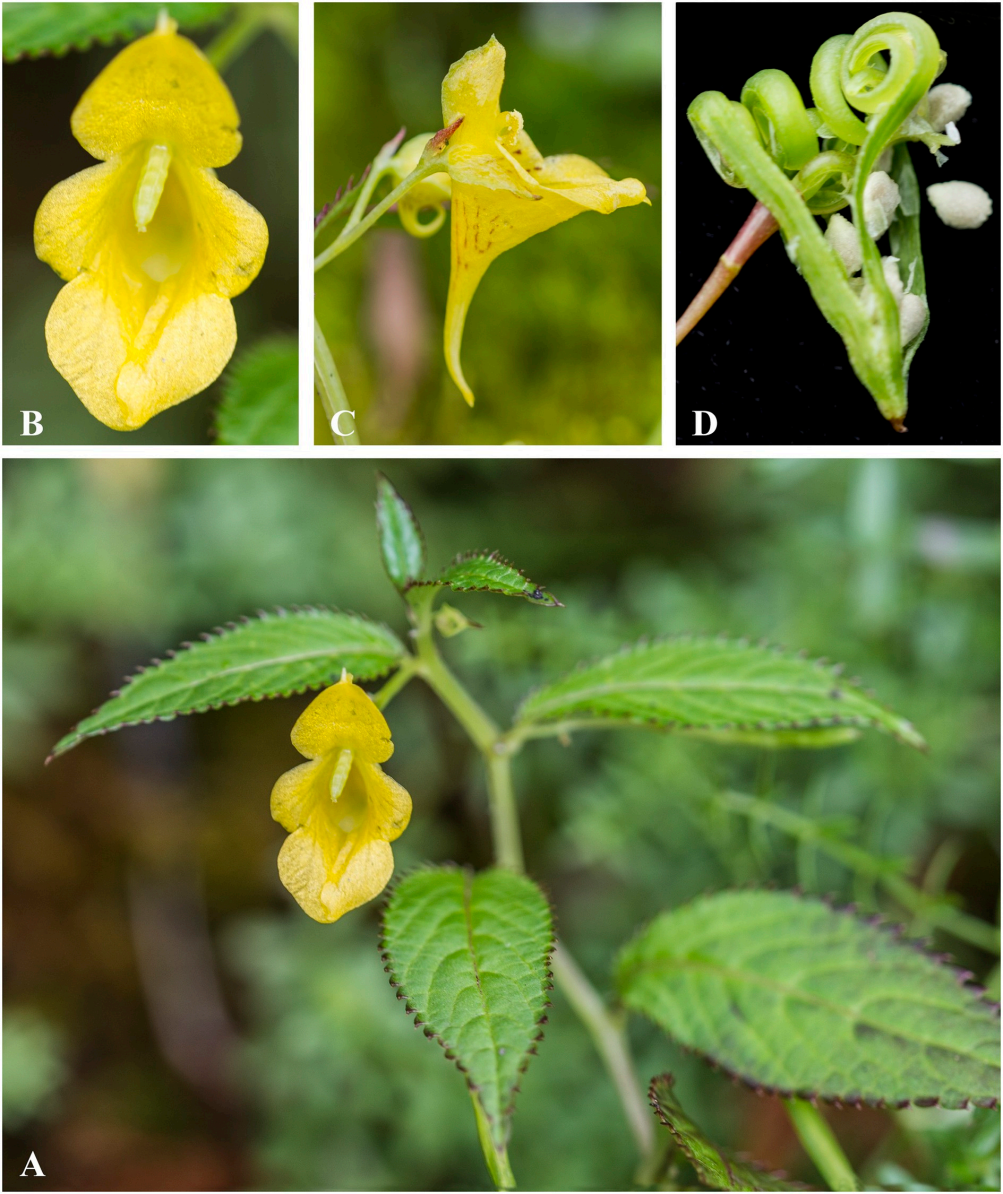
*Impatiens serratifolia* A. Flowering plant; B. Flower (Front view); C. Flower (side view); D. Capsule and seeds. Photographs by Bhakta Bahadur Raskoti.

***Impatiens serratifolia*** Hook. f., Rec. B. Surv. Ind. 4: 18 & 23 (1905). Fig 26.

Type: *Hooker J*. *D*. (lecto, designated in [39], K K0006949380!), Sikkim, Lachong, 9000–10000 ft. 06 Aug. 1849.

Habitat: Temperate forest at an elevation range of 2400–3000 m.

Distribution: Eastern Nepal and India.

Specimen examined: Solukhumbu district, Sete, 2550 m, 19 Aug. 1985, *Ohba H*. *et al*. *61436* (KATH); Solukhumbu district, Pangkoma-Najing Dingma, 3050 m, 03 Aug. 1997, *Rajbhandari K*.*R*. *9740040* (KATH); Sankhuwasabha district, Arun Valley, Chyamtang, 8000 ft., 24 May 1956, *Stainton J*.*D*.*A*. *413* (BM); Kyapra to Phedi, Ghunsa Khola, 2700 m, 07 Sept. 1989, *KEKE 324* (E).

**Fig 27:**
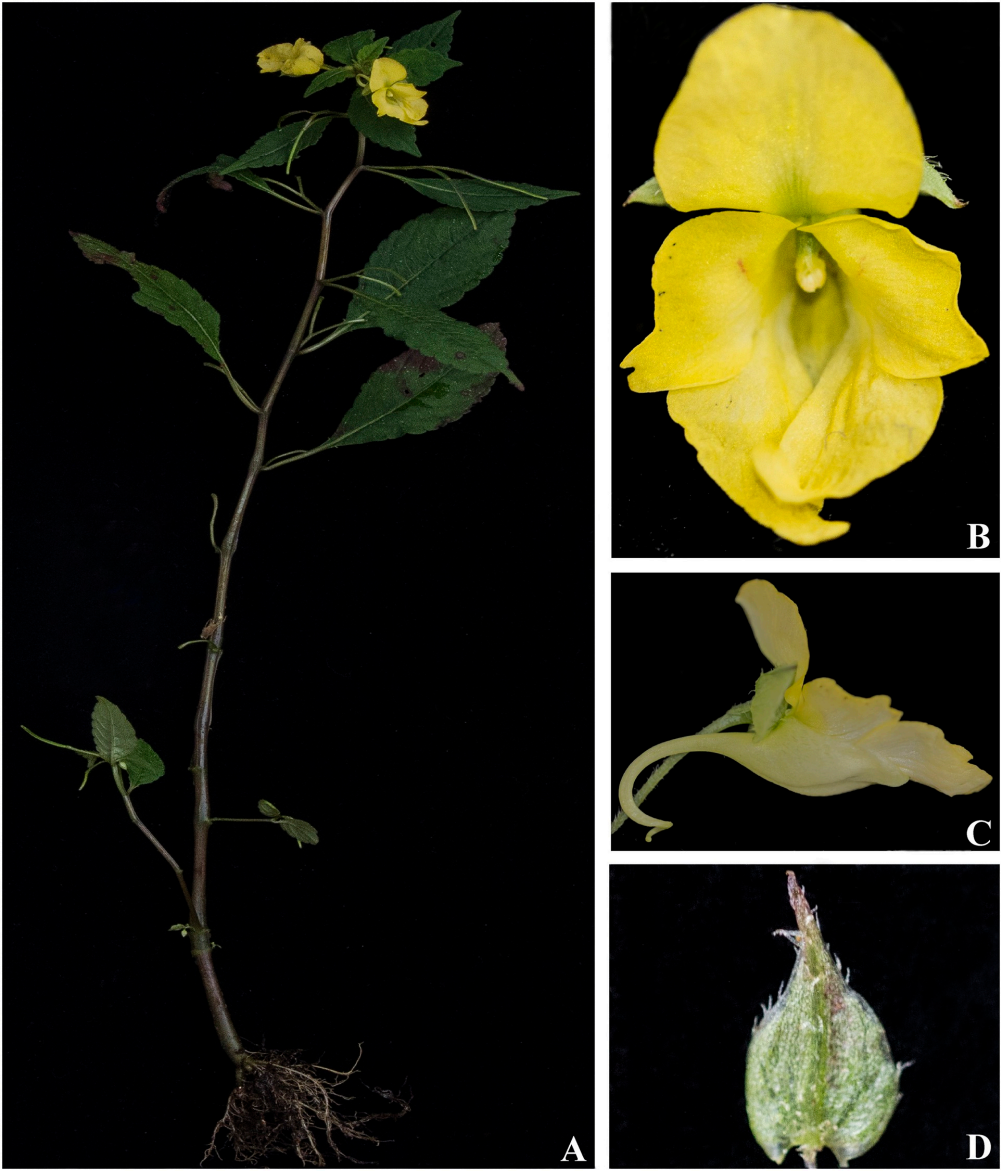
*Impatiens sikkimensis* A. Flowering plant; B. Flower (Front view); C. Flower (side view). Photographs by Bhakta Bahadur Raskoti.

***Impatiens sikkimensis*** Govaerts & Chakrabarty, Rheedea. 21(2):173 (2011). Fig 27.

Type: *Pantling s*.*n*., *Herb*. *Calc*. *1902* (lecto, designated in [40], K!, nr. 38/8-1-5B; isolecto, K!, CAL nr. 71551), Sikkim, 5000 ft., Dumsong.

Habitat: Subtropical forest at an elevation of 1400 m.

Distribution: Eastern Nepal and India.

Specimen examined: Ilam district, Kolbung, 1400 m, *Bhakta B*. *Raskoti 20145* (KATH).

***Impatiens spirifera*** Hook. f. & Thomson, J. Linn., Soc. Bot. 4: 135 (1859). Annotation as "spirifer"

Type: *Hooker J*. *D*. (lecto, designated in [41], K K000694933!), India, Sikkim, 4000–7000 ft.

Habitat: Open forest at an elevation range of 1400–2100 m.

Distribution: Eastern Nepal, China and India.

Specimen examined: Taplejung district, between Chirwa and Hellok, 1400 m, 04 Sept. 1989, *KEKE 200* (E); Panchthar district, Gairi Kharka–Beteni, 2000 m, 21 Jun. 1992, *Noshiro S*. *et al*. *9240987* (TI).

**Fig 28:**
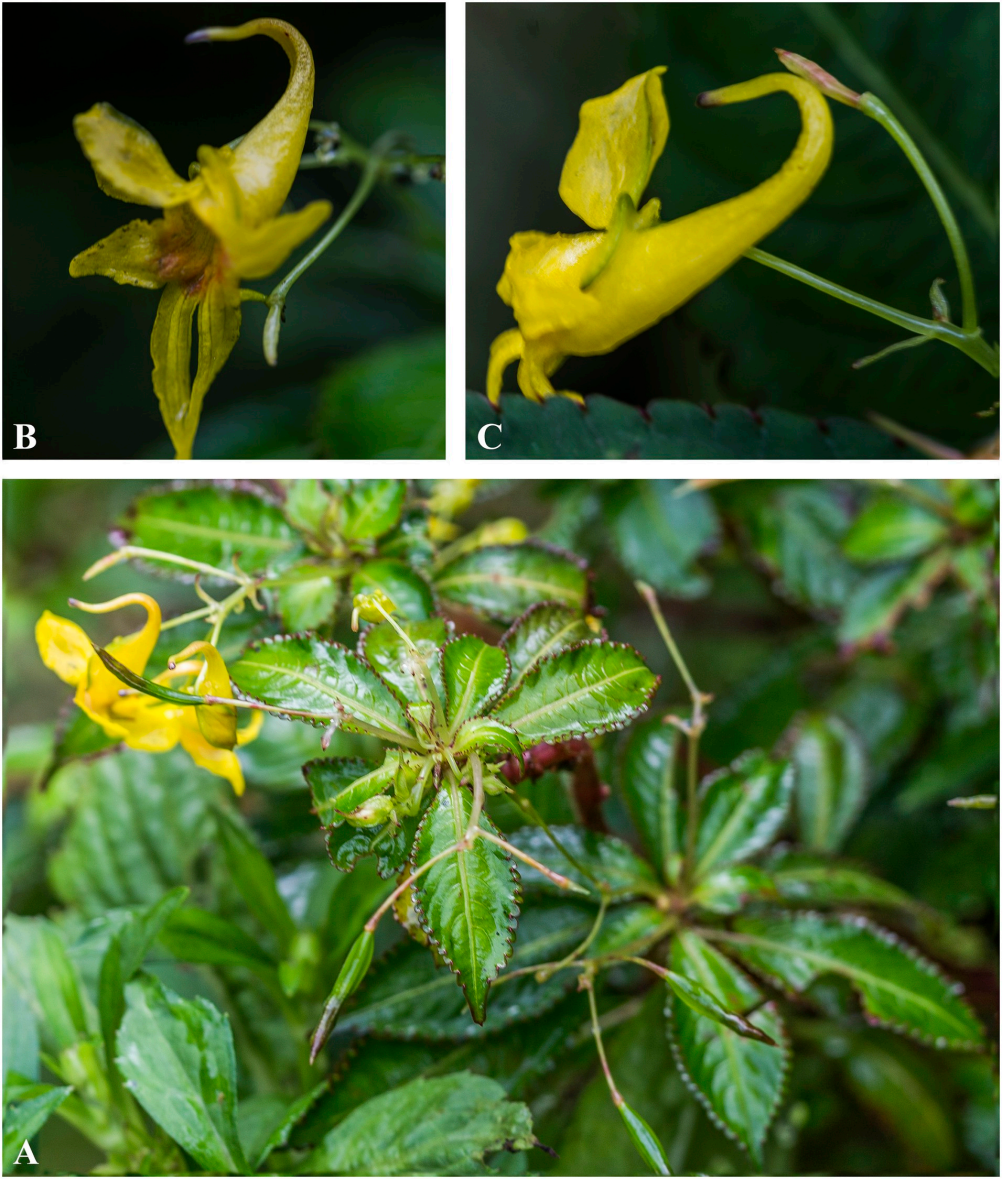
*Impatiens stenantha* A. Flowering plant; B. Flower (Front view); C. Flower (side view). Photographs by Bhakta Bahadur Raskoti.

***Impatiens stenantha*** Hook. f., Fl. Br. Ind. 1: 478 (1875). Fig 28.

Type: *Hooker J*.*D*. *s*.*n*. (lecto, designated in [29], larger upper specimen of K K000694611!, isolecto CAL, G00237315, smaller lower specimen of K K000694611!), India, Sikkim, 6000–8000 ft.

Habitat: Subtropical to temperate forest at an elevation range of 1800–2600 m.

Distribution: Eastern Nepal, Bhutan, China and India.

Specimen examined: Solukhumbu district, Bhotebas-Chichila, 1940 m, 20 Sept. 1991, *Long D*.*G*. *et al*. *108* (KATH); Dhankuta district, above Sedua, 7400 ft., 15 Aug. 1975, *Beer L*.*W*. *25308* (BM); Sedua-Thokpu, 2100 m, 25 Sept. 1971, *Shakya P*.*R*. *& Joshi D*.*P*. *919* (TI); Sankhuwasabha district, Tashi Gaun, 2250 m, 13 Jul. 1988, *Suzuki M*. *et al*. *8880424* (TI); Sankhuwasabha district, Arun Valley, Kasuwa Khola, 8500 ft., 11 Jun. 1956, *Stainton J*.*D*.*A*. *607* (BM); Panchthar district, Beteni-Chyangthapu, 2000 m, 22 Jun. 1992, *Noshiro S*. *et al*. *9240997* (TI); Panchthar district, Chyangthapu-Dabale Deurali, 2500 m, 23 Jun. 1992, *Noshiro S*. *et al*. *9241022* (TI); Taplejung district, Khokling-Thunglung, 1620 m, 12 May 1992, *Suzuki M*. *et al*. *9240047* (TI); Taplejung district, Mewa Khola-Papung, 2500 m, 29 Jun. 1972, *Kanai H*. *et al*. *720900* (TI).

**Fig 29:**
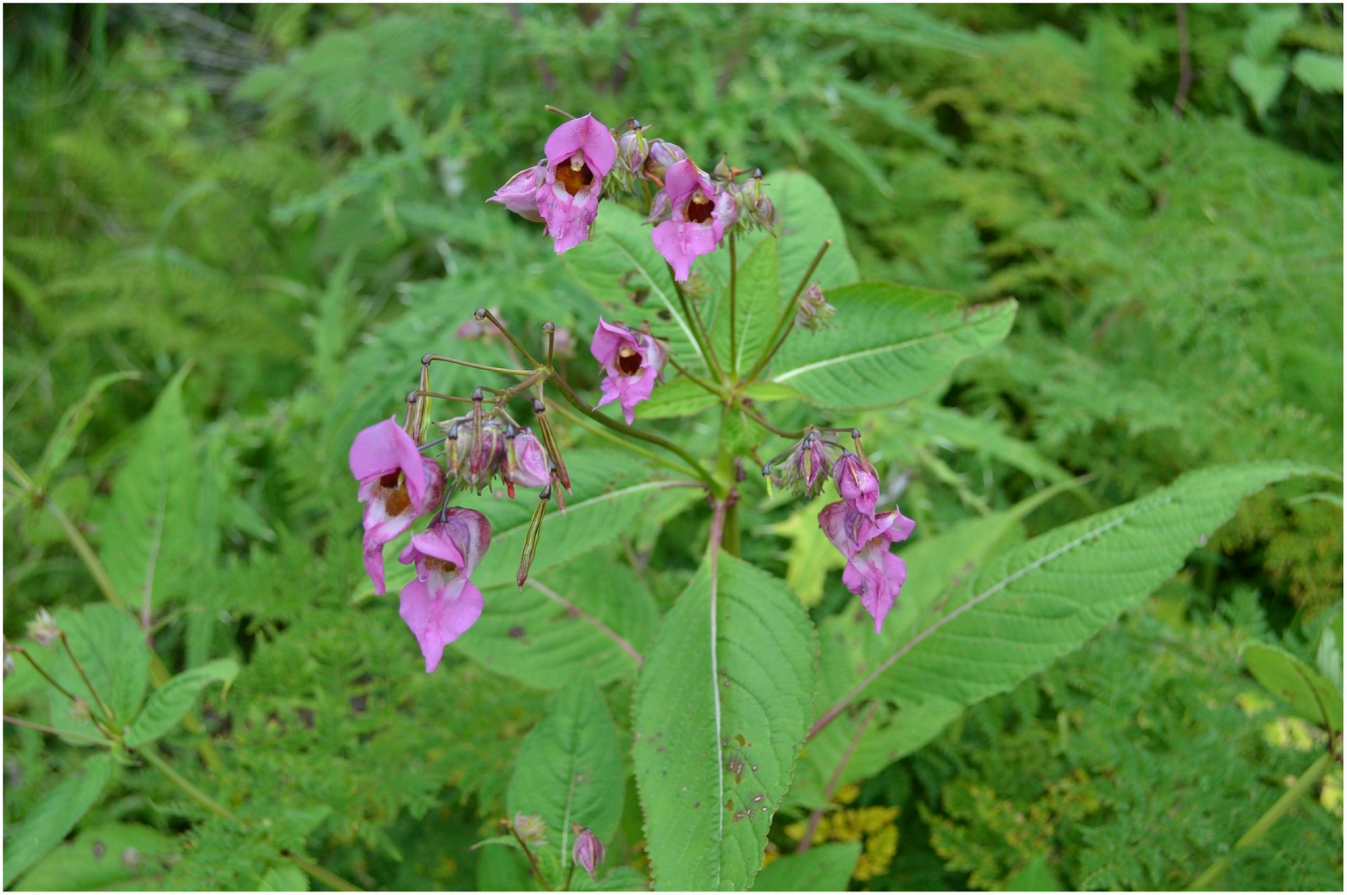
*Impatiens sulcata* (flowering plant). Photograph by Bhakta Bahadur Raskoti.

***Impatiens sulcata*** Wall., Roxb., Fl. Ind. 2: 458 (1824). Fig 29.

Type: *Wallich cat*. *no*. (lecto, designated in [29], *4764A* (K K001039846!)), Nepal, 1821.

*Impatiens gigantea* Edgew., Trans. Linn. Soc. London 20(1): 28 (1846).

Habitat: Subtropical forest to alpine grassland slopes at an elevation range of 1700–4100 m.

Distribution: Central, Western Nepal, Bhutan, China, India and Pakistan.

Specimen examined: Lamjung district, Thokyo-Nai Sima, 3429 m, 03 Sept. 2021, *Thapa T*.*K*. *& R*. *Magar 20210921* (KATH); Bajhang district, Khaptad National Park, 2900 m, 27 Aug. 1991, *Suzuki M*. *et al*. *9170984* (TI); Bajura district, Berseni–Porakya, 2650 m, 12 Aug. 1991, *Suzuki M*. *et al*. *9170486* (TI); Dolakha district, Charikot-Kalinchok, 9500 ft., 16 Sept. 1964, *Banerjee*, *Shrestha & Upadhya 2783* (US); Dolpa district, Balanga Pass, 11000 ft., 19 Jul. 1950, *Polunin*, *Sykes & Williams 2512* (E); Dolpa district, Jangla Banyang, 3800 m, 13 Jul. 1973, *Einarsson S*., *Skärby L*. *& Wetterhall B*. *2276* (BM); Ilam district, Samtar, 12000–13000 ft., *Lall Dhwoj 303* (E); Jumla district, Bhurchula Lehk, near Jumla, 12000 ft., 14 Jul. 1952, *Polumin O*., *Sykes W*.*R*. *& Williams L*.*H*.*J*. *4685* (BM); Kalikot district, Panipokhari-Chaukebada, 2680 m, 04 Aug. 1991, *Suzuki M*. *et al*. *9170232* (TI); Kaski district, Banthanti–Ghorapani, 3100 m, 24 Aug. 1988, *Suzuki M*. *et al*. *8881319* (TI); Manang district, Khangsar, 12500 ft., 15 Jul. 1950, *Stainton*, *Sykes & Williams 1194* (E); Manang district, Gunsang–Yak Kharka, 3720 m, 17 Aug. 1994, *Mikage M*. *et al*. *9470461* (TI); Mugu district, Ghurchi Lekh, between Lumra and Murma, 11000 ft., 11 Aug. 1952, *Polunin O*., *Sykes W*.*R*. *& Williams L*.*H*.*J*. *5121* (BM); Mustang district, Ghemi, 3520 m, 17 Aug. 2002, *Miyamoto F*. *et al*. *20230087* (TI); Mustang district, Ghenkar Gompa-Ghemi, 04 Aug. 2002, *Watanabe T*. *et al*. *s*.*n*. (TI).

**Fig 30:**
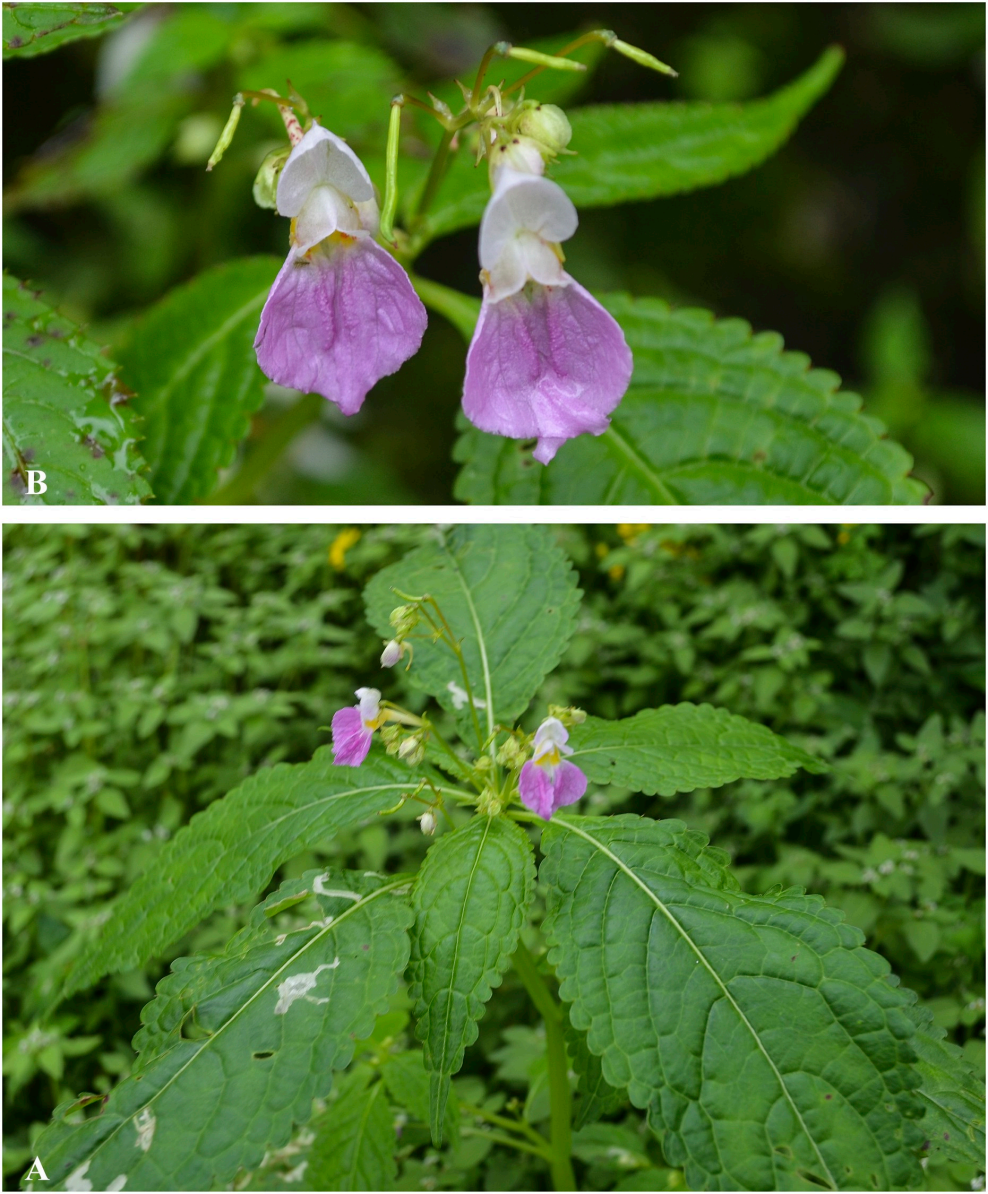
*Impatiens sunkoshiensis* A. Flowering plant; B. Flower (Front view). Photographs by Bhakta Bahadur Raskoti.

***Impatiens sunkoshiensis*** S. Akiyama, H. Ohba & Wakab., Himal. Pl. 2: 77 (1991). Fig 30.

Type: *Ohba H*. *et al*. *8520277* (holo, TI), Sagarmatha Zone, Sete-Taktor, 2550–3000 m, 19 Aug. 1985.

Habitat: Temperate forest at an elevation range of 2500–3200 m.

Distribution: Central Nepal (endemic).

Specimen examined: Gorkha distict, Ghopte to Namrung, 2240 m, 32 Jul. 2008, *Ikeda H*., *Kawahara T*., *Yano O*., *Watson M*.*F*., *Li Z*.*H*., *Subedi M*.*N*. *et al*. *20811095* (KATH).

Note: *Impatiens sunkoshiensis* is morphologically similar to *Impatiens laxiflora* but molecular analysis [6] indicated that these two taxa are two distinct species.

**Fig 31:**
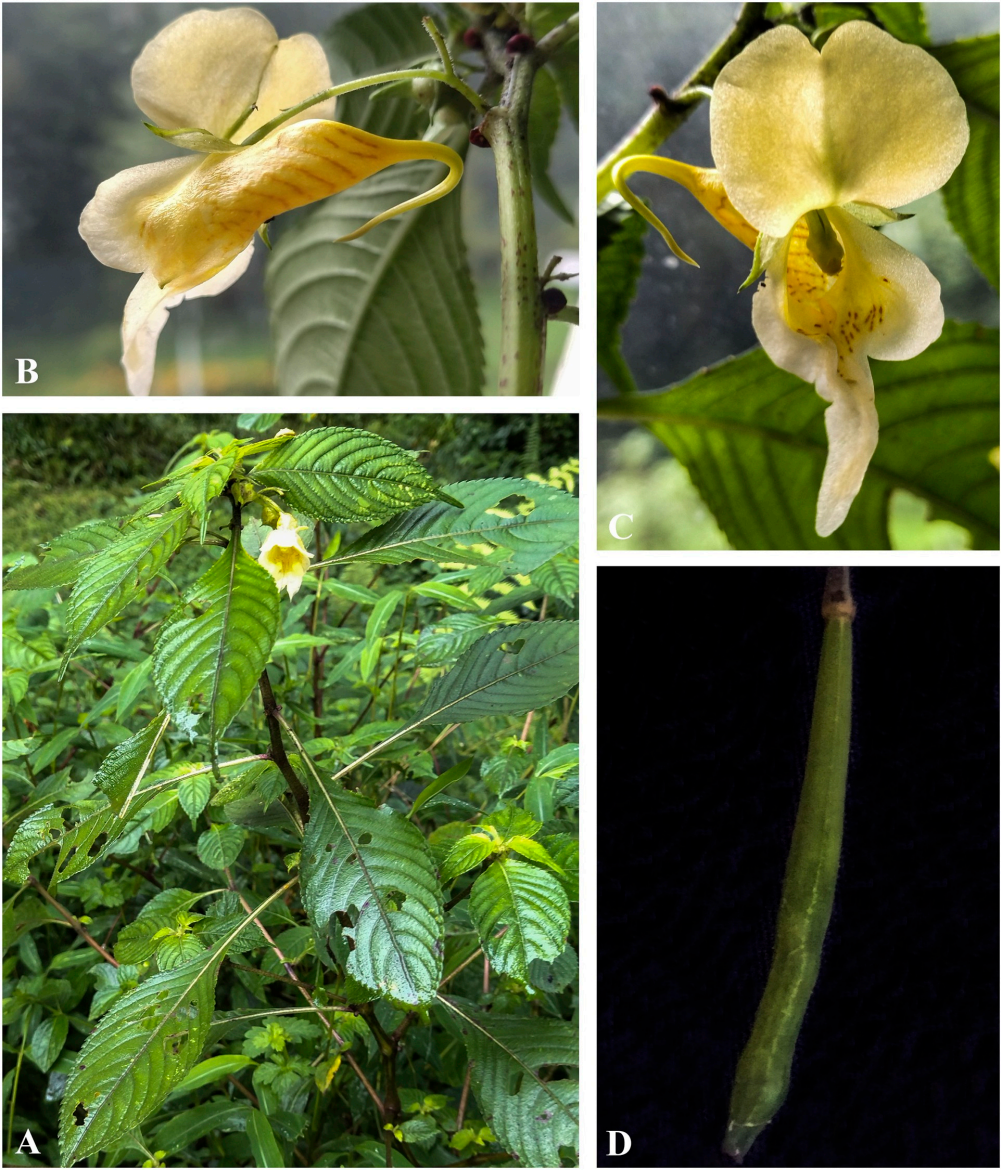
*Impatiens tricoris* A. Flowering plant; B. Side view of flower; C. Front view of flower; D. Capsule. Photographs by Bhakta Bahadur Raskoti.

***Impatiens tricornis*** Lindl., Bot. Reg. 26: t. 7 (1840). Fig 31.

Type: *Illustration*, *t*. *7 published in Bot*. *Reg*. *26 (1840)*, (lecto, designated in [39]).

*Impatiens praetermissa* Hook.f., J. Linn. Soc. Bot. 37: 29 (1904).

Type: *Wallich s*.*n*. (not found).

*Impatiens cristata* auct. non Wall.: Hook. f., Rec. Bot. Surv. India 4: 7 & 10 (1904); 22 (1905). Grey-Wilson, in Grierson and Long (eds.), Fl. Bhutan 2: 92, Fig 12a–12c (1991).

Distribution: Central, Western Nepal, China, India, Pakistan

Habitat: Subtropical to temperate forest and forest margins.

Specimen examined: Dadeldhura district, 2000 m, 05 May 1971, *Shakya P*.*R*. *& Joshi D*.*P*. *479* (TI); Bajhang district, Chainpur-Jimkot, 1200 m, 25 Aug. 1991, *Suzuki M*. *et al*. *9170946* (TI); Dolakha district, Gungur Khpla-Jagat, 1120 m, *Noshiro S*. *et al*. *20710213* (TI); Gorkha district, Syaule-Jagat, 1000 m, 23 Jul. 1994, *Suzuki M*. *et al*. *9470146* (TI); Jumla district, Munigaon-Chutta, 2850 m, 24 Jul. 1952, *Polunin*, *Sykes & Williams s*.*n*. (TI); Kalikot district, Chaukebada-Badarigaon, 05 Aug. 1991, *Suzuki M*. *et al*. *9170299* (TI); Kaski district, Tikhedhunga-Kande, 07 Sept. 1988, *Suzuki M*. *et al*. *8881795* (TI); Lalitpur district, Godavari, 18 Sept. 1963, *Hara H*. *et al*. *6306731* (TI); Makwanpur district, Deorali-Kuli Khani, 1700 m, 03 Sept. 1970, *Kanai H*. *& Shrestha T*.*B*. *672729* (TI); Manang, Latamanang, 2440 m, 14 Aug. 1994, *Mikage M*. *et al*. *9485416*, (TI); Myagdi district, Ghodepani Deurali-Shika, 2400 m, 25 Aug. 1988, Suzuki M. *et al*. 8881364, (TI); Rasuwa district, Dhunche-Syabru, 29 Aug. 1991, *Noshiro S*. *9154503* (TI).

**Fig 32:**
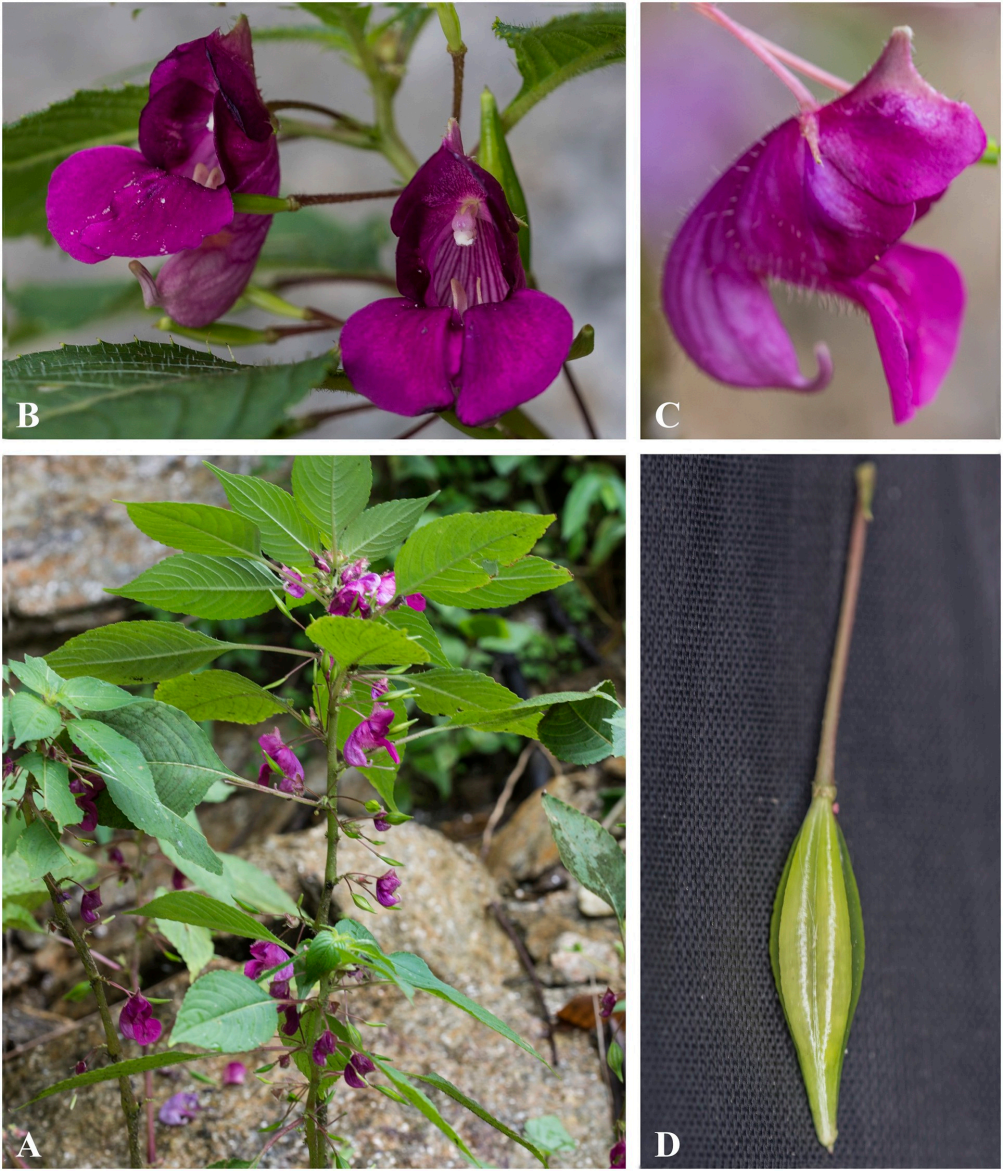
*Impatiens tripetala* A. Flowering plant; B. Flower (Front view); C. Flower (side view); D. Capsule. Photographs by Bhakta Bahadur Raskoti.

***Impatiens tripetala*** Roxb. ex DC., Prodr. 1: 687 (1824). Fig 32.

Type: ex hort. calc (lecto, designated in [42], G00218033), Silhet, India, Jul. 1818.

*Impatiens tripetala* Roxb. (1814) 18, nom. nud.; Wall. (1824) 453; G. Don (1831) 750; Roxb. (1832) 651; Hook.f. (1904a) 25, 27, 31; W.J. Kress *et al*. (2003) 170.

Type: *s*. *coll*. (lecto, designated in [42] G G00218033), India, 1818.

*Impatiens multiflora* Wall. ex Hook.f. & Thomson, Numer. List (Wallich) n. 4742 (1831).

Type: *De Silva*, *Wallich Cat*. *no*. 33*4742* (lecto, designated in [26], K K001039802!), Bangladesh, Pundua.

*Impatiens ternifolia* Buch.-Ham. ex Hook.f. & Thomson (1860) 126, nom. nud. *Impatiens ternifolia* Buch.-Ham. in Hook.f. (1904a) 27, nom. nud.,

Type: Based on: *Hb*. *Ham*., *Wall*. *Cat*. *4752B in Hooker (1904a) 27*, *31* (Syn, K K001039802!), India, Assam State, Goalpara, 08 May 1862.

Habitat: Subtropical forest at an elevation range of 1400–1800 m.

Distribution: Eastern Nepal, Bhutan, India and Myanmar.

Specimen examined: Taplejung district, between tawa and Chiruwa, 1200 m, 02 Aug. 1989, *Kew-Edinburgh-Kathmandu Expedition to N*.*E*. *Nepal 153* (E); Taplejung district, Tapethok, 05 Nov. 1963, *Hara H*. *et al*. *6300496* (TI); Taplejung district, Linkhim-Tuwa, 04 Nov. 1963, *Hara H*. *et al*. (TI); Taplejung district, Papung-Bir Gaon-Sangrati Pati, 1300 m, 26 Aug. 1977, *Ohashi H*. *et al*. *774018* (TI); Taplejung district, Saju Khola, 1400 m, 01 Jul. 1972, *Kanai H*. *et al*. *720981* (TI); Taplejung district, Taplethok, Tuwa-Kiwa-Tapethok, 05 Nov. 1963, *Hara H*. *et al*. *6300496* (KATH).

***Impatiens uncipetala*** C. B. Clarke ex Hook. f., Rec. B. Surv. Ind. 4: 18 & 22 (1905).

Type: *Clarke 9121* (lecto, designated in [43], K K000694903!), India, Darjeeling, Rungyroong, 6500 ft. 06 Sept. 1869.

*Impatiens yui* S.H. Huang, Acta Bot. Yunnan 25: 266 (2003).

Type: *Yü T*. *T*. *19964* (holo, KUN), China. Yunnan, Gongshan, Dulongjiang, 1800 m.

Habitat: Temperate forest at an elevation range of 2200–2400 m.

Distribution: Eastern Nepal, China and India.

Specimen examined: Dhankuta district, Sinduwa, 2100 m, 24 Oct. 1963, *Hara H*. *et al*. *6306721* (TI); Panchthar district, Chyangthapu–Dabale Deurali, 2500 m, 23 Jun. 1992, *Noshiro S*. *et al*. *9241023* (TI); Sankhuwasawa district, Tinjure Phedi-Mangal Bare, 15 Jul. 1991, *Ohba H*. *et al*. *9120059* (TI); Taplejung district, Surke Pati-Gupha Pokhari, 30 Aug. 1977, *Ohashi H*. *et al*. *774134* (TI).

***Impatiens urticifolia*** Wall., Roxb., Fl. Ind. 2: 457 (1824); in Cat. 168, n. 4768 (1831). Figs 4B and 15B.

Type: *Wallich 4768* (lecto, designated in [11], K-W [K001039858!]), Nepal, Gosain Than.

Habitat: Temperate to subalpine forest at an elevation range of 2700–3800 m.

Distribution: Eastern, Central, Western Nepal, Bhutan, China and India.

Specimen examined: Rasuwa district, Yure Kharka-Timbe Kharka, 3700 m, 26 Jul. 1994, *Rajbhandari K*.*R*. *17141* (KATH); Solukhumbu district, Hinku Khola, 3170 m, 27 Aug. 1995, *Miyamoto et al*. *9596491* (KATH); Bajura district, Pategaon-Badigaon, 3100 m, *Suzuki et al*. *9170640* (TI); Dhankuta district, Tinjure Phedi-Tinjure, 2600 m, *Kanai et al*. *721110* (TI); Kalikot district, Panipokhari-Chaukebada, 2690 m, *Suzuki et al*. *9170225* (TI); Kaski district, Banthanti-Ghodepani Deurali, 2660 m, *Suzuki et al*. *8860649* (TI); Panchthar district, Phalut, 3600 m, *Kanai et al*. *723188* (TI); Rasuwa district, Deurali-Sing Gomba, *Hoshino et al*. *9536049* (TI); Sankhuwasawa district, Bhainsi Kharka-Khongma, 2900 m, *Suzuki et al*. *8850385* (TI); Solukhumbu district, Dudh Kund-Thasing Dingma, 3305 m, *Miyamoto et al*. *9596491* (TI).

***Impatiens wallichii*** Hook. f., Rec. B. Surv. Ind. 4: 15 & 20 (1905). Fig 5C.

Type: *Wallich 4767* (lecto, designated in [11], upper right and left branches, K-W K001039857!), Nepal.

Habitat: Temperate forest to alpine grasslands at an elevation range of 3000–4100 m.

Distribution: Central Nepal and India.

Specimen examined: Rasuwa district, Pabil Kharka, 3200 m, 07 Aug. 1994, *Miyamoto F*. *et al*. *9420193* (KATH); Bhairabkunda, 4100 m, *Maire s*.*n*. (BM); Mustang district, Kali Gandaki, Larjung, 10000 ft., *Stainton et al*. *1951* (E); Mustang district, Kali Gandaki, Taglund, 12000 ft., *Stainton et al*. *1756* (E); Lamjung district, Lamjung Himal, 13500 ft., *Stainton et al*. *6353* (E); Rasuwa district, Chyauche Kharka-Lingju, 3730 m, Miyamoto *et al*. 9420240 (TI); Rasuwa district, Pabil Kharka-Seto Kund, 3550 m, *Miyamoto et al*. *9420193* (TI).

***Impatiens williamsii*** H. Hara, J. Jap. B. 47: 142 (1972).

Type: *Polunin*, *Sykes & Williams 5041* (holo, BM), Nepal, between Jumla and Garjigoth, 1000 ft. 08 Aug. 1952.

Habitat: Temperate forest at an elevation range of 2400–3100 m.

Distribution: Western Nepal (endemic).

Specimen examined: Hurta, Nagdungkhola, 8000 ft., 06 Oct. 1952, *Polunin O*., *Sykes W*.*R*. *& Williams L*.*H*.*J*. *3225* (KATH); 1000 ft., 15 Sept. 1952, *Polunin O*., *Sykes W*.*R*. *& Williams L*.*H*.*J*. *3407* (BM).

## Discussion

The relationships in the resulting cladograms of *Impatiens* (Fig 1), generally agree with those recovered in the previous phylogenetic analyses [6–9, 12, 24]. *Impatiens* is divided into two major clades (Clade I and Clade II). Clade II is further divided into different subclades.

Based on nuclear (ITS) and chloroplast (*atpB*-*rbcL*) markers, phylogenetic analyses revealed a separate lineage of newly sampled species (Fig 1, Clade II/Subclade III) with BS_MP_ = 80%, PP = 1.00, which is strongly supported as sister to *I*. *harae* and its allies (BS_MP_ = 90%, PP = 1.00). Taxa belonging to newly sampled species and their closely related clade (*I*. *harae*, *I*. *radiata*, *I*. *racemosa*, *I*. *urticifolia*, *I*. *wallichii*) share similar morphological characters such as many-flowered, racemose inflorescences, lateral sepals 2, rarely 4 with inner 2 reduced; capsule linear; seed ovoid.

*Impatiens bicornuta* also shares similar morphological characters with the taxa belonging to newly sampled clade. But in the present molecular analysis as well as in previous studies of *Impatiens* [6], *I*. *bicornuta* forms a separate lineage as sister to *I*. *cymbifera*, *I*. *purpurea* and *I*. *blinii*. There are different specimens identified as *I*. *bicornuta* deposited in different herbaria (such as K, E, TI) having variations in shape of leaves, petioles, inflorescence, color of flowers, peduncles, pod etc. Similarly, such variations can be observed in the natural populations in different habitats. In the literature [29] all these polymorphic taxa were treated as *I*. *bicornuta* and synonymized several species such as *I*. *harae*, *I*. *arunensis* based on only morphological observation. In this study, we did not accept treatment by [29] because of separate phylogenetic position of *I*. *bicornuta* and *I*. *harae*, based on molecular result (Fig 1). There may be existence of cryptic speciation in *I*. *bicornuta* complex group. More robust molecular study (specially population level) is needed to confirm taxonomic position and identity of *I*. *bicornuta* having different morphology in different population.

## Conclusions

With the discovery of this new species and five additional species new records to Nepal, a total 57 species of *Impatiens* (8 endemic species) were confirmed in the updated checklist of Balsaminaceae in Nepal. We strongly recommend that the future authors should integrate morphological and molecular data for the recognition and description of new species of *Impatiens*. Furthermore, we propose more robust studies (sampling population level) for the clarification of taxonomic position of *I*. *bicornuta* as well as taxonomic identity of its different polymorphic forms.

## Supporting information

S1 Fig Bayesian inference tree generated from ITS marker. Numbers above branches indicate bootstrap value for posterior probabilities (PP) for BI analysis. Asterisk (*) indicates PP = 1.00, a dash (−) indicates support at a node < 50%.
(PDF)

S2 Fig Bayesian inference tree generated *atpB-rbcl*. Numbers above branches indicate bootstrap value for posterior probabilities (PP) for BI analysis. Asterisk (*) indicates PP = 1.00, a dash (−) indicates support at a node < 50%.
(PDF)

S1 Table Taxa analyzed, voucher information and GenBank accession numbers for the DNA sequences. (DOC)
