# Supplementary figures and images for: A new species of Impatiens and updated checklist of Balsaminaceae in Nepal
Source: PLoS One. 2022 Oct 19;17(10):e0274699. doi: 10.1371/journal.pone.0274699 (PMC9581419; doi:10.1371/journal.pone.0274699)

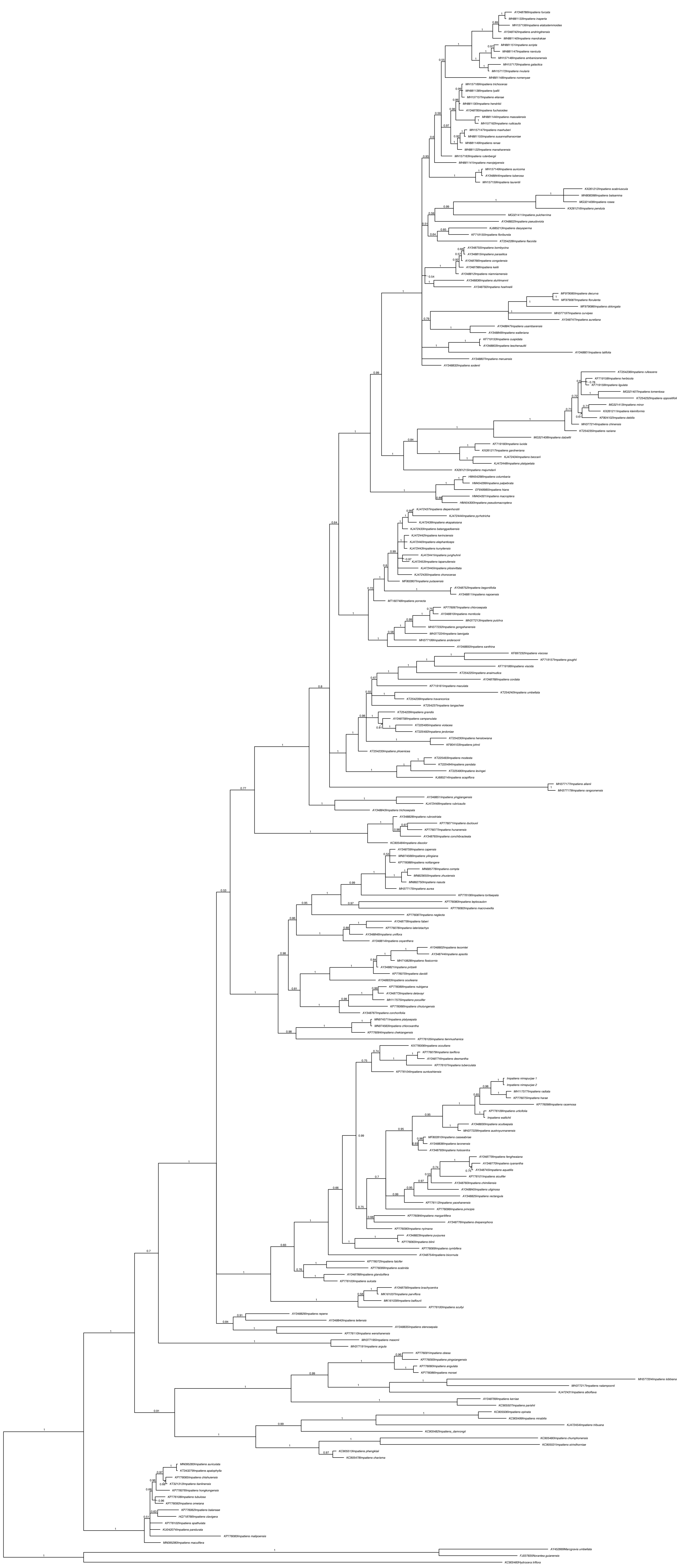

Supplement: S1 Fig — Numbers above branches indicate bootstrap value for posterior probabilities (PP) for BI analysis. Asterisk (*) indicates PP = 1.00, a dash (−) indicates support at a node < 50%. (PDF) [file pone.0274699.s001.pdf]

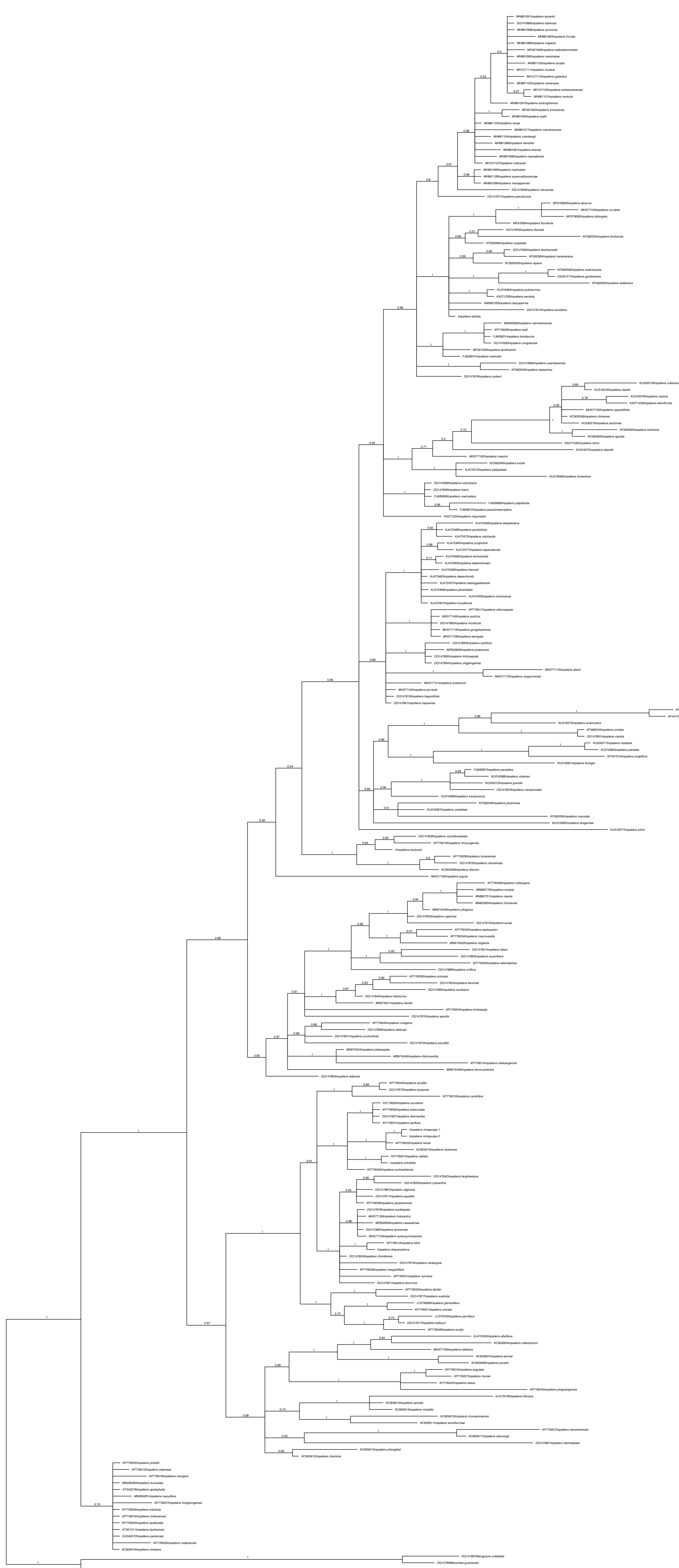

Supplement: S2 Fig — Numbers above branches indicate bootstrap value for posterior probabilities (PP) for BI analysis. Asterisk (*) indicates PP = 1.00, a dash (−) indicates support at a node < 50%. (PDF) [file pone.0274699.s002.pdf]
