# Supplementary material for: A new species of Impatiens and updated checklist of Balsaminaceae in Nepal
Source: PLoS One. 2022 Oct 19;17(10):e0274699. doi: 10.1371/journal.pone.0274699 (PMC9581419; doi:10.1371/journal.pone.0274699)
Supplement: S1 Table — (DOC) [file pone.0274699.s003.doc]

**­­S1Table. Taxa analyzed, voucher information, and GenBank accession numbers for the DNA sequences.**

| **Species name** | **ITS** | ***atpb-rbcL*** |
| --- | --- | --- |
| *Impatiens alboflava* | KJ472431 | KJ472455 |
| *Impatiens allanii* | MH377177 | MH377174 |
| *Impatiens ambanizanensis* | MH157148 | MH157103 |
| *Impatiens anaimudica* | KT254225 | KU316372 |
| *Impatiens andersonii* | MH377199 | MH377151 |
| *Impatiens andringitrensis* | AY348742 | MH881067 |
| *Impatiens angulata* | KP776060 | KP776010 |
| *Impatiens apalophylla* | KT343279 | KT343278 |
| *Impatiens apsotis* | AY348744 | DQ147810 |
| *Impatiens aquatilis* | AY348745 | DQ147811 |
| *Impatiens arguta* | MH377191 | MH377160 |
| *Impatiens aurea* | MH377175 | DQ147813 |
| *Impatiens aureliana* | AY348747 | DQ147814 |
| *Impatiens auricoma* | MH157149 | MH881068 |
| *Impatiens auriculata* | MN095283 | MN095284 |
| *Impatiens austroyunnanensis* | MH377229 | MH377123 |
| *Impatiens balansae* | KP776062 | KP776012 |
| *Impatiens balfourii* | MK161039 | DQ147817 |
| *Impatiens balsamina* | MH808398 | KF582043 |
| *Impatiens batanggadisensis* | KJ472433 | KJ472457 |
| *Impatiens beccarii* | KJ472434 | KJ472458 |
| *Impatiens begoniifolia* | AY348752 | DQ147819 |
| *Impatiens bicornuta* | AY348754 | DQ147821 |
| *Impatiens blinii* | KP776063 | KP776013 |
| *Impatiens bombycina* | AY348755 | FJ826631 |
| *Impatiens brachycentra* | AY348756 | - |
| *Impatiens campanulata* | AY348758 | DQ147822 |
| *Impatiens capensis* | AY348759 | DQ147823 |
| *Impatiens casseabriae* | MF802810 | MF802809 |
| *Impatiens charisma* | KC905478 | KC905612 |
| *Impatiens chekiangensis* | KP776064 | KP776014 |
| *Impatiens chimiliensis* | AY348760 | DQ147824 |
| *Impatiens chinensis* | MH377214 | KC905558 |
| *Impatiens chishuiensis* | KP776065 | KP776015 |
| *Impatiens chiulungensis* | KP776066 | KP776016 |
| *Impatiens chlorosepala* | KP776067 | KP776017 |
| *Impatiens chloroxantha* | MN974563 | MN974546 |
| *Impatiens chonoceras* | KJ472435 | KJ472459 |
| *Impatiens chumphonensis* | KC905480 | KC905610 |
| *Impatiens clavigera* | HQ718766 | KP776018 |
| *Impatiens columbaria* | HM454298 | DQ147828 |
| *Impatiens compta* | MN685778 | MN685776 |
| *Impatiens conchibracteata* | AY348765 | DQ147829 |
| *Impatiens congolensis* | AY348766 | DQ147830 |
| *Impatiens corchorifolia* | AY348767 | DQ147831 |
| *Impatiens cordata* | AY348768 | KF582044 |
| *Impatiens curvipes* | MH377197 | MH377153 |
| *Impatiens cuspidata* | KF719153 | KF562060 |
| *Impatiens cyanantha* | AY348770 | DQ147833 |
| *Impatiens cymbifera* | KP776069 | KP776019 |
| *Impatiens dalzellii* | MG321408 | KU316375 |
| *Impatiens damrongii* | KC905482 | KC905617 |
| *Impatiens dasysperma* | KJ685213 | KM360163 |
| *Impatiens davidii* | KP776070 | MN974551 |
| *Impatiens debilis* | KF804102 | KU316376 |
| *Impatiens decurva* | MF979085 | MF979082 |
| *Impatiens delavayi* | AY348773 | DQ147836 |
| *Impatiens desmantha* | AY348774 | DQ147837 |
| *Impatiens diepenhorstii* | KJ472437 | KJ472460 |
| *Impatiens discolor* | KC905484 | KC905568 |
| *Impatiens drepanophora* | AY348776 | MH377141 |
| *Impatiens duclouxii* | KP776071 | KC905569 |
| *Impatiens ekapaksiana* | KJ472439 | KJ472463 |
| *Impatiens elatostemmoides* | MH157156 | MF567403 |
| *Impatiens elephanticeps* | KJ472440 | KJ472464 |
| *Impatiens elianae* | MH157157 | MH881081 |
| *Impatiens faberi* | AY348778 | DQ147841 |
| *Impatiens falcifer* | KP776072 | KP776022 |
| *Impatiens fenghwaiana* | AY348779 | DQ147842 |
| *Impatiens fissicornis* | MH710828 | DQ147844 |
| *Impatiens flaccida* | KT254228 | DQ147845 |
| *Impatiens floribunda* | KF719155 | KF582045 |
| *Impatiens florulenta* | MF979087 | MF979084 |
| *Impatiens fuchsioides* | AY348785 | - |
| *Impatiens furcata* | AY348786 | MH881083 |
| *Impatiens galactica* | MH157170 | MH157114 |
| *Impatiens gardneriana* | KX261217 | KX261217 |
| *Impatiens glandulifera* | AY348788 | LC379698 |
| *Impatiens gongshanensis* | MH377232 | MH377119 |
| *Impatiens goughii* | KF719157 | KF447372 |
| *Impatiens grandis* | KT254229 | KU530215 |
| *Impatiens harae* | KP776075 | KP776025 |
| *Impatiens hendrikii* | MH881130 | MH881086 |
| *Impatiens henslowiana* | KT254230 | KF562064 |
| *Impatiens herbicola* | KF719158 | KF562065 |
| *Impatiens hians* | EF649985 | DQ147849 |
| *Impatiens hoehnelii* | AY348792 | FJ826647 |
| *Impatiens holocentra* | AY348793 | MH377139 |
| *Impatiens hongkongensis* | KP776076 | KP776027 |
| *Impatiens hunanensis* | KP776077 | KP776028 |
| *Impatiens inaperta* | MH881133 | MH881088 |
| *Impatiens jerdoniae* | KT225492 | KU530216 |
| *Impatiens johnii* | KF804103 | KU316377 |
| *Impatiens junghuhnii* | KJ472441 | KJ472465 |
| *Impatiens keilii* | AY348798 | KP776029 |
| *Impatiens kerinciensis* | KJ472442 | KJ472466 |
| *Impatiens kerriae* | AY348799 | KC905607 |
| *Impatiens kleiniformis* | KX261211 | KX271233 |
| *Impatiens kunyitensis* | KJ472443 | KJ472467 |
| *Impatiens laevigata* | MH377224 | MH377128 |
| *Impatiens lateristachys* | KP776078 | KP776030 |
| *Impatiens latifolia* | AY348801 | KU316378 |
| *Impatiens laurentii* | MH157159 | MH881091 |
| *Impatiens laxiflora* | KP776079 | KP776031 |
| *Impatiens lecomtei* | AY348802 | DQ147855 |
| *Impatiens leptocaulon* | KP776080 | KP776032 |
| *Impatiens leschenaultii* | AY348803 | DQ147856 |
| *Impatiens levingei* | KT225490 | KU316381 |
| *Impatiens ligulata* | KF719159 | KF562063 |
| *Impatiens lobbiana* | MH377204 | MH377169 |
| *Impatiens lucida* | KF719160 | KF582046 |
| *Impatiens lyallii* | MH881138 | MH881094 |
| *Impatiens macroptera* | HM454301 | FJ826658 |
| *Impatiens macrovexilla* | KP776082 | KP776034 |
| *Impatiens maculata* | KF719161 | KF562056 |
| *Impatiens maculifera* | MN095280 | MN095281 |
| *Impatiens majumdarii* | KX261215 | KX271234 |
| *Impatiens malipoensis* | KP776083 | KP776035 |
| *Impatiens manaharensis* | MH881122 | MH881077 |
| *Impatiens mandrakae* | MH881140 | MH881095 |
| *Impatiens margaritifera* | KP776084 | KP776036 |
| *Impatiens marojejyensis* | MH881141 | MH881096 |
| *Impatiens masoalensis* | MH881144 | MH881098 |
| *Impatiens masonii* | MH377195 | MH377162 |
| *Impatiens max-huberi* | MH157147 | MH881099 |
| *Impatiens meruensis* | AY348807 | DQ147859 |
| *Impatiens minor* | MG321413 | KX271235 |
| *Impatiens mirabilis* | KC905499 | KC905614 |
| *Impatiens modesta* | KT225493 | KU530217 |
| *Impatiens monticola* | AY348810 | DQ147860 |
| *Impatiens morsei* | KP776086 | KP776037 |
| *Impatiens nalampoonii* | MH377217 | KC905604 |
| *Impatiens napoensis* | AY348811 | DQ147861 |
| *Impatiens nasuta* | MN862750 | MN862751 |
| *Impatiens navicula* | MH881147 | MH881101 |
| *Impatiens neglecta* | KP776087 | MN974552 |
| *Impatiens niamniamensis* | AY348812 | MK840606 |
| *Impatiens noli-tangere* | KP776088 | KP776039 |
| *Impatiens nomenyae* | MH881148 | MH881102 |
| *Impatiens nubigena* | KP776089 | KP776040 |
| *Impatiens nyimana* | KP776090 | KP776041 |
| *Impatiens obesa* | KP776091 | KP776042 |
| *Impatiens oblongata* | MF979086 | MF979083 |
| *Impatiens occultans* | KX776006 | KX776003 |
| *Impatiens omeiana* | KP776092 | KC905619 |
| *Impatiens opinata* | KC905506 | KC905615 |
| *Impatiens oppositifolia* | KT254232 | MH377165 |
| *Impatiens oxyanthera* | AY348814 | DQ147865 |
| *Impatiens palpebrata* | HM454299 | FJ826666 |
| *Impatiens pandata* | KT225494 | KU316383 |
| *Impatiens pandurata* | KU042074 | KU042072 |
| *Impatiens parasitica* | AY348815 | FJ826667 |
| *Impatiens parishii* | KC905507 | KC905608 |
| *Impatiens parviflora* | MK161037 | LC379700 |
| *Impatiens pendula* | KX261216 | KX271230 |
| *Impatiens phengklaii* | KC905513 | KC905613 |
| *Impatiens phoenicea* | KT254233 | KF582049 |
| *Impatiens pilosivittata* | KJ472445 | KJ472469 |
| *Impatiens pingxiangensis* | KP776093 | KP776043 |
| *Impatiens platypetala* | KJ472448 | KJ472472 |
| *Impatiens platysepala* | MN974571 | MN974554 |
| *Impatiens poculifer* | MH117575 | DQ147870 |
| *Impatiens porrecta* | MT160748 | MH377140 |
| *Impatiens principis* | KP776096 | KP776026 |
| *Impatiens pritzelii* | AY348821 | KP776045 |
| *Impatiens pseudomacroptera* | HM454300 | FJ826670 |
| *Impatiens pseudoviola* | AY348822 | DQ147871 |
| *Impatiens pulcherrima* | MG321411 | KU316384 |
| *Impatiens pulchra* | MH377213 | MH377149 |
| *Impatiens purpurea* | AY348823 | DQ147872 |
| *Impatiens putaoensis* | MF802807 | MF802806 |
| *Impatiens pyrhotricha* | KJ472444 | KJ472468 |
| *Impatiens racemosa* | KP776098 | KC905574 |
| *Impatiens radiata* | MH117577 | KP776047 |
| *Impatiens rangoonensis* | MH377179 | MH377172 |
| *Impatiens raziana* | KT254235 | KU316379 |
| *Impatiens rectangula* | AY348825 | DQ147874 |
| *Impatiens renae* | MH881149 | MH881103 |
| *Impatiens repens* | AY348826 | KC905555 |
| *Impatiens rivularis* | MH157172 | MH157111 |
| *Impatiens rosea* | MG321409 | - |
| *Impatiens rubricaulis* | KJ472449 | KJ472473 |
| *Impatiens rubrostriata* | AY348828 | DQ147876 |
| *Impatiens rudicaulis* | MH157162 | MH157107 |
| *Impatiens rufescens* | KT254236 | KU530218 |
| *Impatiens rutenbergii* | MH157163 | MH881104 |
| *Impatiens scabrida* | KP776099 | DQ147877 |
| *Impatiens scabriuscula* | KX261212 | KF562058 |
| *Impatiens scapiflora* | KJ685214 | KF447374 |
| *Impatiens scripta* | MH881151 | MH881105 |
| *Impatiens scullyi* | KP776100 | KP776048 |
| *Impatiens scutisepala* | AY348830 | DQ147878 |
| *Impatiens siculifer* | KP776101 | KP776049 |
| *Impatiens sirindhorniae* | KC905531 | KC905611 |
| *Impatiens sodenii* | AY348832 | DQ147879 |
| *Impatiens soulieana* | AY348833 | DQ147880 |
| *Impatiens nimspurjae* | XXXXXX | XXXXXX |
| *Impatiens nimspurjae* | XXXXXX | XXXXXX |
| *Impatiens spathulata* | KP776102 | KP776050 |
| *Impatiens stenosepala* | AY348835 | DQ147881 |
| *Impatiens stuhlmannii* | AY348836 | MF567406 |
| *Impatiens sulcata* | KP776103 | KP776051 |
| *Impatiens sunkoshiensis* | KP776104 | KP776052 |
| *Impatiens susan-nathansoniae* | MH881155 | MH881109 |
| *Impatiens tangachee* | KT254237 | KU316385 |
| *Impatiens tapanuliensis* | KJ472453 | KJ472477 |
| *Impatiens taronensis* | AY348838 | DQ147882 |
| *Impatiens teitensis* | AY348840 | DQ147883 |
| *Impatiens tianlinensis* | KT321312 | KT321311 |
| *Impatiens tienmushanica* | KP776105 | MN974548 |
| *Impatiens tomentosa* | MG321407 | KU316386 |
| *Impatiens tortisepala* | KP776106 | KP776054 |
| *Impatiens travancorica* | KT254239 | KU316380 |
| *Impatiens tribuana* | KJ472454 | KJ472478 |
| *Impatiens trichoceras* | MH157169 | MF567420 |
| *Impatiens trichosepala* | AY348843 | DQ147885 |
| *Impatiens tuberculata* | KP776107 | KP776055 |
| *Impatiens tuberosa* | AY348844 | DQ147886 |
| *Impatiens tubulosa* | KP776108 | KP776056 |
| *Impatiens uliginosa* | AY348845 | DQ147887 |
| *Impatiens umbellata* | KT254240 | KU316387 |
| *Impatiens uniflora* | AY348846 | DQ147888 |
| *Impatiens urticifolia* | KP776109 | - |
| *Impatiens usambarensis* | AY348847 | DQ147889 |
| *Impatiens violacea* | KT225495 | KU316388 |
| *Impatiens viscida* | KF719166 | DQ147891 |
| *Impatiens viscosa* | KF697232 | KF447373 |
| *Impatiens walleriana* | AY348849 | KF582050 |
| *Impatiens wallichii* | XXXXXX | - |
| *Impatiens wenshanensis* | KP776110 | KP776057 |
| *Impatiens xanthina* | AY348850 | DQ147893 |
| *Impatiens yaoshanensis* | KP776112 | KP776059 |
| *Impatiens yilingiana* | MN974566 | MN974549 |
| *Impatiens yingjiangensis* | AY348851 | DQ147894 |
| *Impatiens zhuxiensis* | MN823655 | MN823654 |
| *Hydrocera triflora* | KC905460 | DQ147895 |
| *Marcgravia umbellata* | AY452669 | DQ147897 |
| *Norantea guianensis* | FJ037835 | DQ147898 |
